# Insights into widespread disturbance in gene expression and severe growth inhibition observed in transgenic rice producing polyhydroxybutyrate

**DOI:** 10.5511/plantbiotechnology.24.1107a

**Published:** 2025-03-25

**Authors:** Hiroaki Shimada, Astuo Kawamura, Miki Ogasawara, Aya Tamaki, Tetsuya Yamazaki, Yohei Igarashi, Sota Hara, Chiaki Yamagiwa, Hiroshi Teramura, Hiroaki Kusano, Ken’ichiro Matsumoto

**Affiliations:** 1 Department of Biological Science and Technology, Tokyo University of Science, Katsushika, Tokyo 125-8585, Japan

**Keywords:** cell death, defense response gene, gene ontology terms, polyhydroxyalkanoate, transcriptome analysis

## Abstract

Production of polyhydroxybutyrate (PHB), a kind of biodegradable polymer, was attempted using transformant rice, in which the genes involved in PHB biosynthesis in *Cupriavidus necator* were introduced. Accumulation of PHB was observed in the transformants containing the genes for β-ketothiolase (*phaA*), acetoacetyl-CoA reductase (*phaB*) and PHB synthase (*phaC*) (*PhaABC* lines) and those containing *phaB* and *phaC* (*PhaBC* lines). However, they immediately withered after regeneration due to severe growth inhibition, whereas no growth inhibition occurred in the *PhaAB* lines containing *phaA* and *phaB*, and the *PhaC* lines containing *phaC*, which did not produce PHB. Crossing between them generated sufficient quantities of F1 seeds. Many of them germinated and produced PHB, but they died at an early stage of growth. This suggests that the accumulation of PHB in the cells caused a strong growth inhibition. Microarray analysis using the *PhaBC* and *PhaC* lines revealed very similar expression profiles, suggesting that most of the changes in gene expression were mainly caused by *phaC* gene expression. *PhaC* transformants exhibited increased expression of genes involved in the stress response, certain biological processes and cellular components. These results strongly suggest that *phaC* gene expression results in perturbation of gene expression levels in various cell functions. It was concluded that the disturbance of cell function caused by *phaC* gene expression is enhanced by the intracellular production of PHB, leading to cell death.

## Introduction

Polyhydroxyalkanoates (PHAs) are synthesized as bacterial storage materials that can be processed into useful thermoplastic materials (Tang et al. 2022). These polyesters are a biobased and biodegradable alternative to petrochemical plastics. The polymers are industrially produced from plant-derived biomass, such as plant oils, sugars and glycerol, by bacterial fermentation. PHAs are used as carbon-neutral biodegradable plastics that reduce the environmental impact of nonbiodegradable plastic pollution (Acharjee et al. 2023; Atiwesh et al. 2021; Moshood et al. 2022).

**Figure 1:**
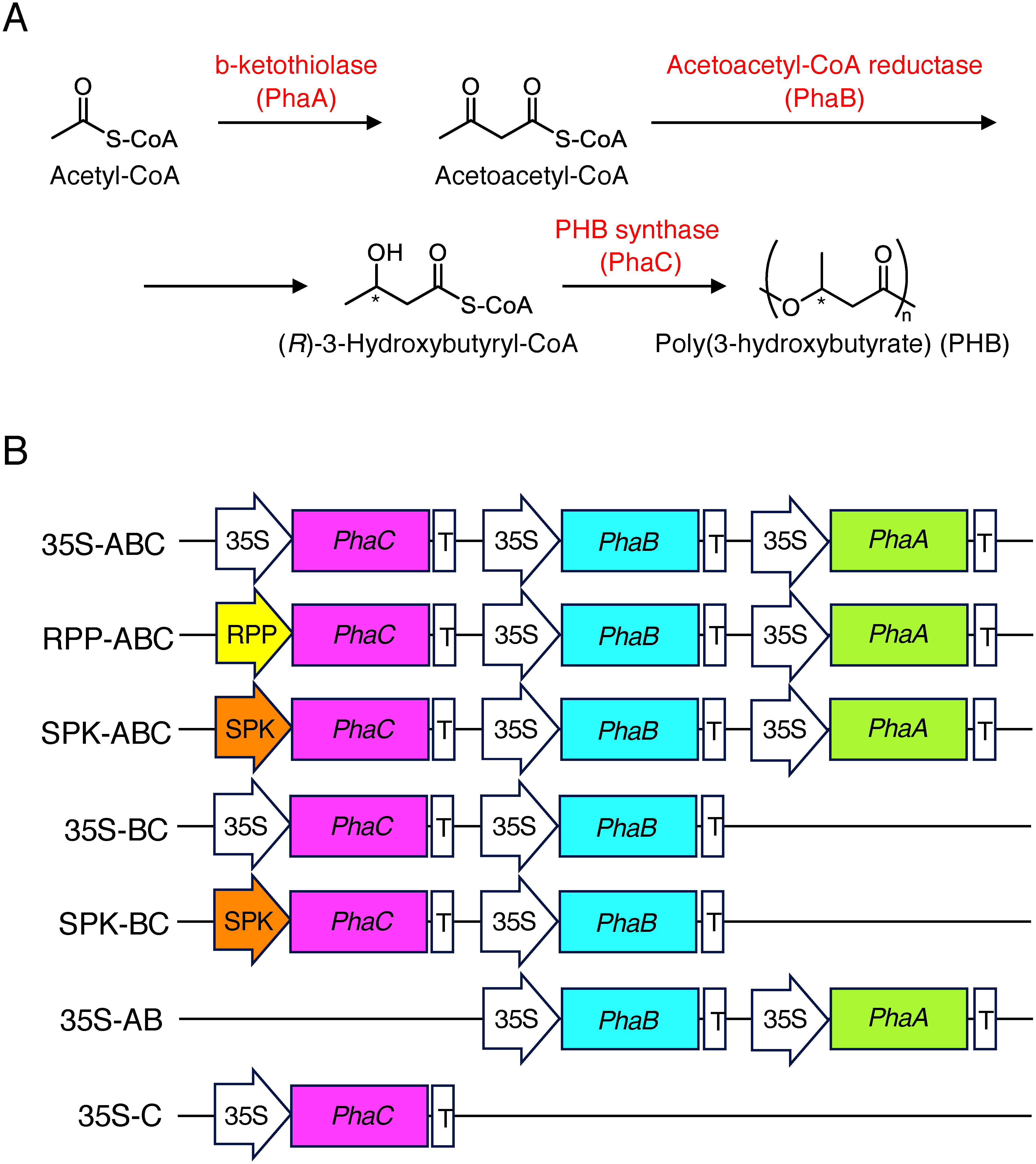
Figure 1. (A) Pathway of PHB biosynthesis. Enzyme names and the corresponding genes are shown. *An asymmetric carbon. (B) Structures of the plasmids used in this study, 35S: CaMV *35S* promoter, RPP: *RPP17* promoter, SPK: *SPK* promoter, *PhaA*, *PhaB*, and *PhaC*: protein coding regions of the *phaA*, *phaB* and *phaC* genes of *Cupriavidus necator*, T: nos terminator.

Poly(3-hydroxybutyrate) (PHB), a common PHA, is synthesized in bacteria from acetyl-CoA by the consecutive actions of β-ketothiolae (PhaA), acetoacetyl-CoA reductase (PhaB), and PHA synthase (PhaC) (Peoples and Sinskey 1989) (Figure 1A). Therefore, PHB can be produced in various hosts by transferring and expressing PHB biosynthetic genes.

Transgenic plants expressing microbial enzymes are potent platforms to produce value-added chemicals directly from carbon dioxide (Somleva et al. 2013). In planta, PHB production has a potential advantage in terms of cost over PHB production by fermentation because no carbon stocks or fermentation facilities are needed for plant growth (van Beilen and Poirier 2008). When PHB is synthesized in plants, genes for the enzymes involved in the synthesis of NADPH-dependent acetoacetyl-CoA reductase (*phaB*) and PHB synthase (*phaC*) must essentially be introduced, whereas it has been suggested that the plant cytosol contains β-ketothiolase activity (Poirier et al. 1992).

Several PHB-producing transgenic plants have been generated. The first PHB production in plants was achieved using *Arabidopsis thaliana* (Poirier et al. 1992). To date, many attempts have been made using rapeseed, tobacco, potato, sugarcane and switchgrass (Arai et al. 2004; Bohmert et al. 2000; Houmiel et al. 1999; Matsumoto et al. 2011; Mott et al. 2000; Petrasovits et al. 2007; Purnell et al. 2007; Romano et al. 2003, 2005; Slater et al. 1999; Somleva et al. 2008). However, it is known that PHB production is much lower in plant cytosol than in bacteria and that accumulation of PHB has a negative influence on plant growth (Yoshizumi et al. 2017). Therefore, an increase in PHB accumulation is an expected goal of plant PHB production.

We focused on rice, *Oryza sativa*, as a model plant that shows fast growth and high biomass production and attempted to create PHB-producing rice transformants. During the course of the experiments, we observed severe growth inhibition in PHB-accumulating rice, with a critical and precipitous phenotype, suggesting that a strong cytotoxicity occurred in the cell due to PHB synthesis. Here, to elucidate the mechanism of growth inhibition by PHB synthesis, we performed a comprehensive analysis and sought to identify the hazardous elements that cause growth inhibition in the transformant rice expressing *phaB* and *phaC* genes. Based on these findings, we discuss what occurred in the PHB-producing plants.

## Materials and methods

### Plasmid construction

The coding regions of the genes *phaA*, *phaB* and *phaC* of *Cupriavidus necator* (formerly *Ralstonia eutropha*) (Acc. nos. MH558939) were assembled for PHB production in rice. Each fragment was placed downstream of the cauliflower mosaic virus (CaMV) *35S* promoter or another appropriate promoter. For the tissue-specific expression of *phaC*, we used the promoters of *RPP17* (*Os01g0841700*) and *SPK* (*Os10g0539600*) genes encoding the phloem protein RPP17 and the protein kinase SPK, which are specifically expressed in vascular tissues and developing seeds, respectively, in addition to cultured cells (Asano et al. 2002a, 2002b). They were inserted into the binary vector pBI121 as described previously (Matsumoto et al. 2009) and subjected to the construction of artificial genes for expression in rice (Figure 1B). As the control, the vector plasmid pBI121 was used.

### Rice transformation

Transformation of rice, *Oryza sativa* L. cv. Nipponbare, was performed by the *Agrobacterium* method (Hiei et al. 1994). Transformant calli were cultured in N6D medium (Chu et al. 1975). The resultant calli, which grew on the medium containing hygromycin, were selected by PCR amplification of the *phaB* gene using their genomic DNA. The regenerated plants were grown in a greenhouse. Crossing of rice plants was performed using conventional procedures, and the obtained progeny F1 seeds were germinated on the agar plates containing the N6D medium.

### Determination of PHB production

Preparation and quantification of PHB in the rice transformant cells was performed according to the method described previously (Matsumoto et al. 2011). The fraction containing polyesters was extracted with chloroform from rice tissues that were harvested, lyophilized and ground using a mortar and pestle. The extracted polymer was hydrolyzed and modified to ethyl esters by an ethanolysis reaction. Ethyl 3-hydroxybutanoate generated by this reaction was measured using gas chromatography-mass spectrometry (GC-MS). A 1 µl aliquot of the sample solution was subjected to GC-MS on a GC-MS-2010 Plus system (Shimadzu, Kyoto, Japan) using the following conditions: column, Agilent HP-5MS (30 m × 0.25 mm); carrier gas, helium; injection temperature, 250°C; oven temperature, 45°C at *t*=0, then to 117°C at 7°C min^−1^, and then to 300°C at 35°C min^−1^.

### Transcriptome profiling analysis

Microarray analysis was performed twice using Agilent 4 × 44k oligoarrays that were designed by the rice microarray project in NIAS (Nuruzzaman et al. 2014). As the experimental materials, rice transformant calli, which were transformed with the *phaC* gene, both the *phaB* and *phaC* genes, and the empty vector, were examined. Similarities between the 44k microarray results were analyzed in a hierarchical clustering. Euclidean distances between the samples were calculated with the R function “dist” in the 44k dimension space and plotted onto the dendrogram using the “hclust” function with the “ward.D2” method.

Gene Ontology (GO)-terms assigned to the genes were taken as those released by the Rice Annotation Project (RAP) on 2022-09-01 (https://www.rapdb.dna.affrc.go.jp). The relationships between GO terms were calculated with co-occurrence analysis. A set of Perl and R scripts to implement the following method is available in GitHub (https://github.com/kusano-kyotouniv). The co-occurrence score of pairs of GO terms was defined by counting genes with pairs of GO terms. The top three co-occurrence targets were selected for each GO term, and the resulting biograph matrix was applied to the R library “igraph” to draw a network graph using the layout option “layout.fruchterman.reingold”. To improve the readability of the map for the “Biological Processes” category of GO terms, GO terms consisting of only one co-occurrence target were excluded from the regular map and drawn on another network graph. We assigned vertex colors using a red–white–blue color scale to indicate the upregulation and downregulation of GO term expression scores, respectively. The expression score of GO-terms was calculated using the following procedure.

The expression level of the gene was obtained by averaging multiple duplicated spot intensities of a gene. The values of four samples of the cells containing *phaC* expression were calculated relative to those of the control samples to obtain the gene expression ratios. The means of these four values were taken as the fold change in gene expression. An expression score of the GO terms was obtained as the means of the fold change in the expression of genes belonging to the appropriate GO terms. When each gene belonged to multiple GO terms, this operation was performed for each one. Changes in gene expression were visualized using a scatter plot and a volcano plot. The expression of genes belonging to the representative GO terms is highlighted on scatter plots drawn by RNAseqViewer, whose source code is provided in GitHub.

## Results

### Morphology of the rice transformants containing the genes involved in PHB synthesis

A set of genes involved in bacterial PHB biosynthesis, *phaA*, *phaB*, and *phaC*, were introduced into the vector in which these genes were set to be driven by the CaMV *35S* promoter (named *35S*–*ABC*). In addition, we constructed alternative sets including *phaC* that was driven by the *RPP17* promoter and the *SPK* promoter (named *RPP17*–*ABC* and *SPK*–*ABC*), respectively. To determine the effect of endogenous β-ketothiolase activity in rice, we also constructed a set of PHB synthesis genes consisting of *phaB* and *phaC* driven by the *35S* and *SPK* promoters (named *35S*–*BC* and *SPK*–*BC*) (Figure 1B).

**Figure 2:**
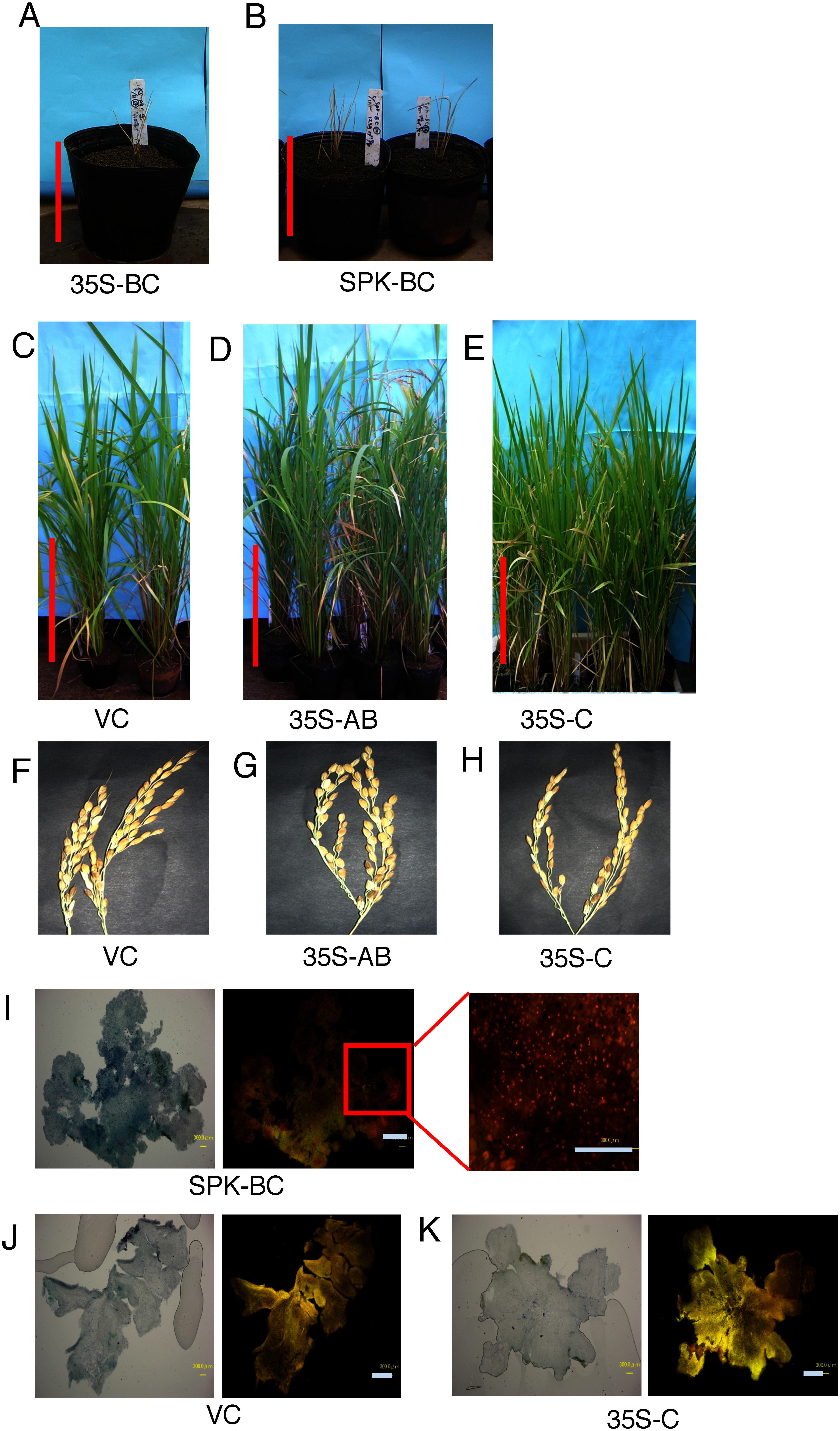
Figure 2. Features of transformant rice plants. (A–B) Morphology of the transformant plants containing *35S*–*PhaBC* (A) and *SPK*–*PhaBC* (B) two weeks after regeneration. (C–E) Features of 2-month-old *VC* (C), *35S*–*PhaAB* (D), and *35S*–*PhaC* (E) transformants. Bar is 20 cm or 50 cm. (F–H) Panicles of *VC* (F), *35S*–*PhaAB* (G), and *35S*–*PhaC* (H) transformants. (I) Detection of the accumulated PHB in the *SPK*–*PhaBC* transformant callus. The intercellular localization of PHB was determined by the Nile blue staining according to Ostle and Holt (1982). Left panel: visible image, center panel: fluorescence image, right panel: enlarged view. Bars are 1 mm (left and center) and 0.3 mm (right). (J–K) Nile blue staining of *VC* and *35S–PhaC* transformant callus. Left panel: visible image, right panel: fluorescence image. Bar is 1 mm.

These constructs were introduced into rice calli. However, very few transformants showing hygromycin resistance were obtained. In many cases, these calli soon died on regeneration medium before regeneration. The ratio of generation of the transformants was very low when *35S*–*ABC*, *RPP17*–*ABC* or *SPK*–*ABC* were introduced, whereas a large number of transformants were obtained when pBI121 was introduced (Table 1). The regenerated transformants showed a severe reduction of their growth on the regeneration medium, and many of them never grew up to a size large enough to transplant them into culture pots (Table 1). In total, we obtained 0 (0%), 2 (0.65%) and 1 (0.78%) transformants containing *35S*–*ABC*, *RPP17*–*ABC* and *SPK*–*ABC*, respectively. i.e., very few transformant plants were obtained. These transformants died before reaching reproductive growth, and no progeny were generated. *SPK* is expressed strongly in developing seeds and weakly in other sink organs including cultured cells (Asano et al. 2002a). *RPP17* expression is detected specific in phloem tissues (Asano et al. 2002b). The growth inhibition was observed in both cases. Similar results were observed when the PHB production system consisted of *phaB* and *phaC*. The regeneration ratio of the transformants was very low, and most of them died soon before transplantation into the culture pots (Table 1). In these cases, two *35S*–*BC* and two *SPK*–*BC* transformants were obtained but they all died in the vegetative stage (Figure 2A, B). PHB granules were detected in the calli of *SPK*–*BC* transformants (Figure 2I), suggesting that *phaA* was not essential for PHB production in the transformants.

**Table 1:** Table 1. Efficiency of plant regeneration from transformant cells.

Name of transgene	No. of transformants*	No. of regenerated plants**	Efficiency (%) of regeneration***
*35S*–*ABC*	409	0 (6)	0 (1.47)
*RPP*–*ABC*	308	2 (6)	0.649 (1.95)
*SPK*–*ABC*	129	1 (3)	0.775 (2.33)
*35S*–*BC*	325	2 (5)	0.615 (1.54)
*SPK*–*BC*	527	2 (7)	0.379 (1.33)
*35S*–*AB*	33	10 (10)	30.0 (30.3)
*35S*–*C*	65	11 (11)	16.9 (16.9)
*VC*	50	4 (4)	8 (8)

The names of the transgenes are cited in Figure 1B. *VC* indicates the pBI121 vector control. *Numbers of total rice calli treated with the transgene. **Numbers of plants that have regenerated and grew to the vegetative stage. ***Efficiency of regeneration. The numbers in parentheses are the total numbers of regenerated plants, including those that died immediately after regeneration.

### Transformants containing only *phaC* and a set of *phaA* and *phaB* can survive and show efficient fertility

We created a recombinant plasmid containing *phaA* and *phaB* (*35S*–*AB*) and a plasmid containing *phaC* (*35S*–*C*) (Figure 1B). From the transformant calli containing these genes, a sufficient number of regenerated plants were obtained (Table 1). They grew normally and produced a large number of progenies similar to the plants containing the vector plasmid (*VC*) (Figure 2C–E). They produced a sufficient number of seeds, suggesting that they have sufficient fertility (Figure 2F–H). No PHB granules were detected in the transformant *35S*–*C* cells (Figure 2J, K), suggesting that no PHB production occurred in these transformants. This indicates that growth inhibition was not induced when only *phaB* or *phaC* were introduced into the rice transformants.

### Determination of PHB production in the transformants

**Figure 3:**
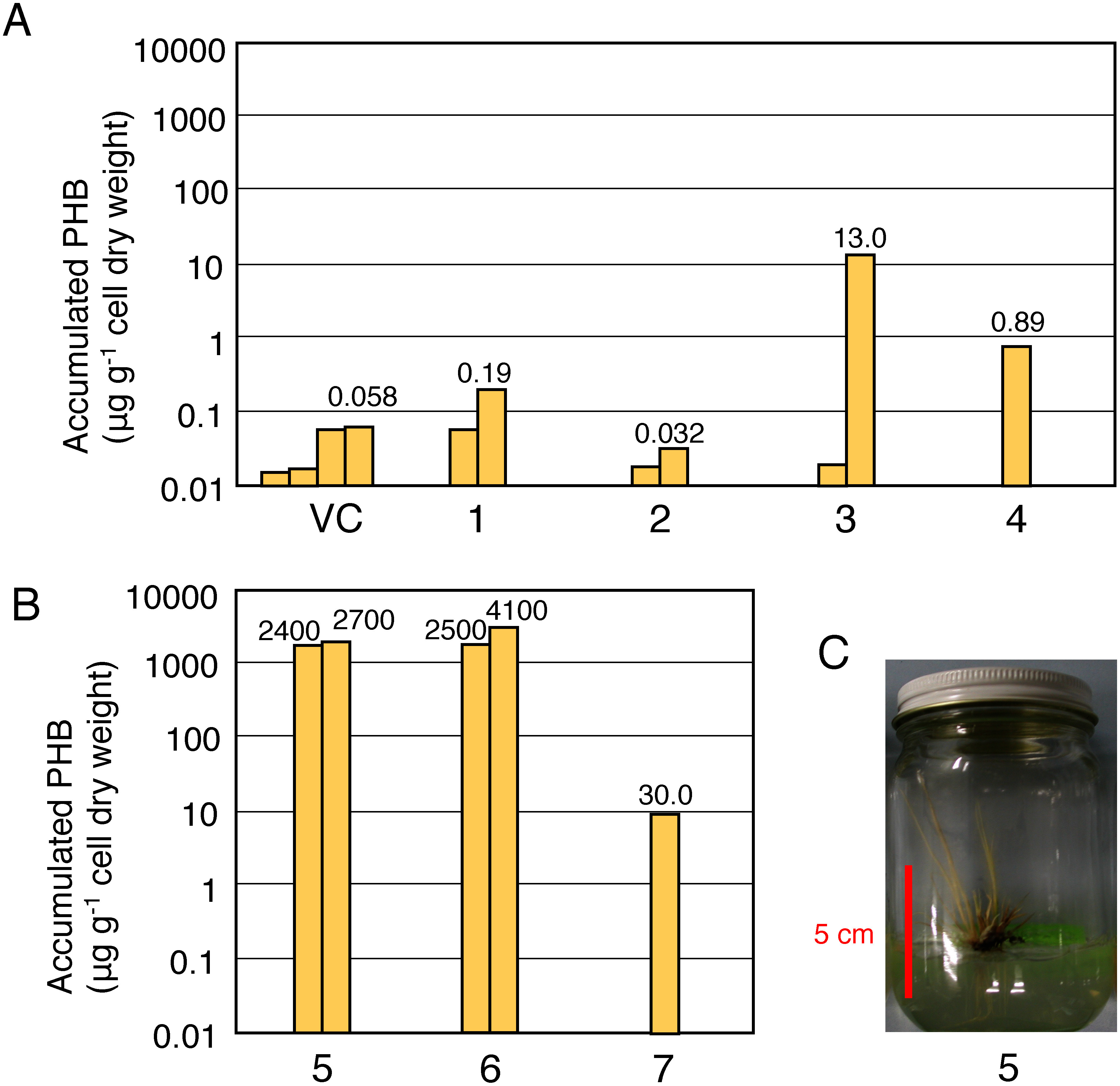
Figure 3. Accumulation of PHB in the transformants. (A) Amount of PHB accumulation in individual transformant plants. In the panel, VC and 1–4 indicate the amounts of accumulated PHB measured in *VC*, *35S*–*PhaBC*, *RPP*–*PhaBC*, *SPK*–*PhaBC*, and *SPK*–*PhaABC* transformant that were cultured 2 weeks after regeneration, respectively. (B) PHB accumulation detected in transformants that withered immediately after regeneration. In the panel, 5–7 show the amount of those in *35S*–*PhaABC* transformant, *35S*–*PhaBC* transformant, *RPP*–*PhaBC* transformant, respectively. (C) The *35S*–*PhaABC* transformant (corresponding to “5” in panel B) that died immediately after regeneration.

As mentioned above, most transformants grew little and died in the early stage of growth. However, several lines in the *RPP17*–*ABC*, *SPK*–*ABC*, *35S*–*BC* and *SPK*–*BC* transformants survived through the vegetative stage. We analyzed PHB production in the leaves of these transformants. Among them, one *SPK*–*BC* transformant line showed 13 µg g^−1^ (dcw) PHB accumulation, but the other lines accumulated small amounts of PHB that were less than 1 µg g^−1^ (dcw) (Figure 3A). PHB accumulation was also analyzed in the transformants that died soon after regeneration or no longer regenerated (Figure 3C). We found that they contained a relatively large amount of PHB accumulation compared with the living plants. More than 2,000 µg g^−1^ (dcw) and 14–6,000 µg g^−1^ (dcw) of accumulated PHB were detected in the dead transformant plants (Figure 3B) and the dead transformant callus (data not shown), respectively. This suggests that higher PHB productivity accounts for the loss of viability of the transformants.

### PHB production using F1 plants crossed between plants containing *PhaAB* and *PhaC*

**Figure 4:**
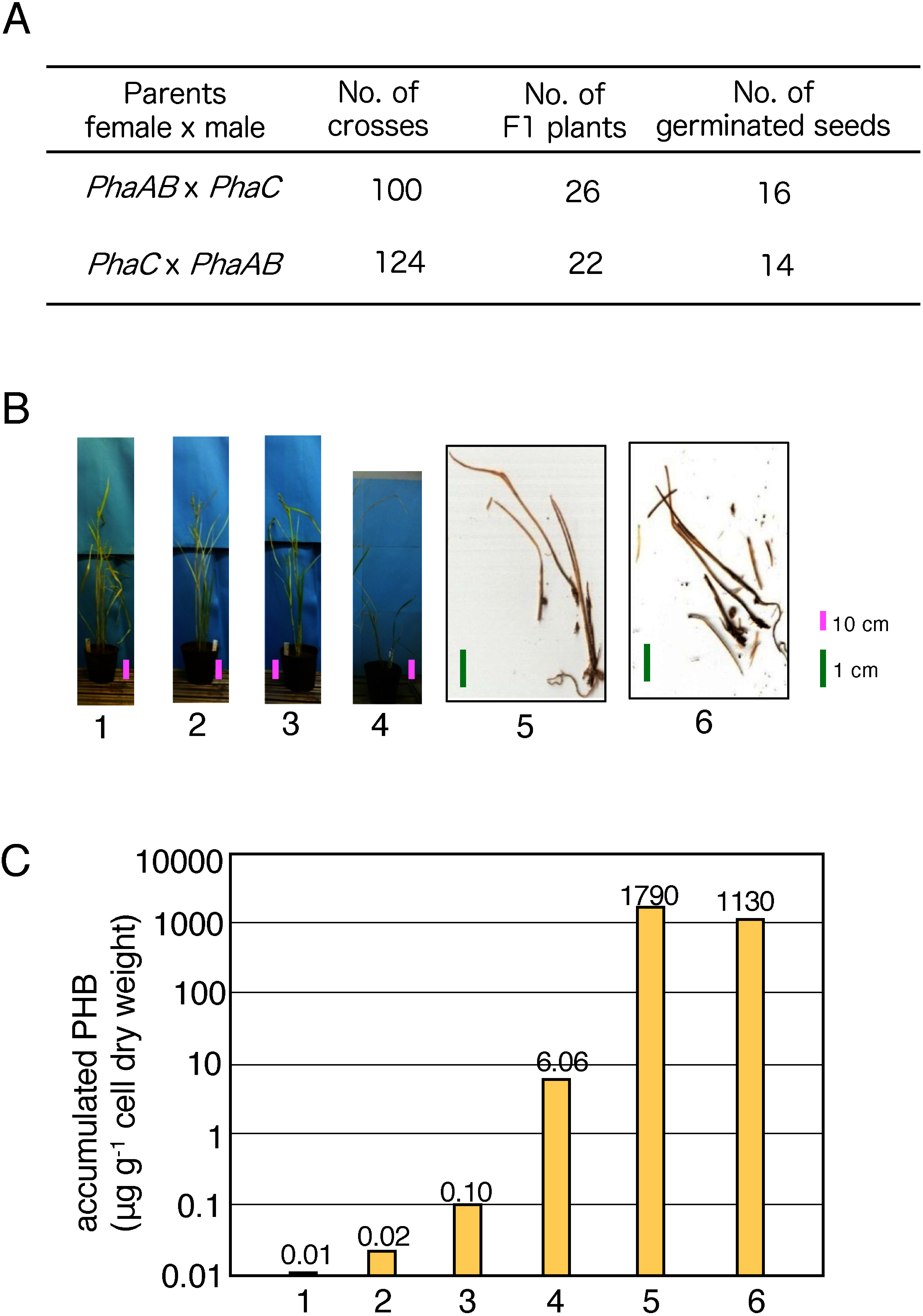
Figure 4. Production of PHB in the F1 plants crossed with *35S*–*PhaAB* and *35S*–*PhaC* transformants. (A) Results of crossing. (B) F1 phenotype. Panels 1–4 indicate features of individual 2-months-old plants. Bars are 10 cm. Panels 5 and 6 show features of plants that died soon after germination. Bars are 1 cm. (C) PHB accumulation detected in transformants is shown. Lines a to f indicate the PHB accumulation in the plants shown in Panel B.

The *35S*–*AB* transformant line was crossed with the *35S*–*C* transformant line. By this process, a set of *phaA*, *phaB* and *phaC* genes was gathered in the progenies, and PHB production was expected in the F1 plants. We obtained 48 F1 seeds in total. Among them, 30 seeds germinated (Figure 4A). The seedlings of these F1 plants contained these three genes. A large proportion of F1 plants soon died in the early growth stage due to strong growth inhibition. Among them, four F1 plants survived for more than one month and formed several leaves, although growth retardation was observed. These plants showed accumulation of PHB in amounts ranging from 0.01 µg g^−1^ dcw to 6.06 µg g^−1^ dcw in leaves (Figure 4B, C). F1 plants that stopped growing immediately after germination accumulated larger amounts of PHB compared with grown plants. The amounts of PHB accumulation in two representative dead plantlets were 1,790 µg g^−1^ dcw and 1,130 µg g^−1^ dcw (samples ‘5’ and ‘6’ in Figure 4B, C). These results show that F1 plants containing a set of *PhaA*, *PhaB* and *PhaC* allowed the production of PHB, but strong growth inhibition also occurred in relation to the PHB production ability.

### Comprehensive analysis of gene expression in the *PhaB* and *PhaC* transformants

Severe growth inhibition occurred in the transformants producing PHB. For this reason, we attempted to comprehensively analyze gene expression in the transformants harboring both *phaB* and *phaC* and those containing only *phaC*. However, no transformant plants were obtained from the calli containing both *phaB* and *phaC* because no transformants survived until regeneration. Any F1 plants from crosses between *phaB* transformants and *phaC* transformants largely fail to grow to the mature plant stage. The *35S*–*BC* transformant calli also showed strong growth retardation and could not be maintained as culture lines. The *SPK*–*BC* calli also showed delayed growth. However, in this case, its growth was less inhibited than that of the *35S*–*BC* calli and could be maintained in a callus line, although no regenerated plants were ever obtained. Therefore, we used *SPK*–*BC* callus (named the *PhaBC* line) for this analysis and compared the gene expression profiles to those of *35S*–*C* (named the *PhaC* line) and the vector control (*VC*).

**Figure 5:**
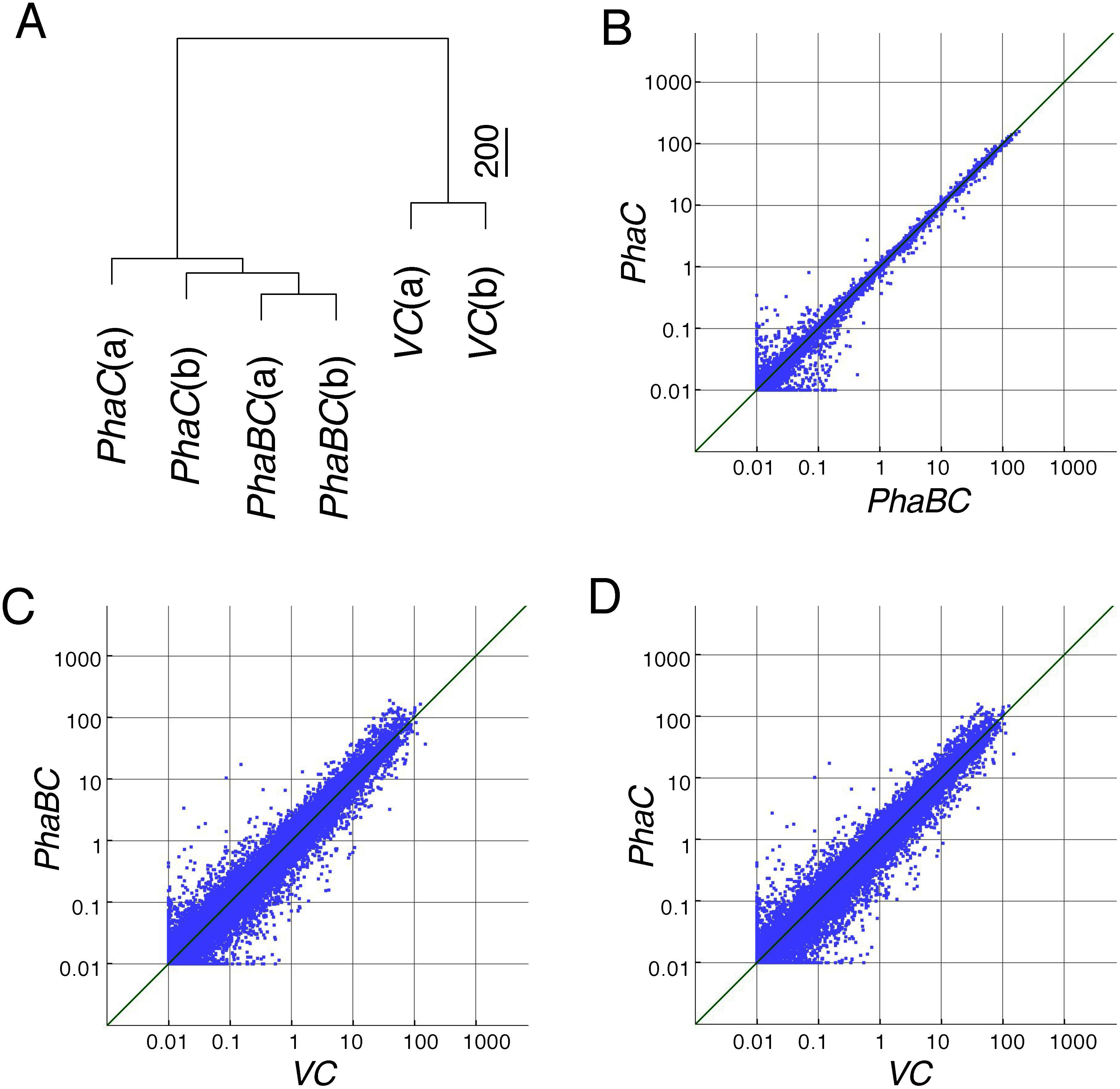
Figure 5. Microarray analysis of rice transformant calli harboring the genes involved in PHB synthesis. (A) Hierarchical clustering of microarray results. Euclidean distances between the samples were arranged into a dendrogram of hierarchical clustering in the Ward method. Scale bar indicates calculated distance at the unit of microarray intensity. Microarray analysis was performed using two independently established callus lines, which are shown by (a) and (b) following the names of the lines. (B–D) Scatter plots of the microarray data. The data from the microarray were graphed on a scatter plot to visualize variations (or reproducibility) between arrays on the representative *PhaBC* and *PhaC* lines (B), *VC* and *PhaBC* lines (C), and *VC* and *PhaC* lines (D). The values on the X and Y axes of the scatter plot indicate the means of normalized signal values for the two transformant lines examined, respectively. The green line shows the fold change.

We performed microarray analysis on two lines each of the *PhaBC*, *PhaC* and *VC* lines and confirmed the reproducibility of the results. Both the *PhaBC* and *PhaC* lines contained many genes whose expression levels changed greatly compared to those of *VC*. Surprisingly, many of these genes were commonly included in both lines. The hierarchical clustering analysis uncovered very similar patterns of expression profiles between the *PhaBC* lines and the *PhaC* lines compared to those of the *VC* lines (Figure 5A). This observation indicated that the overall expression profile in the *PhaC* lines was very similar to that in the *PhaBC* lines. Scatter plots of these microarray data also showed strong similarity between the expression profiles of the *PhaBC* and *PhaC* lines (Figure 5B). These results suggest that most of the alterations in the expressions of these genes were attributed to the expression of *phaC* in these lines.

These results also revealed that gene expression in the *PhaC* lines was greatly disturbed and disorganized although no obvious PHB production had been achieved. Therefore, we attempted to discover common effect(s) on the gene expression profiles caused by the transgenes in the two lines (*PhaC* and *PhaBC*). To meet this goal, we considered the doublet microarray data for the two lines as the results of a single dataset and applied them to statistical analysis.

### Introduction of the *phaC* gene may induce the expression of stress response genes

To obtain an overview of the physiological responses in the *phaC* expression lines, we mapped the expression scores of GO terms on the co-occurrence network of annotation assignments in the Rice Annotation Project. This analysis indicated high expression levels of genes containing several GO terms, such as lipid transport, response to oxidative stress, lipid metabolic process, and fatty acid biosynthetic process, in the *PhaC* and *PhaBC* lines (Supplementary Table S1). There were some local networks consisting of genes containing GO terms that showed up- or downregulation of gene expression (Supplementary Figures S1–S5).

GO term analysis revealed several clusters with expression up- (>2) or downregulated (<0.5) by *phaC* expression. We focused on two GO terms related to the stress response. Notably, 58 genes contained the GO term of “Response to stress”. Among them, many genes, which were annotated as peroxidases, showed high upregulation of gene expression (Supplementary Figure S6A–C). In addition, there were 145 genes that contained the GO term “response to oxidative process”, and among them, the 12 most prominently upregulated genes were annotated as “peroxidase”, “dehydrin” or “heat shock protein” (Supplementary Figure S6D–F). This finding suggests that there is some relationship between growth inhibition induced by PHB synthesis and alteration of gene expression involved in the stress responses.

Up- and downregulation of gene expression was also found in other categories for biological functions such as starch synthesis, microtubule regulation, repair process of nucleotides, and biosynthesis of cell wall components. In the networks of the “Biological Process” category, many genes with GO terms in the clusters of tRNA aminoacylation processes, including genes involved in protein translation, showed downregulation in the *phaC* expression lines (Supplementary Figure S1). Of these genes, 53 genes showed downregulated expression (Supplementary Figure S7A, B). In the networks of the “Cellular Component” category, many genes contained various GO terms in the cluster composed of photosystem-related genes, which were upregulated in the *phaC* expression lines (Supplementary Figure S4). Among them, 38 genes showed upregulated expression (Supplementary Figure S7C, D). These results suggest that *phaC* expression affects a wide range of physiological functions. However, this analysis yielded no clear evidence accounting for the growth inhibition observed in PHB-producing plants.

## Discussion

In this study, we first attempted to develop PHB-producing rice transformants by taking advantage of their high biomass production capacity. However, contrary to our initial expectations, very few number of transformants were generated from the cultured rice cells transformed with the genes for PHB biosynthesis (Table 1). Furthermore, the amount of PHB produced in these transformants was very low. The amount of PHB production was quite low in these transformants, whereas relatively greater PHB accumulation was detected in the plants that withered immediately after regeneration (Figure 2, 3). This indicates that it is very difficult to produce PHB in cultured rice cells.

*SPK* is known to be strongly expressed in immature seeds but weakly expressed in other organs (Asano et al. 2002a). RPP17 is specifically expressed in phloem tissues (Asano et al. 2002b). Therefore, it was expected to perform low level expression in the cultured cells when using these promoters. However, such growth inhibition was observed to occur even when the genes involved in PHB production were driven by relatively weak promoters, such as *SPK* and *RPP17* promoters, which were expected to reduce the negative effects on growth. This result strongly implies that production of PHB unexpectedly causes severe growth inhibition in transgenic rice.

The *PhaBC* transformants, which died immediately after regeneration, showed a higher PHB accumulation than surviving plants, but the total amount of PHB produced was quite small in both cases. We presumed that even a small amount of PHB produced would induce strong growth inhibition during regeneration. Therefore, we expected to improve PHB productivity if the growth inhibition that occurred immediately after regeneration could be avoided. *PhaAB* and *PhaC* transformants, which do not produce PHB, were efficiently obtained without significant growth inhibition. We attempted to use them to establish a PHB-producing F1 plant by crossing.

Crossing *35S*–*AB* and *35S*–*C* transformants resulted in F1 seeds in which all three genes were included. These F1 seeds germinated, and PHB production was observed in the F1 plants. However, most of the F1 plants also showed strong growth inhibition and did not grow to the reproductive stage (Figure 4). This result indicates difficulty in PHB production even in F1 plants in which all of the genes involved in PHB production were introduced. This strongly suggests that PHB production in plants causes severe damage that makes survival difficult for these plants. Therefore, it is necessary to establish a new procedure that may avoid growth inhibition due to PHB production in the transformants. Accordingly, we aimed to elucidate the mechanism that caused a severe growth inhibition by observing this phenomenon in detail. First, we investigated whether the growth inhibition was due to PHB production or the expression of a transgene involved in PHB synthesis.

When the promoter for the *phaC* gene was changed from *35S* to *SPK*, we obtained a small number of regenerated plants from the transformants harboring the *SPK*-driven *phaB* and *phaC* genes, whereas none were obtained from those containing the *35S* promoter-driven genes (Figure 2). This implies that sufficient amounts of PHB may be produced in mature plants if the *phaC* gene is not expressed during the young plant stage.

Analysis of the strong growth inhibition that occurred in the PHB-producing transformants may provide insight into this mechanism. We observed that transformants that did not produce PHB, such as the *PhaAB* and *PhaC* transformants, generated their progeny seeds without growth inhibition (Figure 2). This suggests that PHB production is a major threat to plant survival.

To explore the mechanisms behind this phenomenon, a comprehensive microarray analysis was performed on gene expression of the *PhaBC*, *PhaC*, and *VC* transformants. In this analysis, we attempted to use the *PhaBC* transformant that accumulated PHA, but the *35S*–*PhaBC* showed severe growth inhibition and we never enabled to obtain a sufficient amount of RNA samples. Therefore, we used another PHB-producing transformant, *SPK*–*PhaBC*, from which RNA sample could be prepared. *SPK* is expected to be weakly expressed both in cultured cells and leaves as described above (Asano et al. 2002a). Therefore, it was expected to perform low level protein production. The transformant containing *phaC* gene driven by the *SPK* promoter had a relatively small degree of growth inhibition, and could be maintained as cultured cells. The transcriptome analysis was performed using this and the *PhaC* and *VC* transformant that did not show growth inhibition. This analysis revealed many changes in gene expression in both the *PhaBC* and *PhaC* transformants.

Microarray analysis exhibited that the overall gene-expression patterns of the *PhaBC* and *PhaC* transformants were very similar to each other (Figure 5). Some genes appeared to differ in expression level between *PhaBC* and *PhaC* transformants, but the ratio of genes that were detected only in *PhaBC* was less than 5% in genes showing different expression patterns to that of *VC*. This indicates that most of the changes in gene expression were mainly caused by *phaC* gene expression. However, this result is inconsistent with the observation that strong growth inhibition was induced by PHB production. 3HB-CoA, a substrate of PHB, possibly exists in plants because plant has a metabolic pathway involved in generation of 3HB-CoA from acetyl-CoA, and therefore, it is not excluded that a small amount of PHB could be produced in the transformant in a PhaC transformant. It is unclear whether this has any adverse effects. We presumed that additional expression of PhaC caused some unknown metabolic perturbation, which is not lethal in the PhaC transformant. It is also presumed that expression of both PhaB and PhaC induced a lethal damage to the PhaBC transformants harboring the PHB biosynthetic pathway.

Although many attempts have been performed to efficiently produce PHB in plants, few studies have focused on the physiological characteristics for PHB production. To our knowledge, there has been no studies on the PhaC expression that affects to other genes and any traits that it confers. It is noteworthy that many of the upregulated genes included the GO terms of stress-response genes, such as peroxidases, dehydrins and heat shock proteins (Supplementary Figure S6). Peroxidases, dehydrins and heat shock proteins are well known to function under stress conditions such as cold, drought and osmotic stresses (e.g., Pandey et al. 2017; Puhakainen et al. 2004; Rorat 2006; Wang et al. 2004). It was assumed that the expression of these genes increased in response to PHB production. Programmed cell death has been reported to be induced by a specific type of stress (Burke et al. 2020; Cimini et al. 2019; Petrov et al. 2015). The negative effects of PHB accumulation on plant growth may be related to stress responses.

Additionally, significant changes in gene expression were found for many genes involved in certain biological processes and cellular components (Supplementary Figures S6 and S7). These findings suggest that PhaC activity caused some kind of metabolic disturbance, leading to the disruption of intercellular physiological functions. These results strongly suggest that the activity of introduced *phaC* had a great effect on the transformants. These observations imply that the gene expression profile is perturbed by the transgene, even if the effect has not reached a phenotypically observable level. Such metabolic disturbance was considered to be enhanced in the PHB-producing transformants, resulting in lethal consequences.

The strength of growth inhibition due to PHB production largely varies depending on the plant species. Several reports have shown PHB production in plant organelles. It has been reported that PHB in a maximum of 1.88% of dry weight accumulated in the mitochondria of sugarcane leaves without obvious deleterious effects (Petrasovits et al. 2007). It is known that transformants producing PHB in the cytosols show strong growth inhibition (e.g., Bohmert et al. 2000). However, production of 3.72% of the dry weight of PHB has been achieved in the leaves of switchgrass (Somleva et al. 2008). It has been reported that growth inhibition occurred in *Arabidopsis* harboring the genes involved in PHB production, but cell viability was increased by supplementing the culture medium with sucrose (Yoshizumi et al. 2017). Investigating the metabolic disturbances caused by PHB production may provide some hints for selecting plant species suitable for PHB production. If the genes driven by an inducible or a specific promoter were used as the genes involved in PHB production, this could be achieved. Our findings are expected to be useful for selecting plant hosts that can tolerate the expression of PHB biosynthetic genes.

## Description of Supplementary Files

Supplementary Table S1. Gene Ontology (GO) enrichment analysis.

Supplementary Figure S1. Co-occurrence network map of GO terms in the “Biological process” category.

Supplementary Figure S2. Co-occurrence pair map of GO terms in the “Biological process” category.

Supplementary Figure S3. Co-occurrence map of orphan GO terms in the “Biological process” category.

Supplementary Figure S4. Co-occurrence map of GO terms in the “Cellular components” category.

Supplementary Figure S5. Co-occurrence map of GO terms in the “Cellular components” category.

Supplementary Figure S6. Scatter plots of the genes annotated with the GO terms response to stress and response to oxidative stress.

Supplementary Figure S7. Scatter plots of the genes annotated with the GO terms of tRNA aminoacylation and photosystem-related cluster.

## Notes

dcw: dry cell weight
PCR: polymerase chain reaction

## References

[RAcharjee2023] Acharjee SA, Bharali P, Gogoi B, Sorhie V, Walling B, Alemtoshi (2023) PHA-based bioplastoc: A potential alternative to address microplatic pollution. *Water Air Soil Pollut* 234: 21 36593989 10.1007/s11270-022-06029-2 PMC9797907

[RArai2004] Arai Y, Shikanai T, Doi Y, Yoshida S, Yamaguchi I, Nakashita H (2004) Production of polyhydroxybutyrate by polycistronic expression of bacterial genes in tobacco plastid. *Plant Cell Physiol* 45: 1176–1184 15509840 10.1093/pcp/pch139

[RAsano2002a] Asano T, Kunieda N, Omura Y, Ibe H, Kawasaki T, Takano M, Sato M, Furuhashi H, Mujin T, Takaiwa F, et al. (2002a) Rice SPK, a calmodulin-like domain protein kinase, is required for storage product accumulation during seed development: Phosphorylation of sucrose synthase is a possible factor. *Plant Cell* 14: 619–628 11910009 10.1105/tpc.010454 PMC150584

[RAsano2002b] Asano T, Kusano H, Okuda T, Kubo N, Shimada H, Kadowaki K (2002b) *Rpp16* and *Rpp17*, from a common origin, have different protein characteristics but both genes are predominantly expressed in rice phloem tissues. *Plant Cell Physiol* 43: 668–674 12091721 10.1093/pcp/pcf083

[RAtiwesh2021] Atiwesh G, Mikhael A, Parrish CC, Banoub J, Le T-AT (2021) Environmental impact of bioplastic use: A review. *Heliyon* 7: e07918 34522811 10.1016/j.heliyon.2021.e07918 PMC8424513

[RBohmert2000] Bohmert K, Balbo I, Kopka J, Mittendorf V, Nawrath C, Poirier Y, Tischendorf G, Trethewey RN, Willmitzer L (2000) Transgenic *Arabidopsis* plants can accumulate polyhydroxybutyrate to up to 4% of their fresh weight. *Planta* 211: 841–845 11144269 10.1007/s004250000350

[RBurke2020] Burke R, Schwarze J, Sherwood OL, Jnaid Y, McCabe PF, Kacprzyk J (2020) Stressed to death: The role of transcription factors in plant programmed cell death induced by abiotic and biotic stimuli. *Front Plant Sci* 11: 1235 32903426 10.3389/fpls.2020.01235 PMC7434935

[RChu1975] Chu CC, Wang CC, Sun CS, Hsu C, Yin KC, Chu CY, Bi FY, Chu C, Wang NC, Sun CP, et al. (1975) Establishment of an efficient medium for anther culture of rice through comparative experiments on the nitrogen. *Sci Sin* 18: 659–668

[RClimini2019] Cimini S, Gualtieri C, Macovei A, Balestrazzi A, De Gara L, Locato V (2019) Redox balance-DDR-miRNA triangle: Relevance in genome stability and stress responses in plants. *Front Plant Sci* 10: 989 31428113 10.3389/fpls.2019.00989 PMC6688120

[RHiei1994] Hiei Y, Ohta S, Komari T, Kumashiro T (1994) Efficient transformation of rice (*Oryza sativa* L.) mediated by *Agrobacterium* and sequence analysis of the boundaries of the T-DNA. *Plant J* 6: 271–282 7920717 10.1046/j.1365-313x.1994.6020271.x

[RHoumiel1999] Houmiel KL, Slater S, Broyles D, Casagrande L, Colburn S, Gonzalez K, Mitsky TA, Reiser SE, Shah D, Taylor NB, et al. (1999) Poly(β-hydroxybutyrate) production in oilseed leukoplasts of *Brassica napus.* *Planta* 209: 547–550 10550638 10.1007/s004250050760

[RMatsumoto2011] Matsumoto K, Morimoto K, Gohda A, Shimada H, Taguchi S (2011) Improved polyhydroxybutyrate (PHB) production in transgenic tobacco by enhancing translation efficiency of bacterial PHB biosynthetic genes. *J Biosci Bioeng* 111: 485–488 21185778 10.1016/j.jbiosc.2010.11.020

[RMatsumoto2009] Matsumoto K, Murata T, Nagao R, Nomura CT, Arai S, Arai Y, Takase K, Nakashita H, Taguchi S, Shimada H (2009) Production of short-chain-length/medium-chain-length polyhydroxyalkanoate (PHA) copolymer in the plastid of *Arabidopsis thaliana* using an engineered 3-keto-acyl carrier protein synthase III. *Biomacromolecules* 10: 686–690 19265441 10.1021/bm8013878

[RMoshood2022] Moshood TD, Nawanir G, Mahmud F, Mohamad F, Ahmad MH, AbdulGhani A (2022) Sustainability of biodegradable plastics: New problem or solution to solve the global plastic pollution? *Curr Res Green Sustain Chem* 5: 100273

[RMott2000] Mott IEC, Hughes A, Dunnill P (2000) An ultra scale-down process study for the production of polyhydroxybutyrate from transgenic rapeseed. *Bioprocess Biosyst Eng* 22: 451–459

[RNuruzzaman2014] Nuruzzaman M, Sharoni AM, Satoh K, Kumar A, Leung H, Kikuchi S (2014) Comparative transcriptome profiles of the *WRKY* gene family under control, hormone-treated, and drought conditions in near-isogenic rice lines reveal differential, tissue specific gene activation. *J Plant Physiol* 171: 2–13 24189206 10.1016/j.jplph.2013.09.010

[ROstle1982] Ostle AG, Holt JG (1982) Nile blue A as a fluorescent stain for poly-β-hydroxybutyrate. *Appl Environ Microbiol* 44: 238–241 6181737 10.1128/aem.44.1.238-241.1982 PMC241995

[RPandey2017] Pandey S, Fartyal D, Agarwal A, Shukla T, James D, Kaul T, Negi Y, Arora S, Reddy M (2017) Abiotic stress tolerance in plants: Myriad roles of ascorbate peroxidase. *Front Plant Sci* 8: 581 28473838 10.3389/fpls.2017.00581 PMC5397514

[RPeoples1989] Peoples OP, Sinskey AJ (1989) Poly-β-hydroxybutyrate biosynthesis in *Alcaligenes eutrophus* H16. *J Biol Chem* 264: 15293–15297 2670935

[RPetrasovits2007] Petrasovits LA, Purnell MP, Nielsen LK, Brumbley SM (2007) Production of polyhydroxybutyrate in sugarcane. *Plant Biotechnol J* 5: 162–172 17207265 10.1111/j.1467-7652.2006.00229.x

[RPetrov2015] Petrov V, Hille J, Mueller-Roeber B, Gechev TS (2015) ROS-mediated abiotic stress-induced programmed cell death in plants. *Front Plant Sci* 6: 69 25741354 10.3389/fpls.2015.00069 PMC4332301

[RPoirier1992] Poirier Y, Dennis D, Klomparens K, Somerville C (1992) Polyhydroxybutyrate, a biodegradable thermoplastic, produced in transgenic plants. *Science* 256: 520–523 17787950 10.1126/science.256.5056.520

[RPuhakainen2004] Puhakainen T, Hess MW, Mäkelä P, Svensson J, Heino P, Palva ET (2004) Overexpression of multiple dehydrin genes enhances tolerance to freezing stress in Arabidopsis. *Plant Mol Biol* 54: 743–753 15356392 10.1023/B:PLAN.0000040903.66496.a4

[RPurnell2007] Purnell MP, Petrasovits LA, Nielsen LK, Brumbley SM (2007) Spatio-temporal characterization of polyhydroxybutyrate accumulation in sugarcane. *Plant Biotechnol J* 5: 173–184 17207266 10.1111/j.1467-7652.2006.00230.x

[RRomano2005] Romano A, van der Plas LHW, Witholt B, Eggink G, Mooibroek H (2005) Expression of poly-3-(R)-hydroxyalkanoate (PHA) polymerase and acyl-CoA-transacylase in plastids of transgenic potato leads to the synthesis of a hydrophobic polymer, presumably medium-chain-length PHAs. *Planta* 220: 455–464 15351883 10.1007/s00425-004-1349-8

[RRomano2003] Romano A, Vreugdenhil D, Jamar D, van der Plas LHW, De Roo G, Witholt B, Eggink G, Mooibroek H (2003) Evidence of medium-chain-length polyhydroxyoctanoate accumulation in transgenic potato lines expressing the *Pseudomonas oleovorans* Pha-C1 polymerase in the cytoplasm. *Biochem Eng J* 16: 135–143

[RRorat2006] Rorat T (2006) Plant dehydrins: Tissue location, structure and function. *Cell Mol Biol Lett* 11: 536–556 16983453 10.2478/s11658-006-0044-0 PMC6275985

[RSlater1999] Slater S, Mitsky TA, Houmiel KL, Hao M, Reiser SE, Taylor NB, Tran M, Valentin HE, Rodriguez DJ, Stone DA, et al. (1999) Metabolic engineering of *Arabidopsis* and *Brassica* for poly(3-hydroxybutyrate-*co*-3-hydroxyvalerate) copolymer production. *Nat Biotechnol* 17: 1011–1016 10504704 10.1038/13711

[RSomleva2013] Somleva MN, Peoples OP, Snell KD (2013) PHA bioplastics, biochemicals, and energy from crops. *Plant Biotechnol J* 11: 233–252 23294864 10.1111/pbi.12039

[RSomleva2008] Somleva MN, Snell KD, Beaulieu JJ, Peoples OP, Garrison BR, Patterson NA (2008) Production of polyhydroxybutyrate in switchgrass, a value-added co-product in an important lignocellulosic biomass crop. *Plant Biotechnol J* 6: 663–678 18498309 10.1111/j.1467-7652.2008.00350.x

[RTang2022] Tang HJ, Neoh SZ, Sudesh K (2022) A review on poly(3-hydroxybutyrate-*co*-3-hydroxyhexanoate) [P(3HB-*co*-3HHx)] and genetic modifications that affect it production. *Front Bioeng Biotechnol* 10: 1057067 36545679 10.3389/fbioe.2022.1057067 PMC9760699

[Rvan2008] van Beilen JB, Poirier Y (2008) Production of renewable polymers from crop plants. *Plant J* 54: 684–701 18476872 10.1111/j.1365-313X.2008.03431.x

[RWang2004] Wang W, Vinocur B, Shoseyov O, Altman A (2004) Role of plant heat-shock proteins and molecular chaperones in the abiotic stress. *Trends Plant Sci* 9: 244–252 15130550 10.1016/j.tplants.2004.03.006

[RYoshizumi2017] Yoshizumi T, Yamada M, Higuchi-Takeuchi M, Matsumoto K, Taguchi S, Matsui M, Numata K (2017) Sucrose supplementation suppressed the growth inhibition in polyhydroxyalkanoate-producing plants. *Plant Biotechnol (Tokyo)* 34: 39–43 31275006 10.5511/plantbiotechnology.16.1121a PMC6543704

