# Supplementary material for: Insights into widespread disturbance in gene expression and severe growth inhibition observed in transgenic rice producing polyhydroxybutyrate
Source: Plant Biotechnol (Tokyo). 2025 Mar 25;42(1):41–9. doi: 10.5511/plantbiotechnology.24.1107a (PMC12622900; doi:10.5511/plantbiotechnology.24.1107a)
Supplement: Supplementary Data [file plantbiotechnology-42-1-24.1107a-s001.pdf]

Supplementary Table S1. Gene Ontology (GO) enrichment analysis. GO terms were sorted and highlighted by the weighted enrichment index.

GO ID	GO term	genes	enrichment	log10(enrich)	log(enrich) x genes	SpkBC1	SpkBC2	35S-C1	35S-C2	SpkBC	35S-C
GO:0006869	lipid transport	79	1.75321213	0.24383447	19.2629229	1.84265359	1.71150962	1.59621416	1.8768245	1.77587144	1.73084195
GO:0006979	response to oxidative stress	145	1.1793493	0.07164245	10.3881559	1.27392831	1.1593437	1.05763676	1.23844319	1.21528628	1.14447501
GO:0006629	lipid metabolic process	219	1.09947269	0.04118445	9.01939371	1.16397087	1.08595516	1.0172803	1.1364312	1.12428651	1.07520652
GO:0006633	fatty acid biosynthetic process	66	1.25255253	0.09779595	6.45453257	1.32505452	1.22598539	1.13277381	1.33758549	1.27455776	1.23092722
GO:0009765	photosynthesis%2C light harvesting	11	3.49217118	0.54309552	5.97405076	3.88059071	3.32844482	3.06606454	3.75544669	3.59392989	3.39329367
GO:0007018	microtubule-based movement	52	1.29264566	0.11147949	5.79693361	1.36906611	1.25623844	1.1703485	1.3870952	1.31143947	1.27412118
GO:0008610	lipid biosynthetic process	23	1.54424858	0.18871721	4.34049585	1.60410983	1.51882831	1.41776479	1.64634888	1.56088674	1.52778777
GO:0071577	zinc ion transmembrane transport	10	2.63790478	0.42125911	4.21259114	2.83568604	2.60075887	2.33359407	2.81353882	2.71568327	2.5623539
GO:0030001	metal ion transport	76	1.12599675	0.05153714	3.91682255	1.20678549	1.1038955	1.00769235	1.19746423	1.15419456	1.09848784
GO:0000160	two-component signal transduction system (phosph	39	1.25298602	0.09794622	3.81990277	1.35016446	1.19849563	1.15230719	1.32188447	1.27207162	1.23418677
GO:0006334	nucleosome assembly	53	1.18035222	0.07201162	3.81661601	1.25276172	1.13390779	1.08032755	1.2648669	1.19185413	1.16896132
GO:0019953	sexual reproduction	16	1.7155575	0.23440528	3.75048445	1.88878621	1.70659367	1.52235317	1.76519529	1.79538035	1.63928358
GO:0015979	photosynthesis	42	1.21550483	0.08475669	3.559781	1.30906449	1.1822199	1.08186914	1.3037452	1.24402656	1.18763702
GO:0006833	water transport	9	2.31756456	0.36503184	3.28528658	2.5352972	2.27406853	2.0984926	2.38444605	2.40113298	2.23690465
GO:0042545	cell wall modification	35	1.22553122	0.08832438	3.09135332	1.28548328	1.17175496	1.22628595	1.22124427	1.22730249	1.22376251
GO:0009664	plant-type cell wall organization	26	1.30785958	0.11656112	3.03058905	1.49033065	1.26135013	1.13309829	1.37359467	1.37106847	1.24756473
GO:0009416	response to light stimulus	22	1.35324991	0.13137801	2.89031614	1.58919236	1.26044703	1.19061413	1.40617678	1.41530661	1.2939142
GO:0030418	nicotianamine biosynthetic process	3	8.06189595	0.90643719	2.71931157	8.55189935	7.85895908	7.22961032	8.69373216	8.19811119	7.92794398
GO:0008299	isoprenoid biosynthetic process	23	1.23927789	0.0931687	2.14288011	1.32150034	1.19534572	1.11701153	1.3367689	1.25684119	1.22196002
GO:0006520	cellular amino acid metabolic process	41	1.1083534	0.04467826	1.83180854	1.20952501	1.06509231	0.98511646	1.18911361	1.13501356	1.08231945
GO:0006486	protein glycosylation	40	1.11107054	0.04574163	1.82966522	1.17396003	1.08470327	1.00840816	1.18676823	1.1284495	1.09395922
GO:0006281	DNA repair	75	1.05546255	0.02344283	1.75821213	1.11728645	1.01057179	0.97081754	1.13214485	1.06259031	1.0483826
GO:0006260	DNA replication	52	1.08058063	0.03365718	1.75017323	1.14910896	1.0448236	0.9761762	1.16331227	1.09572632	1.06564429
GO:0009690	cytokinin metabolic process	7	1.75512834	0.24430888	1.71016216	1.91154332	1.68117443	1.61104674	1.83286492	1.7926622	1.71838035
GO:0042218	1-aminocyclopropane-1-carboxylate biosynthetic pr	12	1.38470447	0.14135709	1.69628514	1.48029261	1.34443005	1.25517777	1.47176123	1.41072671	1.35916223
GO:0005978	glycogen biosynthetic process	6	1.90569504	0.2800534	1.68032042	1.99365265	1.84197038	1.70857461	2.10207173	1.91631133	1.89513756
GO:0000079	regulation of cyclin-dependent protein kinase activit	30	1.13468798	0.05487646	1.64629368	1.25556328	1.11642416	0.99532473	1.18815527	1.18395151	1.08747428
GO:0006754	ATP biosynthetic process	26	1.14278445	0.05796432	1.50707237	1.20425217	1.12814247	1.05302037	1.19217258	1.16557626	1.12043831
GO:0043086	negative regulation of catalytic activity	38	1.0937751	0.03892803	1.47926529	1.20781656	1.0391357	1.00584703	1.13372299	1.1203059	1.06787261
GO:0009790	embryo development	3	2.94261014	0.46873273	1.40619818	2.86096378	3.29855109	2.56169692	3.10147	3.07197578	2.81869228
GO:0006310	DNA recombination	15	1.22630173	0.08859734	1.32896012	1.2977803	1.237461	1.08041368	1.30336662	1.26726181	1.18666555
GO:0006270	DNA-dependent DNA replication initiation	7	1.51249763	0.1796947	1.25786292	1.8417726	1.32118559	1.42346374	1.51088903	1.55991135	1.46652506
GO:0006284	base-excision repair	20	1.15567304	0.06283498	1.25669966	1.21837925	1.1212568	1.04403772	1.25065108	1.16880966	1.14268407
GO:0009415	response to water	7	1.47331128	0.16829451	1.17806159	1.49225872	1.5323288	1.38740414	1.4851796	1.51216104	1.43545962
GO:0005975	carbohydrate metabolic process	444	1.00612723	0.0026529	1.17788959	1.06759083	0.98936499	0.92360071	1.05042726	1.02773391	0.9849748
GO:0006536	glutamate metabolic process	5	1.67701417	0.22453673	1.12268366	1.74353249	1.68126414	1.52167106	1.77320992	1.71211526	1.64263271
GO:0022900	electron transport chain	8	1.37513301	0.13834471	1.10675767	1.45802661	1.33377119	1.23953973	1.48344552	1.39451564	1.35601979
GO:0009089	lysine biosynthetic process via diaminopimelate	10	1.27747276	0.10635165	1.06351647	1.40856915	1.27952855	1.10889966	1.33255819	1.34249933	1.21559588
GO:0046274	lignin catabolic process	24	1.10207012	0.04220923	1.01302151	1.17585896	1.11758226	1.01331216	1.10779381	1.14635035	1.05950032
GO:2000123	positive regulation of stomatal complex developmen	1	10.102704	1.00443763	1.00443763	11.132972	9.38140472	9.28183302	10.7457758	10.2197317	9.98701638
GO:0042744	hydrogen peroxide catabolic process	1	9.88531913	0.99499069	0.99499069	11.10703	9.507768	8.907513	10.15149	10.2763352	9.50918131
GO:0006546	glycine catabolic process	6	1.44405941	0.15958506	0.95751036	1.50696882	1.43173548	1.32231086	1.52419181	1.46887056	1.41966735
GO:0006200	ATP catabolic process	13	1.18467523	0.07359931	0.95679102	1.25050988	1.1750717	1.09927861	1.21937689	1.2122041	1.15777154
GO:0006066	alcohol metabolic process	5	1.52968163	0.18460105	0.92300525	1.93980364	1.1851106	1.39558136	1.70660254	1.5162064	1.54327661
GO:0016310	phosphorylation	20	1.10894276	0.04490913	0.8981826	1.16834031	1.06884041	1.00029071	1.21067635	1.11748348	1.10046731
GO:0051726	regulation of cell cycle	16	1.1301635	0.05314128	0.85026042	1.21325337	1.14206505	1.00277187	1.17414198	1.17712118	1.08507905
GO:0006094	gluconeogenesis	7	1.24341705	0.09461682	0.66231772	1.28253398	1.2113541	1.11290085	1.38251803	1.24643604	1.24040537
GO:0009250	glucan biosynthetic process	5	1.34228565	0.12784495	0.63922474	1.39661753	1.29606084	1.23124987	1.45656783	1.34540005	1.33917846
GO:0042549	photosystem II stabilization	1	4.2125923	0.62454943	0.62454943	4.70355949	4.01771319	3.69118361	4.51467504	4.34713158	4.08221687
GO:0006556	S-adenosylmethionine biosynthetic process	4	1.42568635	0.15402399	0.61609596	1.51186412	1.33695296	1.37474123	1.48677796	1.42172121	1.42966254
GO:0051188	cofactor biosynthetic process	2	2.01276367	0.30379279	0.60758557	2.0715991	1.97718489	1.84818577	2.16806613	2.02384151	2.00174648
GO:0010411	xyloglucan metabolic process	3	1.59313708	0.20225315	0.60675944	1.69310896	1.64779741	1.48366741	1.55627846	1.67029954	1.51953928
GO:0007275	multicellular organismal development	13	1.11062491	0.04556741	0.59237634	1.20532642	1.10533081	0.98667629	1.15743869	1.15424626	1.0686521
GO:0042128	nitrate assimilation	2	1.97717553	0.29604523	0.59209046	2.23887128	1.87147173	1.81756807	2.00667865	2.04694511	1.90978403
GO:0006021	inositol biosynthetic process	2	1.93562199	0.28682055	0.57364109	1.51569495	4.17985482	1.36700392	1.6208373	2.51701904	1.48851972
GO:0006696	ergosterol biosynthetic process	3	1.54877247	0.18998762	0.56996286	1.66098496	1.54440008	1.381553	1.62351916	1.60163208	1.49765743
GO:0008219	cell death	11	1.12652416	0.05174051	0.56914561	1.1618072	1.18612942	1.03054116	1.13404571	1.17390532	1.0810554
GO:0007017	microtubule-based process	21	1.06382349	0.02686958	0.5642611	1.11616215	1.02909905	0.9753006	1.14328722	1.07174689	1.05595867
GO:0006527	arginine catabolic process	2	1.89073959	0.27663172	0.55326344	2.12394327	1.80176088	1.6667728	2.00359713	1.95623053	1.82744116
GO:0019464	glycine decarboxylation via glycine cleavage system	4	1.36160514	0.13405118	0.53620473	1.43909912	1.34579536	1.26605252	1.40179101	1.39166552	1.33219407
GO:0007050	cell cycle arrest	5	1.27984403	0.10715705	0.53578524	1.35599468	1.21837766	1.22236907	1.32857456	1.28534572	1.2743659
GO:0051258	protein polymerization	17	1.07386291	0.03094884	0.52613034	1.13742488	1.02849731	0.99271554	1.1451033	1.08159069	1.06619034
GO:0006542	glutamine biosynthetic process	5	1.27048994	0.10397123	0.51985616	1.35489952	1.25165913	1.14423648	1.3426907	1.30225664	1.23949815
GO:0045038	protein import into chloroplast thylakoid membrane	1	3.08210668	0.48884767	0.48884767	3.68175283	2.89075891	2.7706367	3.06016315	3.26237028	2.91180362
GO:0000902	cell morphogenesis	4	1.31935692	0.1203623	0.4814492	1.39692575	1.2289254	1.18357395	1.49126544	1.31023568	1.32854166
GO:0009082	branched chain family amino acid biosynthetic proce	9	1.12460236	0.05099899	0.45899091	1.167527	1.10701139	1.02634963	1.20581756	1.13686661	1.11247041
GO:0009813	flavonoid biosynthetic process	3	1.41710706	0.15140266	0.45420798	1.71431304	1.30311898	1.1869124	1.52095994	1.49464172	1.34359451
GO:0006950	response to stress	57	1.01851307	0.00796661	0.45409665	1.0771982	0.99381267	0.92301686	1.08907214	1.03466575	1.00261256
GO:0030042	actin filament depolymerization	3	1.39996603	0.1461175	0.43835249	1.5238664	1.34819741	1.25276979	1.49244501	1.4333432	1.36736609
GO:0009607	response to biotic stimulus	6	1.18128063	0.07235308	0.43411849	1.31857844	1.08736026	1.08874755	1.2473999	1.19740127	1.16537702
GO:0009909	regulation of flower development	1	2.69522877	0.43059563	0.43059563	2.489784	2.74305033	2.864672	2.697194	2.61335088	2.77967195
GO:0048573	photoperiodism%2C flowering	1	2.69522877	0.43059563	0.43059563	2.489784	2.74305033	2.864672	2.697194	2.61335088	2.77967195
GO:0006890	retrograde vesicle-mediated transport%2C Golgi to I	5	1.21639626	0.08507508	0.42537538	1.245951	1.14633336	1.1614186	1.31977421	1.19510468	1.23806717
GO:0008295	spermidine biosynthetic process	5	1.21370207	0.08411209	0.42056046	1.2746699	1.17191892	1.118174	1.2991033	1.22221511	1.20524833
GO:0000103	sulfate assimilation	4	1.26650901	0.10260828	0.41043314	1.29553418	1.20066024	1.19666155	1.38226972	1.24719541	1.2861217
GO:0032312	regulation of ARF GTPase activity	14	1.06657186	0.02799012	0.39186168	1.13974365	1.05207695	0.95861833	1.12579694	1.09503334	1.03885013
GO:0006656	phosphatidylcholine biosynthetic process	1	2.4362818	0.38672752	0.38672752	2.51687502	2.42940678	2.19855589	2.62066002	2.47275418	2.40034737
GO:0006072	glycerol-3-phosphate metabolic process	7	1.13563088	0.05523719	0.38666036	1.22719494	1.10402938	1.01664464	1.20749521	1.16398422	1.1079682
GO:0015995	chlorophyll biosynthetic process	8	1.11645392	0.0478408	0.38272643	1.18708054	1.07756571	1.01173082	1.2005335	1.13099836	1.10209652
GO:0006471	protein ADP-ribosylation	4	1.24635563	0.09564198	0.38256792	1.31336362	1.16508498	1.1817216	1.33447414	1.23700454	1.25577741
GO:0000245	spliceosome assembly	2	1.52099487	0.18212775	0.3642555	1.59733834	1.50579673	1.41161337	1.57627491	1.55089228	1.4916738
GO:0006006	glucose metabolic process	8	1.11037849	0.04547104	0.36376833	1.17909748	1.09275392	0.97933278	1.20470799	1.13510501	1.0861906
GO:0000398	mRNA splicing%2C via spliceosome	8	1.10794144	0.04451681	0.35613447	1.17431742	1.07692126	1.00913874	1.18071994	1.12456543	1.09156321
GO:0005991	trehalose metabolic process	1	2.26937988	0.3559072	0.3559072	2.54175318	2.06891053	2.11453765	2.38527409	2.29317682	2.24582988

GO:0006857	oligopeptide transport	58	1.01421917	0.00613182	0.35564529	1.11326301	0.9887065	0.9185531	1.0465448	1.04913792	0.98046263
GO:0010338	leaf formation	1	2.23304131	0.34889676	0.34889676	2.4584653	2.12931233	2.01479169	2.35750934	2.28797738	2.17942429
GO:0006725	cellular aromatic compound metabolic process	3	1.29829037	0.11337184	0.34011551	1.4027178	1.26110471	1.17865037	1.36263923	1.33002783	1.26731024
GO:0046168	glycerol-3-phosphate catabolic process	4	1.21033797	0.08290666	0.33162663	1.30428824	1.18047197	1.08365302	1.28619591	1.2408367	1.18058887
GO:0006108	malate metabolic process	13	1.06046229	0.02549523	0.33143801	1.10548745	1.02669123	0.94631136	1.17747909	1.0653611	1.05558602
GO:0019752	carboxylic acid metabolic process	19	1.03787229	0.01614392	0.30673444	1.09516591	1.01482588	0.93529818	1.11623114	1.05423086	1.02176756
GO:0009073	aromatic amino acid family biosynthetic process	10	1.07283748	0.03053394	0.30533937	1.12826222	1.04444526	0.97013315	1.15880051	1.08554508	1.06027864
GO:0009725	response to hormone	21	1.03344728	0.01428833	0.30005487	1.0936928	0.99825021	0.93511772	1.11725488	1.04488232	1.02213739
GO:0000077	DNA damage checkpoint	3	1.25112003	0.09729898	0.29189693	1.38972037	1.24850059	1.08109749	1.30621563	1.31721931	1.18833768
GO:0006816	calcium ion transport	1	1.93845677	0.28745612	0.28745612	2.06468485	1.88122308	1.77612123	2.04671703	1.97082034	1.90662465
GO:0000154	rRNA modification	4	1.17820982	0.07122264	0.28489055	1.2960639	1.13914881	1.04458516	1.24951024	1.21507598	1.14246219
GO:0042398	cellular modified amino acid biosynthetic process	7	1.09700369	0.04020809	0.28145661	1.21089051	1.04506333	0.9719002	1.17750618	1.12492545	1.06977497
GO:0000737	DNA catabolic process%2C endonucleolytic	3	1.23969546	0.09331501	0.27994503	1.29622742	1.20159014	1.11737856	1.35713218	1.24801205	1.23143429
GO:0006221	pyrimidine nucleotide biosynthetic process	8	1.08351086	0.03483327	0.27866615	1.15154672	1.05793231	0.9707671	1.16540958	1.10374747	1.06364528
GO:0009081	branched-chain amino acid metabolic process	5	1.13191286	0.053813	0.26906498	1.18446257	1.10284997	1.02168295	1.22998002	1.14292804	1.12100385
GO:0009107	lipoate biosynthetic process	5	1.12729616	0.05203803	0.26019015	1.20001334	1.12431511	1.01668987	1.17730638	1.16154773	1.0940546
GO:0006809	nitric oxide biosynthetic process	1	1.79723181	0.2546041	0.2546041	2.05761634	1.7004831	1.60173595	1.86161017	1.87054586	1.72679123
GO:0000162	tryptophan biosynthetic process	3	1.21143408	0.08329979	0.24989936	1.22536405	1.1830063	1.13075798	1.31394532	1.20399892	1.21891515
GO:0007049	cell cycle	7	1.08472363	0.0353191	0.24723371	1.13489263	1.06052506	0.98568995	1.16697181	1.09707889	1.07250752
GO:0030494	bacteriochlorophyll biosynthetic process	1	1.76651556	0.24711747	0.24711747	1.87248921	1.64582572	1.56829405	2.01483369	1.75550303	1.77759717
GO:0006621	protein retention in ER lumen	5	1.11779012	0.04836026	0.24180132	1.18578053	1.05954607	0.99473247	1.24913854	1.12088764	1.11470115
GO:0006644	phospholipid metabolic process	4	1.1482414	0.0600332	0.24013281	1.19447239	1.12441995	1.05529387	1.22646381	1.15891699	1.13766416
GO:0006873	cellular ion homeostasis	1	1.73535533	0.23938841	0.23938841	1.97544772	1.64432499	1.62492034	1.71817888	1.80229799	1.67089911
GO:0046373	L-arabinose metabolic process	5	1.11623807	0.04775683	0.23878414	1.16832149	1.06593876	1.07058915	1.16442054	1.11595661	1.11651959
GO:0009165	nucleotide biosynthetic process	6	1.09569719	0.03969055	0.23814328	1.16767053	1.06132882	0.98594617	1.17961071	1.11323061	1.07843992
GO:0006231	dTMP biosynthetic process	2	1.31194307	0.11791499	0.23582998	1.41020713	1.26000365	1.18449197	1.40758013	1.33299142	1.29122708
GO:0006545	glycine biosynthetic process	2	1.31194307	0.11791499	0.23582998	1.41020713	1.26000365	1.18449197	1.40758013	1.33299142	1.29122708
GO:0009793	embryo development ending in seed dormancy	1	1.70211848	0.23098979	0.23098979	1.75529214	1.70838122	1.52607245	1.83421314	1.73167784	1.6730637
GO:0045226	extracellular polysaccharide biosynthetic process	3	1.19142497	0.0760667	0.2282001	1.26360654	1.1731732	1.0647779	1.27653806	1.21755054	1.16585999
GO:0009269	response to desiccation	5	1.11016725	0.04538841	0.22694207	1.17176901	1.08757762	1.01075613	1.1792476	1.12888872	1.09175627
GO:0010038	response to metal ion	3	1.1888003	0.07510891	0.22532672	1.23700065	1.14769148	1.07078734	1.31382436	1.19150959	1.18609717
GO:0006909	phagocytosis	7	1.07691268	0.03218049	0.22526344	1.12181847	1.04566954	1.02943698	1.11379473	1.08307497	1.07078545
GO:0018342	protein prenylation	3	1.18645014	0.07424949	0.22274848	1.310598	1.16348917	1.04197651	1.24711988	1.23485488	1.13994282
GO:0010315	auxin efflux	1	1.6700914	0.22274024	0.22274024	1.58168684	1.7153941	1.5530953	1.84619871	1.64718435	1.69331702
GO:0006595	polyamine metabolic process	1	1.66946552	0.22257745	0.22257745	1.7446652	1.60418896	1.47493581	1.88178097	1.67295327	1.66598504
GO:0120029	proton export across plasma membrane	1	1.66394326	0.22113851	0.22113851	1.77418259	1.52839009	1.48917753	1.89834462	1.64670674	1.68136021
GO:0030245	cellulose catabolic process	1	1.65919842	0.21989833	0.21989833	1.5613462	1.65043411	1.57907651	1.86248477	1.60527226	1.71493614
GO:0016573	histone acetylation	3	1.18352387	0.07317702	0.21953106	1.2508192	1.16004514	1.08016176	1.25184298	1.20457741	1.1628383
GO:0015977	carbon fixation	9	1.05748946	0.02427605	0.21848441	1.12406376	1.02164887	0.95001996	1.14624868	1.07163355	1.04353204
GO:0071704	organic substance metabolic process	1	1.63986692	0.2148086	0.2148086	1.58742028	1.66662456	1.46738355	1.86277788	1.62654039	1.65330264
GO:0000272	polysaccharide catabolic process	11	1.04561118	0.01937022	0.21307239	1.15839019	1.03144167	0.92054913	1.08676177	1.09307452	1.00020878
GO:0006561	proline biosynthetic process	5	1.10053819	0.04160512	0.20802559	1.16922528	1.07217777	1.00967476	1.15897475	1.11965055	1.08175207
GO:0006007	glucose catabolic process	3	1.16878124	0.06773323	0.2031997	1.38570088	1.09536013	0.98971219	1.24221734	1.2320071	1.10880009
GO:0015746	citrate transport	5	1.09443584	0.03919031	0.19595153	1.19524811	1.05346728	0.95069854	1.19850342	1.12212066	1.06743405
GO:0019295	coenzyme M biosynthetic process	1	1.56429016	0.19431731	0.19431731	1.61624384	1.53348597	1.39708936	1.72925237	1.5743212	1.55432303
GO:0042546	cell wall biogenesis	19	1.02357823	0.01012104	0.19229976	1.11619061	0.98502178	0.92350071	1.08109217	1.04855714	0.99919437
GO:0043043	peptide biosynthetic process	2	1.2448655	0.09512243	0.19024486	1.34506901	1.20041678	1.1168443	1.33174314	1.2706862	1.21956949
GO:1990426	mitotic recombination-dependent replication fork pro	2	1.24208944	0.09415287	0.18830574	1.26360987	1.20402253	1.14495865	1.36638805	1.23345642	1.25078288
GO:0048478	replication fork protection	1	1.53154977	0.18513111	0.18513111	1.64502671	1.45667655	1.37474729	1.67018855	1.54798961	1.51528452
GO:0006069	ethanol oxidation	1	1.52224291	0.18248396	0.18248396	1.50956507	1.4906179	1.39800364	1.70690505	1.50006157	1.54475224
GO:0015996	chlorophyll catabolic process	2	1.23364531	0.09119031	0.18238063	1.50764524	1.47793949	0.99250125	1.04730789	1.49271847	1.01953636
GO:0010274	hydrotropism	1	1.51639126	0.18081127	0.18081127	1.59831245	1.43432121	1.35666347	1.70006238	1.51409823	1.51868777
GO:0090116	C-5 methylation of cytosine	2	1.22912759	0.08959697	0.17919393	1.33913559	1.1788971	1.11518629	1.29640289	1.25646451	1.20238543
GO:0015969	guanosine tetraphosphate metabolic process	8	1.05174343	0.02190981	0.17527847	1.13279779	1.00608327	0.93820685	1.14433797	1.06756214	1.03615912
GO:0030261	chromosome condensation	2	1.21923154	0.08608619	0.17217238	1.31893463	1.18769517	1.09565145	1.28749063	1.2515959	1.18770408
GO:0009252	peptidoglycan biosynthetic process	1	1.48546629	0.1718628	0.1718628	1.51049702	1.37123917	1.33161381	1.76538662	1.43918473	1.53323618
GO:0006751	glutathione catabolic process	1	1.47292525	0.16818071	0.16818071	1.41887393	1.30921516	1.64457866	1.54068318	1.36294213	1.59178349
GO:0009067	aspartate family amino acid biosynthetic process	1	1.43405541	0.15656593	0.15656593	1.69542058	1.36117658	1.1069893	1.65549697	1.51913356	1.35374201
GO:0006098	pentose-phosphate shunt	10	1.03555752	0.01517423	0.15174228	1.0814052	1.0082693	0.92942949	1.13478992	1.04419714	1.02698939
GO:0046488	phosphatidylinositol metabolic process	15	1.0233384	0.01001927	0.15028903	1.10919867	0.99244893	0.91496793	1.088814	1.04920114	0.99811316
GO:0009688	abscisic acid biosynthetic process	1	1.41082613	0.14947349	0.14947349	1.42783997	1.43492517	1.2991412	1.48843142	1.43137819	1.39056916
GO:0008202	steroid metabolic process	2	1.18548726	0.07389689	0.14779378	1.27136855	1.16249162	1.06733526	1.25206077	1.21571184	1.1560141
GO:0006461	protein complex assembly	2	1.18361008	0.07320866	0.14641731	1.27398129	1.13224166	1.04715166	1.29934014	1.20102235	1.16645025
GO:0009873	ethylene mediated signaling pathway	2	1.17561457	0.07026496	0.14052992	1.30255558	1.16052837	1.06397442	1.18761759	1.22949286	1.1240973
GO:0032324	molybdopterin cofactor biosynthetic process	1	1.37757696	0.13911587	0.13911587	1.4178772	1.31476563	1.23780752	1.56071429	1.36534838	1.38991507
GO:0015986	ATP synthesis coupled proton transport	16	1.01997351	0.00858889	0.13742231	1.07454865	0.98616341	0.90209694	1.13221108	1.02940787	1.01062562
GO:0016125	sterol metabolic process	1	1.35796011	0.13288701	0.13288701	1.55700531	1.25383833	1.24882985	1.3948039	1.3972233	1.31980026
GO:0000045	autophagic vacuole assembly	1	1.35003174	0.13034398	0.13034398	1.309884	1	2.535964	1	1.14450164	1.59247104
GO:0006744	ubiquinone biosynthetic process	6	1.05096229	0.02158713	0.12952279	1.07633661	1.05571632	0.97208353	1.10445898	1.06597661	1.03615944
GO:0015940	pantothenate biosynthetic process	2	1.16013403	0.06450817	0.12901634	1.21623619	1.10593732	1.03319213	1.30347633	1.15977627	1.16049191
GO:0006354	transcription elongation%2C DNA-dependent	1	1.32441779	0.12202501	0.12202501	1.39275261	1.26380216	1.20838679	1.44657511	1.32671163	1.32212792
GO:0032784	regulation of transcription elongation%2C DNA-dep	1	1.32441779	0.12202501	0.12202501	1.39275261	1.26380216	1.20838679	1.44657511	1.32671163	1.32212792
GO:0015693	magnesium ion transport	1	1.31404131	0.11860902	0.11860902	1.39898994	1.24008243	1.26440211	1.35920733	1.31714193	1.31094798
GO:0006101	citrate metabolic process	1	1.30720759	0.11634456	0.11634456	1.35060785	1.28635011	1.16914798	1.43754121	1.31808746	1.29641753
GO:0006379	mRNA cleavage	1	1.29867065	0.11349903	0.11349903	1.41001068	1.23515351	1.13960929	1.43316695	1.31968922	1.27798684
GO:0006566	threonine metabolic process	1	1.29161256	0.11113226	0.11113226	1.37701671	1.2460831	1.16543084	1.39173426	1.30991497	1.27356587
GO:0006672	ceramide metabolic process	2	1.12875317	0.05259898	0.10519796	1.20198763	1.07978704	1.00912102	1.23940877	1.13925005	1.118353
GO:0009439	cyanate metabolic process	1	1.24951958	0.09674307	0.09674307	1.31269361	1.20462699	1.1408074	1.35127574	1.25749996	1.24158985
GO:0006269	DNA replication%2C synthesis of RNA primer	1	1.2239193	0.08775278	0.08775278	1.33428927	1.16348007	1.10611451	1.30677885	1.24596107	1.20226746
GO:0006808	regulation of nitrogen utilization	1	1.22184247	0.08701522	0.08701522	1.28606316	1.17413327	1.11097517	1.32854668	1.22882446	1.21490015
GO:0009306	protein secretion	2	1.10452051	0.04317379	0.08634757	1.15492452	1.05623239	1.00824293	1.21008778	1.10447666	1.10456437
GO:0006261	DNA-dependent DNA replication	1	1.21754096	0.08548358	0.08548358	1.39485733	1.06823564	1.10833752	1.33065488	1.22067043	1.2144195
GO:0009910	negative regulation of flower development	1	1.21325526	0.08395218	0.08395218	1.39073093	1.25202086	0.96532984	1.28907542	1.31955452	1.11551915
GO:0006659	phosphatidylserine biosynthetic process	2	1.1004271	0.04156128	0.08312255	1.18415113	1.06965515	0.99031257	1.16901986	1.12544807	1.07596239
GO:0043461	proton-transporting ATP synthase complex assembl	1	1.20640648	0.08149366	0.08149366	1.27405542	1.15338947	1.09420443	1.31738246	1.21222197	1.2006189

GO:0006750	glutathione biosynthetic process	6	1.03164197	0.013529	0.08117401	1.04563004	1.01146404	0.9661587	1.10850853	1.02840516	1.03488896
GO:0006302	double-strand break repair	2	1.09627793	0.03992067	0.07984134	1.13487082	1.055321	0.97551832	1.2362783	1.09437334	1.09818583
GO:0006481	C-terminal protein methylation	1	1.18506641	0.07374269	0.07374269	1.29301508	1.1062947	1.03615895	1.33066872	1.19601661	1.17421646
GO:0007021	tubulin complex assembly	1	1.18472601	0.07361792	0.07361792	1.23547069	1.16076095	1.03166579	1.33154983	1.19753336	1.17205563
GO:0006282	regulation of DNA repair	1	1.15931572	0.06420173	0.06420173	1.14254801	1.15496452	1.06218092	1.28874	1.14873948	1.16998933
GO:0009231	riboflavin biosynthetic process	8	1.01862857	0.00801585	0.06412684	1.08662193	0.98024865	0.89953531	1.12364851	1.03206573	1.00536636
GO:0006259	DNA metabolic process	17	1.00848136	0.00366788	0.06235389	1.06371007	0.98892009	0.90183072	1.09034016	1.02563359	0.99161598
GO:0006306	DNA methylation	7	1.02040406	0.00877218	0.06140523	1.07029622	0.98876747	0.94671824	1.08210599	1.02872449	1.01215092
GO:0006974	response to DNA damage stimulus	3	1.04816403	0.02042925	0.06128775	1.10557522	1.0027192	0.94460251	1.1526575	1.05289197	1.04345731
GO:0071985	multivesicular body sorting pathway	1	1.14859484	0.06016686	0.06016686	1.23949639	1.1051817	1.03781047	1.22425092	1.17041391	1.12718252
GO:0006348	chromatin silencing at telomere	1	1.14713445	0.05961432	0.05961432	1.20189873	1.13194454	1.05636594	1.20489685	1.16639732	1.1281897
GO:0016568	chromatin modification	1	1.14713445	0.05961432	0.05961432	1.20189873	1.13194454	1.05636594	1.20489685	1.16639732	1.1281897
GO:0051252	regulation of RNA metabolic process	3	1.04261257	0.01812296	0.05436887	1.12377879	1.04208816	0.94819515	1.06416458	1.08216292	1.00450768
GO:0009103	lipopolysaccharide biosynthetic process	1	1.12433048	0.05089398	0.05089398	1.18663772	1.08396127	0.99176536	1.25266541	1.13413814	1.11460763
GO:0045132	meiotic chromosome segregation	1	1.1222428	0.05008683	0.05008683	1.27224423	1.1470102	1.05684606	1.02848465	1.20800543	1.04256892
GO:0046938	phytochelatin biosynthetic process	2	1.05550995	0.02346233	0.04692467	1.09919254	1.02256982	0.94960337	1.16289456	1.06018919	1.05085137
GO:0007047	cellular cell wall organization	1	1.11286939	0.0464442	0.0464442	1.14277695	1.09593935	1.01359164	1.20827499	1.11911314	1.10666048
GO:0010508	positive regulation of autophagy	2	1.05443293	0.02301896	0.04603792	1.11809547	1.01918184	0.94691775	1.14560005	1.06749361	1.04153206
GO:0007062	sister chromatid cohesion	1	1.1100559	0.04534485	0.04534485	1.17915758	1.09811996	0.99917954	1.17358416	1.1379176	1.08287639
GO:0007067	mitosis	11	1.00945881	0.0040886	0.04497461	1.0539099	1.03910968	0.89992301	1.0536204	1.04648363	0.97374393
GO:0006166	purine ribonucleoside salvage	2	1.04971656	0.02107205	0.0421441	1.07245809	1.02537613	0.94141205	1.17285656	1.04865291	1.05078128
GO:0006570	tyrosine metabolic process	1	1.0872198	0.03631735	0.03631735	1.11936612	1.09710754	0.98118263	1.15957331	1.10818094	1.06665514
GO:0015074	DNA integration	6	1.01308249	0.00564481	0.03386884	1.20887543	0.96619434	0.89254833	1.01041887	1.08074446	0.94965661
GO:0006555	methionine metabolic process	1	1.07288318	0.03055244	0.03055244	1.14543512	1.03408082	0.9549195	1.17143449	1.08833473	1.057651
GO:0018106	peptidyl-histidine phosphorylation	7	1.00945881	0.0040886	0.02862022	1.07266451	0.98920674	0.91091209	1.07430343	1.03009075	0.98924011
GO:0022904	respiratory electron transport chain	1	1.06119461	0.02579504	0.02579504	1.06787224	1.03298119	0.97269106	1.18193477	1.05028184	1.07222077
GO:0007186	G-protein coupled receptor signaling pathway	13	1.00438247	0.00189912	0.0246886	1.16097268	0.9520817	0.8830529	1.04258999	1.05135191	0.95951139
GO:0009156	ribonucleoside monophosphate biosynthetic proces	2	1.02744205	0.01175734	0.02351467	1.09296962	0.98997929	0.92218032	1.11680999	1.0402006	1.01483998
GO:0006665	sphingolipid metabolic process	6	1.00861939	0.00372731	0.02236388	1.07900249	0.97730255	0.90251722	1.08743243	1.02689429	0.99066972
GO:0006591	ornithine metabolic process	1	1.04674327	0.01984018	0.01984018	1.13534279	0.99668076	0.93226525	1.13798917	1.06375482	1.03000377
GO:0006303	double-strand break repair via nonhomologous end	1	1.04670446	0.01982407	0.01982407	1.07727061	0.98813293	0.90108228	1.25138706	1.03173958	1.06188639
GO:0019288	isopentenyl diphosphate biosynthetic process%2C n	2	1.02167122	0.00931116	0.01862232	1.23653214	0.9462306	0.90931719	1.0240634	1.08168598	0.96498624
GO:0050992	dimethylallyl diphosphate biosynthetic process	2	1.02167122	0.00931116	0.01862232	1.23653214	0.9462306	0.90931719	1.0240634	1.08168598	0.96498624
GO:0006749	glutathione metabolic process	1	1.04370172	0.0185764	0.0185764	1.09886683	1.06517415	0.91969414	1.10229159	1.08188934	1.00686201
GO:0000724	double-strand break repair via homologous recombi	3	1.0139307	0.00600827	0.01802482	1.0739952	0.97489106	0.90096165	1.12038776	1.02324402	1.00470215
GO:0044238	primary metabolic process	1	1.03952652	0.01683557	0.01683557	1.11334977	1.00050507	0.92230323	1.13662615	1.05542034	1.02387205
GO:0006223	uracil salvage	1	1.03604662	0.0153793	0.0153793	1.13282779	1.02803894	0.91617311	1.07985685	1.07916221	0.99465361
GO:0006139	nucleobase-containing compound metabolic proces	44	1.00074787	0.00032468	0.01428574	1.07643032	0.98333007	0.89866098	1.05442957	1.02882764	0.97343449
GO:0019673	GDP-mannose metabolic process	1	1.02778112	0.01190064	0.01190064	1.1418129	0.92578034	0.87516749	1.20616984	1.02813809	1.02742427
GO:0006680	glucosylceramide catabolic process	2	1.0127127	0.00548626	0.01097251	1.13903625	0.96552287	0.90311308	1.05901664	1.04869707	0.97796308
GO:0031929	TOR signaling	1	1.02550138	0.01093625	0.01093625	1.07625616	0.98093084	0.91285694	1.14759389	1.02748862	1.02351797
GO:0009186	deoxyribonucleoside diphosphate metabolic proces	1	1.02104402	0.00904447	0.00904447	1.0598692	1.0276315	0.91327444	1.09266462	1.04362588	0.99895079
GO:0006476	protein deacetylation	1	1.0192588	0.00828447	0.00828447	1.10362337	0.99796264	0.8888671	1.1024677	1.04946409	0.98992286
GO:0045017	glycerolipid biosynthetic process	1	1.01920628	0.00826209	0.00826209	1.06875202	1.05662533	0.90716537	1.05332873	1.06267138	0.97751898
GO:0046034	ATP metabolic process	7	1.00232456	0.00100837	0.00705862	1.06161017	0.95866254	0.90576012	1.09493785	1.00882402	0.99586698
GO:0030488	tRNA methylation	1	1.01578294	0.00680092	0.00680092	1.02150643	0.99297809	0.93576673	1.12164465	1.00714125	1.02449878
GO:0044205	'de novo' UMP biosynthetic process	1	1.01410743	0.00608396	0.00608396	1.02898827	0.99448687	0.92233923	1.12056151	1.01159049	1.01663063
GO:0032502	developmental process	1	1.01289647	0.00556506	0.00556506	1.10933306	0.96316352	0.91488271	1.07679448	1.03366781	0.99254252
GO:0019370	leukotriene biosynthetic process	1	1.0124926	0.00539186	0.00539186	1.07605928	0.98760006	0.90188796	1.09647199	1.03088128	0.99443194
GO:0045010	actin nucleation	1	1.01147144	0.00495362	0.00495362	1.08208339	0.91499096	0.96499115	1.09550315	0.99503594	1.02817841
GO:0019277	UDP-N-acetylgalactosamine biosynthetic process	1	1.01139344	0.00492013	0.00492013	0.97218424	1.05117724	0.9289886	1.10216245	1.01090947	1.01187764
GO:0008615	pyridoxine biosynthetic process	1	1.00628959	0.00272298	0.00272298	1.05513483	0.96729322	0.90453395	1.11071069	1.01025975	1.00233504
GO:2000028	regulation of photoperiodism%2C flowering	1	1.00059503	0.00025834	0.00025834	1.05878714	0.96922981	0.89185313	1.09522816	1.01301928	0.98832316
GO:0016068	type I hypersensitivity	4	1	0	0	1	1	1	1	1	1
GO:0015684	ferrous iron transport	1	1	0	0	1	1	1	1	1	1
GO:0015851	nucleobase transport	1	1	0	0	1	1	1	1	1	1
GO:0009311	oligosaccharide metabolic process	1	1	0	0	1	1	1	1	1	1
GO:0015743	malate transport	1	1	0	0	1	1	1	1	1	1
GO:0046148	pigment biosynthetic process	1	1	0	0	1	1	1	1	1	1
GO:0006313	transposition%2C DNA-mediated	1	1	0	0	1	1	1	1	1	1
GO:0051013	microtubule severing	3	0.99694114	-0.0013305	-0.0039914	1.05643049	0.97913509	0.87383048	1.09286707	1.01704875	0.97723106
GO:0043401	steroid hormone mediated signaling pathway	1	0.98529287	-0.0064347	-0.0064347	1.0015227	0.96870207	0.93175489	1.04257823	0.98497569	0.98561015
GO:0006788	heme oxidation	2	0.99239301	-0.0033163	-0.0066326	1.02743288	1.00565286	0.89608301	1.04757465	1.01648454	0.96887247
GO:0016043	cellular component organization	11	0.99814798	-0.0008051	-0.0088558	1.03838156	0.97411413	0.91113823	1.07703209	1.00573463	0.99061855
GO:0031047	gene silencing by RNA	4	0.99457433	-0.0023628	-0.009451	0.98454302	1.20262844	0.88561202	0.93312391	1.08813576	0.90905762
GO:0009972	cytidine deamination	1	0.97776178	-0.0097669	-0.0097669	1.03682032	0.91381763	0.82934625	1.16314355	0.97337797	0.98216533
GO:0051103	DNA ligation involved in DNA repair	2	0.98734514	-0.005531	-0.011062	1.03917234	0.9476566	0.89840584	1.07414976	0.99236008	0.98235554
GO:0006694	steroid biosynthetic process	11	0.99737121	-0.0011432	-0.0125749	1.09448057	0.96960762	0.87437045	1.06641871	1.03015372	0.96563192
GO:0009611	response to wounding	4	0.99278201	-0.0031461	-0.0125844	1.10447145	0.91981442	0.85599565	1.11709299	1.00792299	0.97786847
GO:0015936	coenzyme A metabolic process	3	0.98998521	-0.0043713	-0.0131139	1.02721667	0.98553358	0.89888094	1.05555086	1.00615929	0.97407112
GO:0006102	isocitrate metabolic process	4	0.98844452	-0.0050477	-0.0201908	1.03290062	0.90969009	0.89640516	1.13332074	0.96933971	1.00792587
GO:0017004	cytochrome complex assembly	4	0.98767885	-0.0053842	-0.021537	1.02857201	0.97597161	0.86553974	1.09522695	1.00192668	0.97363363
GO:0006779	porphyrin-containing compound biosynthetic proces	5	0.98986921	-0.0044222	-0.0221109	1.03262795	0.96867787	0.89406983	1.07353591	1.00014191	0.97970203
GO:0006741	NADP biosynthetic process	1	0.94887903	-0.0227892	-0.0227892	0.98220683	0.91697614	0.85899763	1.04782908	0.9490312	0.94872688
GO:0019674	NAD metabolic process	1	0.94887903	-0.0227892	-0.0227892	0.98220683	0.91697614	0.85899763	1.04782908	0.9490312	0.94872688
GO:0010223	secondary shoot formation	1	0.94776495	-0.0232994	-0.0232994	1.01546587	0.88808569	0.83044644	1.07738457	0.94964241	0.94589121
GO:1901601	strigolactone biosynthetic process	1	0.94776495	-0.0232994	-0.0232994	1.01546587	0.88808569	0.83044644	1.07738457	0.94964241	0.94589121
GO:0018343	protein farnesylation	1	0.94427686	-0.0249007	-0.0249007	0.93625266	0.93435111	0.83440034	1.08923039	0.9353014	0.95333845
GO:0009088	threonine biosynthetic process	3	0.98066488	-0.0084794	-0.0254381	1.0167355	0.94525314	0.88648246	1.08556599	0.98034301	0.98098686
GO:0009446	putrescine biosynthetic process	1	0.94111072	-0.0263593	-0.0263593	0.99896235	0.90438642	0.84034839	1.03323798	0.95049881	0.93181536
GO:0031167	rRNA methylation	1	0.93981682	-0.0269568	-0.0269568	1.00175283	0.92385461	0.82666954	1.01971008	0.96201558	0.9181303
GO:0006885	regulation of pH	5	0.98751169	-0.0054578	-0.0272888	1.037606	0.93386311	0.87501952	1.12159365	0.98436881	0.99066459
GO:0080009	mRNA methylation	1	0.93860218	-0.0275184	-0.0275184	0.86735332	0.9446693	0.87367163	1.0841819	0.90518619	0.97325175
GO:0019430	removal of superoxide radicals	3	0.97885601	-0.0092812	-0.0278436	1.03586196	0.93879971	0.87857955	1.07453192	0.98613737	0.97162841
GO:0007010	cytoskeleton organization	10	0.99328917	-0.0029243	-0.029243	1.11037736	0.94631702	0.88801213	1.04322179	1.02507024	0.96249343

GO:0043412	macromolecule modification	2	0.96526873	-0.0153518	-0.0307035	1.02575338	0.92793813	0.85115198	1.07157817	0.97562066	0.95502664
GO:0006506	GPI anchor biosynthetic process	13	0.99446197	-0.0024118	-0.0313536	1.07391897	0.94877756	0.88555971	1.08392431	1.00941083	0.9797345
GO:0071705	nitrogen compound transport	1	0.93024136	-0.0314044	-0.0314044	0.99192542	0.90566753	0.84331492	0.98842784	0.94781572	0.91299285
GO:0048280	vesicle fusion with Golgi apparatus	1	0.9286261	-0.0321591	-0.0321591	0.97684602	0.90792186	0.83666993	1.00215478	0.9417536	0.91568159
GO:0046856	phosphatidylinositol dephosphorylation	1	0.92672057	-0.0330512	-0.0330512	0.99749619	0.86419916	0.80447508	1.06354905	0.9284586	0.92498579
GO:0006839	mitochondrial transport	3	0.97445238	-0.0112394	-0.0337181	1.03984462	0.94075875	0.86211689	1.06912778	0.98906164	0.96005891
GO:0006233	dTDP biosynthetic process	1	0.9245684	-0.034061	-0.034061	0.99614791	0.86206265	0.81368085	1.0457778	0.92668329	0.92245833
GO:0017038	protein import	2	0.96008721	-0.0176893	-0.0353786	1.01068303	0.94852521	0.8768211	1.0108062	0.97911099	0.94143306
GO:0009236	cobalamin biosynthetic process	1	0.91797923	-0.0371671	-0.0371671	0.9268541	0.94336102	0.81389598	0.99786829	0.93507113	0.90119975
GO:0006032	chitin catabolic process	17	0.99482039	-0.0022553	-0.0383405	1.04238043	0.97572731	0.86771628	1.1098039	1.00850337	0.98132304
GO:0000042	protein targeting to Golgi	1	0.91245325	-0.0397894	-0.0397894	1.15650258	0.81029332	0.76510267	0.96679393	0.96804252	0.86005617
GO:0006801	superoxide metabolic process	8	0.98725239	-0.0055718	-0.0445745	1.03638101	0.96013539	0.88211018	1.08227595	0.99752999	0.97708067
GO:0010029	regulation of seed germination	1	0.90139507	-0.0450848	-0.0450848	0.89075757	0.84337559	0.77527884	1.13350188	0.86674286	0.93743268
GO:0006086	acetyl-CoA biosynthetic process from pyruvate	1	0.89935813	-0.0460673	-0.0460673	0.96738992	0.82397544	0.80976898	1.0135699	0.89280767	0.90595665
GO:0046854	phosphatidylinositol phosphorylation	3	0.96521455	-0.0153761	-0.0461284	1.04114475	0.9392776	0.85698229	1.03566315	0.98890037	0.94209606
GO:0048015	phosphatidylinositol-mediated signaling	3	0.96521455	-0.0153761	-0.0461284	1.04114475	0.9392776	0.85698229	1.03566315	0.98890037	0.94209606
GO:0010024	phytochromobilin biosynthetic process	1	0.89635433	-0.0475203	-0.0475203	0.97154593	0.85984948	0.79562112	0.97124054	0.91399304	0.87905602
GO:0006729	tetrahydrobiopterin biosynthetic process	2	0.94579951	-0.0242009	-0.0484018	1.06688428	0.95586207	0.85698178	0.91561354	1.00984861	0.88581269
GO:0009245	lipid A biosynthetic process	3	0.96235416	-0.0166651	-0.0499952	1.032494	0.93385315	0.86024825	1.03406964	0.98193573	0.94316308
GO:0051205	protein insertion into membrane	5	0.97601231	-0.0105447	-0.0527235	1.04634235	0.96513722	0.89901289	0.99952206	1.00491987	0.94793629
GO:0043085	positive regulation of catalytic activity	1	0.88460368	-0.0532513	-0.0532513	0.9505454	0.86547615	0.78595642	0.94704045	0.90701398	0.86274708
GO:0031145	anaphase-promoting complex-dependent proteasor	2	0.9396948	-0.0270132	-0.0540263	1.03190449	0.95983079	0.81025109	0.97161341	0.99521541	0.88727156
GO:0019478	D-amino acid catabolic process	1	0.88229441	-0.0543865	-0.0543865	1.02272126	0.85267389	0.7804765	0.89033619	0.93383495	0.83359851
GO:0006891	intra-Golgi vesicle-mediated transport	1	0.87769721	-0.0566553	-0.0566553	0.90572323	0.83641624	0.78722606	0.99508792	0.87038016	0.88507578
GO:0042819	vitamin B6 biosynthetic process	3	0.95722106	-0.0189878	-0.0569633	1.08997701	1.15926062	0.7197688	0.92311867	1.12408515	0.81512699
GO:0042823	pyridoxal phosphate biosynthetic process	3	0.95722106	-0.0189878	-0.0569633	1.08997701	1.15926062	0.7197688	0.92311867	1.12408515	0.81512699
GO:0006623	protein targeting to vacuole	1	0.87553516	-0.0577264	-0.0577264	0.936254	0.83434824	0.76067386	0.98890562	0.88383362	0.86731462
GO:0032786	positive regulation of transcription elongation%2C D	1	0.86875496	-0.0611027	-0.0611027	0.96780064	0.83498693	0.7594004	0.92822398	0.89894432	0.83957946
GO:0010027	thylakoid membrane organization	1	0.86560228	-0.0626816	-0.0626816	0.84477565	0.85586535	0.79669118	0.97462316	0.85030242	0.88117744
GO:0071669	plant-type cell wall organization or biogenesis	1	0.86005914	-0.0654717	-0.0654717	0.89109947	0.82389398	0.78282849	0.9520267	0.85683807	0.86329232
GO:0006275	regulation of DNA replication	1	0.85901933	-0.0659971	-0.0659971	0.92402725	0.80864326	0.76532694	0.95218861	0.86441218	0.85366012
GO:0016973	poly(A)+ mRNA export from nucleus	1	0.85577698	-0.0676394	-0.0676394	0.88875058	0.81836944	0.75292496	0.97940295	0.85283428	0.85872983
GO:0050790	regulation of catalytic activity	1	0.85384003	-0.0686235	-0.0686235	0.95720408	0.79026933	0.76251462	0.92146366	0.86974078	0.83822999
GO:0010167	response to nitrate	1	0.85316253	-0.0689682	-0.0689682	0.92408074	0.80027744	0.7760463	0.92318548	0.85995405	0.84642464
GO:0015706	nitrate transport	1	0.85316253	-0.0689682	-0.0689682	0.92408074	0.80027744	0.7760463	0.92318548	0.85995405	0.84642464
GO:0009094	L-phenylalanine biosynthetic process	6	0.97368115	-0.0115832	-0.0694994	1.04836454	0.97383763	0.87441689	1.00681492	1.01041419	0.93828352
GO:0006177	GMP biosynthetic process	1	0.85116317	-0.0699872	-0.0699872	1.01938031	0.98888553	0.88559445	0.58794154	1.00401715	0.72158005
GO:0009097	isoleucine biosynthetic process	2	0.91775594	-0.0372728	-0.0745456	0.92602422	0.9052137	0.82991288	1.01977145	0.91555984	0.91995731
GO:0006208	pyrimidine base catabolic process	1	0.8407087	-0.0753545	-0.0753545	0.89469228	0.81963034	0.73923841	0.92152247	0.85633926	0.82536343
GO:0006333	chromatin assembly or disassembly	2	0.91651771	-0.0378591	-0.0757183	0.97533047	0.83557535	0.86539458	1.00048797	0.90275251	0.93049281
GO:0006163	purine nucleotide metabolic process	2	0.91539081	-0.0383935	-0.0767869	0.93558346	0.85574862	0.83041093	1.05609841	0.89477609	0.93648046
GO:0008616	queuosine biosynthetic process	2	0.91450114	-0.0388157	-0.0776315	0.94256869	0.90176611	0.83175335	0.98931711	0.9219417	0.90712062
GO:0051090	regulation of sequence-specific DNA binding transc	1	0.83563828	-0.0779817	-0.0779817	0.8781005	0.80047585	0.73495778	0.94388363	0.83839027	0.83289532
GO:0045900	negative regulation of translational elongation	1	0.82914658	-0.0813687	-0.0813687	0.83301996	0.79563506	0.74922804	0.95179168	0.81411294	0.84445782
GO:0060003	copper ion export	1	0.82866151	-0.0816228	-0.0816228	0.87020754	0.89415981	0.69464546	0.87238355	0.88210238	0.77845827
GO:0016598	protein arginylation	1	0.82409067	-0.084025	-0.084025	0.87938508	0.81093775	0.76371388	0.84684259	0.84446821	0.80420485
GO:0015671	oxygen transport	3	0.93643157	-0.028524	-0.0855719	0.99397982	0.88789082	0.85041741	1.02455414	0.93943896	0.93343381
GO:0005986	sucrose biosynthetic process	2	0.9039739	-0.0438441	-0.0876882	0.95107151	0.87107885	0.81173739	0.99297286	0.91019683	0.89779351
GO:0006423	cysteinyl-tRNA aminoacylation	3	0.93011539	-0.0314632	-0.0943895	0.97508696	0.9256066	0.85990769	0.96433004	0.9500247	0.91062331
GO:0007166	cell surface receptor signaling pathway	1	0.80075082	-0.0965026	-0.0965026	0.86389091	0.75488328	0.71844237	0.87752383	0.80754988	0.79400901
GO:0006383	transcription by RNA polymerase III	1	0.80019987	-0.0968015	-0.0968015	0.87262092	0.76975623	0.7144335	0.85438432	0.81957635	0.7812815
GO:0071267	L-methionine salvage	1	0.79968446	-0.0970813	-0.0970813	0.84223376	0.76212377	0.70538827	0.90320895	0.80117811	0.79819358
GO:0006433	prolyl-tRNA aminoacylation	2	0.89392776	-0.0486976	-0.0973952	0.95572223	0.85607103	0.80011316	0.97547646	0.90452535	0.88345433
GO:0006559	L-phenylalanine catabolic process	9	0.97531929	-0.0108532	-0.0976787	1.08455944	0.94861694	0.88167415	0.99755056	1.01431329	0.93782437
GO:0048278	vesicle docking	1	0.7982187	-0.0978781	-0.0978781	0.83233107	0.78842199	0.71789939	0.86172595	0.81007908	0.78653197
GO:0042450	arginine biosynthetic process via ornithine	1	0.79817725	-0.0979007	-0.0979007	0.83208821	0.77400409	0.71594962	0.88024243	0.80252082	0.79385719
GO:0009443	pyridoxal 5'-phosphate salvage	1	0.79539413	-0.0994176	-0.0994176	0.77642328	0.78934156	0.72960543	0.89511319	0.78285578	0.80813331
GO:0032955	regulation of barrier septum formation	1	0.7933112	-0.1005564	-0.1005564	0.83145397	0.76361307	0.70320036	0.8871227	0.79681185	0.78982593
GO:0051301	cell division	1	0.7933112	-0.1005564	-0.1005564	0.83145397	0.76361307	0.70320036	0.8871227	0.79681185	0.78982593
GO:1902600	proton transmembrane transport	2	0.89041011	-0.0504099	-0.1008198	0.91970919	0.89106333	0.84336727	0.90946186	0.90527296	0.87579128
GO:0006188	IMP biosynthetic process	1	0.79157775	-0.1015064	-0.1015064	0.84254456	0.74924948	0.70911482	0.87707841	0.79452884	0.78863762
GO:0009152	purine ribonucleotide biosynthetic process	1	0.79157775	-0.1015064	-0.1015064	0.84254456	0.74924948	0.70911482	0.87707841	0.79452884	0.78863762
GO:0007131	reciprocal meiotic recombination	3	0.92493496	-0.0338888	-0.1016664	0.99972515	0.89693444	0.82412034	0.99040501	0.94693607	0.90344502
GO:0019836	hemolysis by symbiont of host erythrocytes	1	0.78786192	-0.1035499	-0.1035499	0.85972139	0.74707536	0.70307884	0.85324603	0.80142166	0.77453162
GO:0006814	sodium ion transport	16	0.98497542	-0.0065746	-0.1051937	1.04342782	0.94586703	0.89736422	1.06277256	0.99345054	0.97657261
GO:0045040	protein import into mitochondrial outer membrane	2	0.88223259	-0.0544169	-0.1088338	0.92228043	0.85984996	0.79417219	0.96190469	0.89051827	0.874024
GO:0006777	Mo-molybdopterin cofactor biosynthetic process	6	0.95887107	-0.0182398	-0.1094387	1.01205759	0.93349956	0.8817372	1.01480443	0.97198524	0.94593384
GO:0006014	D-ribose metabolic process	3	0.91903691	-0.036667	-0.1100011	0.9638555	0.88223119	0.83349682	1.00654577	0.92214065	0.91594361
GO:0070481	nuclear-transcribed mRNA catabolic process%2C nc	1	0.77488113	-0.1107649	-0.1107649	0.8253121	0.72239295	0.72190315	0.83766369	0.77213965	0.77763234
GO:0070966	nuclear-transcribed mRNA catabolic process%2C nc	1	0.77488113	-0.1107649	-0.1107649	0.8253121	0.72239295	0.72190315	0.83766369	0.77213965	0.77763234
GO:0071025	RNA surveillance	1	0.77488113	-0.1107649	-0.1107649	0.8253121	0.72239295	0.72190315	0.83766369	0.77213965	0.77763234
GO:0006499	N-terminal protein myristoylation	1	0.770786	-0.1130662	-0.1130662	0.80867129	0.75418572	0.67257374	0.86048842	0.78095348	0.76075089
GO:0030041	actin filament polymerization	1	0.76597926	-0.115783	-0.115783	0.83233404	0.71604789	0.67261802	0.85873628	0.77200455	0.76000099
GO:0006568	tryptophan metabolic process	9	0.97080485	-0.0128681	-0.1158126	1.04213032	0.92816731	0.87714476	1.04690718	0.98349951	0.95827405
GO:0006446	regulation of translational initiation	1	0.7620043	-0.1180426	-0.1180426	0.78844094	0.71666944	0.67347024	0.8859786	0.75169909	0.77245079
GO:0015976	carbon utilization	3	0.91225881	-0.0398819	-0.1196458	0.93341687	0.90212562	0.8551627	0.9617913	0.91763788	0.90691127
GO:0006826	iron ion transport	2	0.870955	-0.0600043	-0.1200086	0.94227515	0.8341664	0.82177905	0.89083519	0.88657446	0.85561072
GO:0045980	negative regulation of nucleotide metabolic process	1	0.75627654	-0.1213194	-0.1213194	0.78985561	0.7266875	0.68092315	0.83700688	0.75761349	0.75494195
GO:0006436	tryptophanyl-tRNA aminoacylation	2	0.86837293	-0.0612937	-0.1225874	0.9060275	0.83487311	0.78093117	0.96261027	0.86972295	0.86702501
GO:0048034	heme O biosynthetic process	1	0.75272802	-0.1233619	-0.1233619	0.80230811	0.7242449	0.68297292	0.80895101	0.76227788	0.74329781
GO:0045944	positive regulation of transcription by RNA polymera	14	0.97985358	-0.0088388	-0.1237434	1.02418469	0.94863949	0.89473848	1.06039858	0.98568861	0.97405308
GO:0008612	peptidyl-lysine modification to hypusine	4	0.93105737	-0.0310236	-0.1240942	0.98673688	0.90584587	0.85230989	0.98639886	0.94542664	0.91690649
GO:0006596	polyamine biosynthetic process	3	0.90890192	-0.041483	-0.1244489	0.96846832	0.8793319	0.89323058	0.89715248	0.92282451	0.89518938
GO:0006897	endocytosis	2	0.86310574	-0.063936	-0.127872	0.89455967	0.84030357	0.78880472	0.93592489	0.86700731	0.85922172
GO:0006122	mitochondrial electron transport%2C ubiquinol to cy	6	0.95193795	-0.0213914	-0.1283482	0.98731798	0.92268019	0.85212361	1.05784907	0.95445206	0.94943045

GO:0006606	protein import into nucleus	2	0.86194438	-0.0645208	-0.1290415	0.91222791	0.85966517	0.76305773	0.92241613	0.88555664	0.83896171
GO:0006597	spermine biosynthetic process	3	0.90316204	-0.0442343	-0.132703	0.90691713	0.8797606	0.85690244	0.97319352	0.89323567	0.91319872
GO:0007030	Golgi organization	1	0.73603434	-0.1331019	-0.1331019	0.81849905	0.69993085	0.62675016	0.81738098	0.75689678	0.71574693
GO:0046080	dUTP metabolic process	1	0.73220677	-0.1353663	-0.1353663	0.76918021	0.66867618	0.76927516	0.72645598	0.71716977	0.74755905
GO:0006450	regulation of translational fidelity	1	0.72821485	-0.1377405	-0.1377405	0.77896466	0.70878218	0.65693358	0.77532915	0.74304527	0.71368043
GO:0015780	nucleotide-sugar transport	2	0.85002862	-0.0705665	-0.1411329	0.83507278	0.78069867	0.90209219	0.88771899	0.80742814	0.89487673
GO:0009228	thiamine biosynthetic process	4	0.92062267	-0.0359183	-0.1436733	0.9778355	0.90801424	0.8374182	0.9661083	0.94227839	0.89946466
GO:0006435	threonyl-tRNA aminoacylation	2	0.84332751	-0.0740037	-0.1480075	0.90123689	0.81607558	0.7712709	0.89167948	0.8575998	0.82929273
GO:0006430	lysyl-tRNA aminoacylation	1	0.70874432	-0.1495104	-0.1495104	0.74584543	0.6926182	0.63283839	0.77183205	0.71873926	0.69888837
GO:0009086	methionine biosynthetic process	5	0.93337907	-0.0299419	-0.1497097	0.95325921	0.92530499	0.8507213	1.01146069	0.9391781	0.92761585
GO:0006605	protein targeting	4	0.91694242	-0.0376579	-0.1506317	0.98399779	0.89432848	0.83315302	0.96416707	0.93809235	0.89626933
GO:0006353	transcription termination%2C DNA-dependent	3	0.89008382	-0.0505691	-0.1517073	1.02666549	0.83756696	0.791486	0.92221439	0.92730852	0.85435342
GO:0009584	detection of visible light	3	0.88751075	-0.0518264	-0.1554791	0.91087591	0.87650968	0.81458041	0.9539917	0.89352759	0.88153443
GO:0009585	red%2C far-red light phototransduction	3	0.88751075	-0.0518264	-0.1554791	0.91087591	0.87650968	0.81458041	0.9539917	0.89352759	0.88153443
GO:0017006	protein-tetrapyrrole linkage	3	0.88751075	-0.0518264	-0.1554791	0.91087591	0.87650968	0.81458041	0.9539917	0.89352759	0.88153443
GO:0018298	protein-chromophore linkage	3	0.88751075	-0.0518264	-0.1554791	0.91087591	0.87650968	0.81458041	0.9539917	0.89352759	0.88153443
GO:0009102	biotin biosynthetic process	1	0.69726733	-0.1566007	-0.1566007	0.73832326	0.67532289	0.62438775	0.75925012	0.70612081	0.68852486
GO:0051186	cofactor metabolic process	1	0.69726733	-0.1566007	-0.1566007	0.73832326	0.67532289	0.62438775	0.75925012	0.70612081	0.68852486
GO:0009052	pentose-phosphate shunt%2C non-oxidative branch	3	0.88666663	-0.0522396	-0.1567189	0.89069771	0.844915	0.82145168	0.99980681	0.86750438	0.90625216
GO:0009312	oligosaccharide biosynthetic process	1	0.69603845	-0.1573668	-0.1573668	0.7237913	0.67858158	0.62258342	0.76757347	0.70082198	0.69128758
GO:0016480	negative regulation of transcription from RNA polym	1	0.69259198	-0.1595225	-0.1595225	0.71213523	0.67520851	0.6280962	0.76187425	0.69342611	0.69175886
GO:0006106	fumarate metabolic process	1	0.69042516	-0.1608834	-0.1608834	0.72982944	0.65211194	0.62361961	0.76560214	0.68987716	0.6909736
GO:0000918	barrier septum site selection	1	0.68958738	-0.1614107	-0.1614107	0.73054392	0.65957664	0.59439107	0.78953854	0.69415395	0.68505085
GO:0009785	blue light signaling pathway	2	0.82758029	-0.0821899	-0.1643797	0.87093563	0.80712168	0.73487381	0.90803529	0.83842175	0.81687903
GO:0033014	tetrapyrrole biosynthetic process	5	0.92657712	-0.0331184	-0.1655921	0.95836218	0.90267465	0.84437016	1.00909601	0.93010174	0.92306585
GO:0018279	protein N-linked glycosylation via asparagine	2	0.82521688	-0.0834319	-0.1668638	0.86006522	0.80225197	0.75627091	0.8886952	0.83065578	0.81981359
GO:0017183	peptidyl-diphthamide biosynthetic process from pep	3	0.87967248	-0.055679	-0.167037	1.01200186	0.81310188	0.77883731	0.93435302	0.90711665	0.85305861
GO:0016925	protein sumoylation	1	0.67979997	-0.1676189	-0.1676189	0.70397845	0.64913137	0.60556755	0.7717382	0.67599889	0.68362242
GO:0031668	cellular response to extracellular stimulus	1	0.67979997	-0.1676189	-0.1676189	0.70397845	0.64913137	0.60556755	0.7717382	0.67599889	0.68362242
GO:0051276	chromosome organization	2	0.82356616	-0.0843015	-0.168603	0.8732687	0.81443279	0.695309	0.93027847	0.84333781	0.80425804
GO:0019432	triglyceride biosynthetic process	2	0.82105332	-0.0856286	-0.1712573	0.8413362	0.80061708	0.71948877	0.93770664	0.82072415	0.82138261
GO:0006544	glycine metabolic process	5	0.92385065	-0.0343982	-0.1719912	0.96842697	0.90280255	0.83039696	1.00337128	0.93503922	0.91279596
GO:0006563	L-serine metabolic process	5	0.92385065	-0.0343982	-0.1719912	0.96842697	0.90280255	0.83039696	1.00337128	0.93503922	0.91279596
GO:0019264	glycine biosynthetic process from serine	1	0.66494417	-0.1772148	-0.1772148	0.66618883	0.64742916	0.60690407	0.74684601	0.65674202	0.67324875
GO:0035999	tetrahydrofolate interconversion	1	0.66494417	-0.1772148	-0.1772148	0.66618883	0.64742916	0.60690407	0.74684601	0.65674202	0.67324875
GO:0045116	protein neddylation	1	0.66079536	-0.179933	-0.179933	0.71927388	0.6343918	0.59297924	0.70465511	0.67550089	0.64640997
GO:0006525	arginine metabolic process	2	0.80869906	-0.0922131	-0.1844261	0.84200342	0.77437558	0.72147476	0.90920349	0.80748182	0.80991812
GO:0045005	maintenance of fidelity involved in DNA-dependent	1	0.65338075	-0.1848337	-0.1848337	0.70419625	0.61428428	0.59674716	0.70601166	0.65770562	0.64908432
GO:0007155	cell adhesion	4	0.89580808	-0.047785	-0.1911401	1.032385	0.85235935	0.76537317	0.9561417	0.93806344	0.85545613
GO:0042255	ribosome assembly	1	0.64383042	-0.1912285	-0.1912285	0.68226516	0.61469535	0.57061235	0.71801197	0.64759958	0.6400832
GO:0006515	protein quality control for misfolded or incompletely	1	0.63946083	-0.1941861	-0.1941861	0.63058874	0.62772442	0.58545918	0.7215126	0.62915495	0.64993552
GO:0006571	tyrosine biosynthetic process	2	0.79808866	-0.0979489	-0.1958977	0.89164607	0.75163219	0.68324718	0.88599005	0.81865126	0.77804255
GO:0046654	tetrahydrofolate biosynthetic process	1	0.63594293	-0.1965819	-0.1965819	0.65209186	0.60739589	0.5816585	0.70994362	0.62934721	0.64260777
GO:0050482	arachidonic acid secretion	2	0.79744124	-0.0983013	-0.1966026	0.82074022	0.79061028	0.72440705	0.86028812	0.80553439	0.78942941
GO:0019856	pyrimidine base biosynthetic process	1	0.63236578	-0.1990316	-0.1990316	0.68160244	0.59754495	0.55736889	0.70441565	0.63819127	0.62659347
GO:0045892	negative regulation of transcription%2C DNA-depen	6	0.92575222	-0.0335052	-0.2010314	0.92697844	0.84516163	0.89372519	1.04897622	0.8851252	0.96824401
GO:0007020	microtubule nucleation	1	0.62839195	-0.2017694	-0.2017694	0.64314752	0.62469809	0.54406407	0.71333187	0.63385568	0.62297531
GO:0008272	sulfate transport	9	0.94941248	-0.0225451	-0.2029056	0.99339236	0.91494769	0.91454558	0.97745619	0.95336355	0.94547778
GO:0019346	transsulfuration	1	0.6205648	-0.2072129	-0.2072129	0.66934303	0.59629836	0.5603245	0.66312677	0.6317659	0.60956228
GO:0009738	abscisic acid-activated signaling pathway	2	0.78371086	-0.1058441	-0.2116883	0.88726559	0.70911101	0.71186033	0.84228836	0.79320224	0.77433305
GO:0032065	cortical protein anchoring	2	0.78347934	-0.1059725	-0.2119449	0.81249037	0.75476505	0.72798227	0.84403292	0.783096	0.78386287
GO:0046417	chorismate metabolic process	4	0.88281126	-0.0541321	-0.2165285	0.95450847	0.86560642	0.81595094	0.90096341	0.90897121	0.85740419
GO:0015770	sucrose transport	3	0.84687867	-0.0721788	-0.2165364	0.89231199	0.81404147	0.78831598	0.89829957	0.85227869	0.84151286
GO:0035246	peptidyl-arginine N-methylation	1	0.60679012	-0.2169615	-0.2169615	0.63717397	0.57463031	0.55749923	0.6641454	0.6050946	0.60849039
GO:0006425	glutaminyl-tRNA aminoacylation	2	0.77767883	-0.1091997	-0.2183994	0.83312794	0.74441075	0.69331855	0.85063638	0.78752105	0.76795962
GO:0008380	RNA splicing	2	0.77756713	-0.1092621	-0.2185242	0.8232162	0.7453372	0.7009511	0.84995735	0.78330943	0.77186692
GO:0044249	cellular biosynthetic process	3	0.84544905	-0.0729126	-0.2187377	0.91870995	0.81181403	0.74794093	0.91589878	0.86360965	0.82767034
GO:0006422	aspartyl-tRNA aminoacylation	2	0.77720486	-0.1094645	-0.218929	0.80625391	0.76400552	0.68843613	0.86041912	0.78484549	0.76963862
GO:1901642	nucleoside transmembrane transport	4	0.88143699	-0.0548087	-0.2192349	0.94179644	0.85706059	0.80583069	0.92801011	0.89843009	0.8647653
GO:0030833	regulation of actin filament polymerization	7	0.92984136	-0.0315911	-0.221138	1.02624373	0.88048828	0.81155233	1.01940041	0.95057645	0.90955856
GO:0018160	peptidyl-pyrromethane cofactor linkage	1	0.59694611	-0.2240649	-0.2240649	0.61683165	0.5859212	0.53999165	0.65065024	0.6011778	0.59274421
GO:0071722	detoxification of arsenic-containing substance	3	0.84169903	-0.0748432	-0.2245295	0.9229091	0.79863504	0.75157712	0.9060381	0.85852638	0.82520149
GO:0000917	barrier septum formation	2	0.77136689	-0.112739	-0.225478	0.77278559	0.81315744	0.71184438	0.79145325	0.79271454	0.75059413
GO:0071951	conversion of methionyl-tRNA to N-formyl-methiony	1	0.5941329	-0.2261164	-0.2261164	0.68909364	0.54167264	0.55270351	0.60398624	0.61095268	0.57777618
GO:0016114	terpenoid biosynthetic process	6	0.91606632	-0.0380731	-0.2284385	0.9736842	0.88389088	0.81824456	1.00001788	0.92770178	0.9045768
GO:0006637	acyl-CoA metabolic process	1	0.58738853	-0.2310745	-0.2310745	0.60490768	0.56501534	0.54217198	0.64241463	0.58462135	0.5901688
GO:0009452	RNA capping	1	0.58380161	-0.2337347	-0.2337347	0.64698763	0.53815249	0.46013161	0.72506634	0.5900661	0.57760362
GO:0016458	gene silencing	1	0.58345536	-0.2339924	-0.2339924	0.59270642	0.55898217	0.5458093	0.64084357	0.57559736	0.59142064
GO:0042558	pteridine-containing compound metabolic process	1	0.5800343	-0.2365463	-0.2365463	0.7710176	0.46266064	0.49639762	0.6392315	0.59725999	0.56330542
GO:0006820	anion transport	12	0.9550851	-0.0199579	-0.2394951	0.99349809	0.93338519	0.8774138	1.02267087	0.96297269	0.94726212
GO:0019556	histidine catabolic process to glutamate and formam	1	0.57528873	-0.2401141	-0.2401141	0.54815856	0.60020008	0.554096	0.60083637	0.57358941	0.57699309
GO:0006207	'de novo' pyrimidine base biosynthetic process	4	0.87009387	-0.0604339	-0.2417356	0.91591602	0.83947491	0.78235552	0.95278939	0.87686289	0.86337711
GO:0016579	protein deubiquitination	5	0.8939788	-0.0486728	-0.2433639	0.95709619	0.864543	0.79534269	0.9705377	0.90964323	0.87858412
GO:0015696	ammonium transport	4	0.8691612	-0.0608997	-0.2435987	0.92114154	0.93605909	0.80589098	0.82128786	0.92857036	0.81355299
GO:0006002	fructose 6-phosphate metabolic process	1	0.56891366	-0.2449536	-0.2449536	0.59508823	0.53779799	0.50622679	0.64660608	0.56571835	0.57212702
GO:0006884	cell volume homeostasis	2	0.75358764	-0.1228662	-0.2457325	0.83662439	0.72150622	0.68083439	0.7847349	0.7769361	0.73094084
GO:0007034	vacuolar transport	3	0.82728772	-0.0823434	-0.2470303	0.89394152	0.78350815	0.72015119	0.92864558	0.83690529	0.81778067
GO:0006366	transcription from RNA polymerase II promoter	4	0.86673743	-0.0621124	-0.2484498	0.97055622	0.8291227	0.76541697	0.91624712	0.89705641	0.83744319
GO:0007015	actin filament organization	2	0.74924956	-0.1253735	-0.250747	0.79791416	0.71529985	0.68418595	0.807026	0.75547858	0.7430719
GO:0006426	glycyl-tRNA aminoacylation	3	0.82472859	-0.0836889	-0.2510668	0.87028167	0.78709057	0.74702874	0.90411224	0.82764153	0.82182591
GO:0006428	isoleucyl-tRNA aminoacylation	1	0.5573156	-0.2538988	-0.2538988	0.60567627	0.53666609	0.49419124	0.60057195	0.57012798	0.54479115
GO:0030071	regulation of mitotic metaphase/anaphase transitio	3	0.82238101	-0.0849269	-0.2547808	0.89399311	0.81781464	0.7139518	0.87626273	0.85505594	0.79095471
GO:0006421	asparaginyl-tRNA aminoacylation	3	0.82067286	-0.0858299	-0.2574898	0.8515133	0.80062685	0.75248649	0.88421932	0.82567815	0.81569791
GO:0006000	fructose metabolic process	2	0.74296339	-0.1290326	-0.2580652	0.79125811	0.71439587	0.65431765	0.82380405	0.75184541	0.7341863
GO:0006003	fructose 2%2C6-bisphosphate metabolic process	2	0.74296339	-0.1290326	-0.2580652	0.79125811	0.71439587	0.65431765	0.82380405	0.75184541	0.7341863

GO:0006222	UMP biosynthetic process	1	0.54674695	-0.2622136	-0.2622136	0.59097561	0.53183785	0.48746358	0.58324968	0.56062751	0.53321007
GO:0071266	'de novo' L-methionine biosynthetic process	2	0.73659121	-0.1327735	-0.2655469	0.78914032	0.72448602	0.65002789	0.79211802	0.75612243	0.7175645
GO:0043066	negative regulation of apoptosis	1	0.53692479	-0.2700865	-0.2700865	0.55839195	0.51046117	0.48171013	0.60529373	0.53388895	0.53997789
GO:0006370	mRNA capping	3	0.81269095	-0.0900746	-0.2702237	0.87063978	0.77262101	0.73490316	0.88240215	0.82016741	0.80528264
GO:0006573	valine metabolic process	2	0.72990835	-0.1367317	-0.2734633	0.76342863	0.70927954	0.65230852	0.80358978	0.73585617	0.7240086
GO:0006429	leucyl-tRNA aminoacylation	2	0.72928072	-0.1371053	-0.2742105	0.78429471	0.68141435	0.66834514	0.79193143	0.73104697	0.72751874
GO:0006081	cellular aldehyde metabolic process	5	0.88112786	-0.0549611	-0.2748053	0.88114957	0.97739587	0.80085662	0.87393841	0.92802583	0.83659988
GO:0006278	RNA-dependent DNA replication	7	0.91344071	-0.0393196	-0.2752374	0.96900152	0.90097249	0.83558443	0.95432244	0.93436808	0.89298206
GO:0030259	lipid glycosylation	6	0.89846632	-0.0464982	-0.2789892	0.92627721	0.88427483	0.80066102	0.99364278	0.90503238	0.89194789
GO:0006165	nucleoside diphosphate phosphorylation	5	0.87786105	-0.0565742	-0.2828711	0.91473352	0.84688375	0.79255939	0.96728156	0.88015507	0.875573
GO:0006183	GTP biosynthetic process	5	0.87786105	-0.0565742	-0.2828711	0.91473352	0.84688375	0.79255939	0.96728156	0.88015507	0.875573
GO:0006228	UTP biosynthetic process	5	0.87786105	-0.0565742	-0.2828711	0.91473352	0.84688375	0.79255939	0.96728156	0.88015507	0.875573
GO:0009239	enterobactin biosynthetic process	2	0.71948888	-0.1429759	-0.2859518	0.89808979	0.72843559	0.57190797	0.71624117	0.80882666	0.64001878
GO:0006487	protein N-linked glycosylation	5	0.87655811	-0.0572193	-0.2860964	0.92291532	0.84302825	0.78551801	0.96596798	0.88206785	0.8710828
GO:0006811	ion transport	20	0.96719086	-0.0144878	-0.2897563	1.01387372	0.92880693	0.93388375	0.99505389	0.97040865	0.96398374
GO:0006783	heme biosynthetic process	3	0.79774687	-0.0981349	-0.2944047	0.84684136	0.77214495	0.71410388	0.86735753	0.80863111	0.78700913
GO:0009742	brassinosteroid mediated signaling pathway	2	0.71027805	-0.1485716	-0.2971432	0.78783338	0.66874682	0.6384161	0.75668243	0.72585196	0.6950383
GO:0006402	mRNA catabolic process	2	0.70822594	-0.1498282	-0.2996563	0.73221787	0.69735997	0.62446373	0.78901046	0.7145764	0.70193192
GO:0015937	coenzyme A biosynthetic process	3	0.79419248	-0.1000742	-0.3002227	0.85535498	0.76469715	0.70831197	0.85870233	0.80875677	0.77989046
GO:0046836	glycolipid transport	6	0.89062935	-0.050303	-0.301818	0.92477435	0.87113948	0.82027124	0.9521537	0.89755638	0.88375579
GO:0009408	response to heat	10	0.93284579	-0.0301901	-0.3019014	0.97413496	0.90118367	0.84728435	1.01807036	0.93694958	0.92875997
GO:0008654	phospholipid biosynthetic process	11	0.93831855	-0.0276497	-0.3041467	0.95083391	1.04924987	0.80055318	0.97057127	0.9988305	0.88147258
GO:0009234	menaquinone biosynthetic process	2	0.69863601	-0.155749	-0.3114981	0.72695557	0.67705172	0.62864042	0.76996655	0.70156007	0.69572415
GO:0016485	protein processing	4	0.83447517	-0.0785866	-0.3143463	0.87347605	0.80271631	0.75429977	0.91684644	0.83734908	0.83161112
GO:0006790	sulfur compound metabolic process	1	0.48180919	-0.3171249	-0.3171249	0.56601527	0.44881889	0.40986312	0.51756176	0.50402217	0.46057516
GO:0030036	actin cytoskeleton organization	16	0.9538638	-0.0205136	-0.3282182	0.98926522	0.9335784	0.87830993	1.02054964	0.96101854	0.94676232
GO:0006452	translational frameshifting	4	0.82561309	-0.0832234	-0.3328937	0.87234675	0.79644578	0.74325399	0.89975375	0.83353278	0.81776865
GO:0045901	positive regulation of translational elongation	4	0.82561309	-0.0832234	-0.3328937	0.87234675	0.79644578	0.74325399	0.89975375	0.83353278	0.81776865
GO:0045905	positive regulation of translational termination	4	0.82561309	-0.0832234	-0.3328937	0.87234675	0.79644578	0.74325399	0.89975375	0.83353278	0.81776865
GO:0046949	fatty-acyl-CoA biosynthetic process	1	0.4645389	-0.3329779	-0.3329779	0.47739502	0.46302357	0.42620999	0.49429219	0.47015439	0.45899049
GO:0042176	regulation of protein catabolic process	6	0.87962199	-0.0557039	-0.3342235	0.92427579	0.85088053	0.79106042	0.96228671	0.88681919	0.8724832
GO:0006879	cellular iron ion homeostasis	4	0.82461385	-0.0837494	-0.3349975	0.91255958	0.7805096	0.75524846	0.859554	0.84395587	0.80571511
GO:0043666	regulation of phosphoprotein phosphatase activity	2	0.67906804	-0.1680867	-0.3361734	0.72556949	0.65749275	0.60735289	0.73390878	0.6906929	0.66763884
GO:0009733	response to auxin	4	0.82290026	-0.0846528	-0.3386112	1.10312764	0.77672985	0.68527747	0.78095605	0.92565229	0.73155423
GO:0019419	sulfate reduction	1	0.45438741	-0.3425737	-0.3425737	0.47933683	0.43951475	0.42750427	0.4733152	0.45899413	0.44982693
GO:0000226	microtubule cytoskeleton organization	7	0.89163408	-0.0498133	-0.3486934	0.91042349	0.88431774	0.81577189	0.96233463	0.89727568	0.88602795
GO:0019509	L-methionine salvage from methylthioadenosine	4	0.81556608	-0.0885408	-0.3541634	0.88353185	0.76962582	0.72095104	0.90246218	0.82461441	0.80661704
GO:0034755	iron ion transmembrane transport	2	0.66506686	-0.1771347	-0.3542694	0.71890478	0.63729276	0.59458529	0.71818565	0.67686986	0.65346968
GO:0016070	RNA metabolic process	14	0.94330793	-0.0253465	-0.3548512	0.99375917	0.91980817	0.85837259	1.00915921	0.95606893	0.93071725
GO:0006537	glutamate biosynthetic process	3	0.76132211	-0.1184316	-0.3552947	0.80729463	0.73848296	0.66895699	0.84237026	0.77212261	0.75067268
GO:0006013	mannose metabolic process	3	0.75979809	-0.1193018	-0.3579054	0.78467684	0.73650525	0.69565563	0.82895662	0.76020959	0.75938682
GO:0006424	glutamyl-tRNA aminoacylation	2	0.66198882	-0.1791493	-0.3582987	0.74486736	0.62257245	0.58131146	0.71240161	0.6809801	0.64352717
GO:0046939	nucleotide phosphorylation	7	0.8864181	-0.0523614	-0.3665297	0.95943655	0.86574004	0.77302153	0.96152176	0.91138501	0.86213515
GO:0006813	potassium ion transport	19	0.95624179	-0.0194323	-0.3692133	1.02169174	0.93392658	0.86217144	1.01635315	0.97682397	0.93609329
GO:0019307	mannose biosynthetic process	1	0.4259449	-0.3706466	-0.3706466	0.45341554	0.40252224	0.38626491	0.46691965	0.4272117	0.42468185
GO:0046835	carbohydrate phosphorylation	3	0.75130477	-0.1241839	-0.3725516	0.78120258	0.73083574	0.69073091	0.80792725	0.7555996	0.74703435
GO:0017003	protein-heme linkage	1	0.42389319	-0.3727436	-0.3727436	0.44971936	0.3955599	0.38251539	0.47448556	0.4217712	0.42602585
GO:0019358	nicotinate nucleotide salvage	2	0.64655455	-0.1893948	-0.3787897	0.67005799	0.62666767	0.57638964	0.72202934	0.64799975	0.64511257
GO:0043044	ATP-dependent chromatin remodeling	2	0.64378253	-0.1912608	-0.3825216	0.70344368	0.61585119	0.57720381	0.68694575	0.65819194	0.62968858
GO:0043161	proteasomal ubiquitin-dependent protein catabolic p	3	0.74368265	-0.1286124	-0.3858371	0.78863461	0.71678481	0.67176382	0.80550708	0.75185192	0.73560214
GO:0000105	histidine biosynthetic process	7	0.88078456	-0.0551303	-0.3859122	0.90878329	0.85766724	0.79365533	0.97289881	0.8828554	0.87871857
GO:0006241	CTP biosynthetic process	6	0.86005962	-0.0654714	-0.3928287	0.89065798	0.83222955	0.77892225	0.94768941	0.86094825	0.85917191
GO:0048193	Golgi vesicle transport	7	0.8779777	-0.0565165	-0.3956156	0.9503999	0.85253938	0.76791328	0.95499464	0.90014073	0.85636036
GO:0006825	copper ion transport	3	0.73727201	-0.1323723	-0.3971168	0.78059671	0.72130337	0.67978123	0.77196417	0.7503646	0.72440786
GO:0006541	glutamine metabolic process	4	0.79413123	-0.1001077	-0.4004309	0.88460819	0.80801123	0.7223871	0.77024833	0.8454427	0.74593395
GO:0008652	cellular amino acid biosynthetic process	20	0.95487455	-0.0200537	-0.4010736	1.01190586	0.93145593	0.85638325	1.02994657	0.97084793	0.93916398
GO:0006529	asparagine biosynthetic process	3	0.73499672	-0.1337146	-0.4011438	0.81834244	0.76058746	0.70461649	0.66543337	0.78893663	0.68474471
GO:0006437	tyrosyl-tRNA aminoacylation	1	0.39614713	-0.4021435	-0.4021435	0.29400963	0.45993879	0.3977051	0.45793445	0.36773147	0.42675856
GO:0006760	folic acid-containing compound metabolic process	3	0.73219027	-0.135376	-0.4061281	0.73212251	0.71325886	0.68671768	0.80146885	0.72262914	0.74187791
GO:0006388	tRNA splicing%2C via endonucleolytic cleavage and	2	0.61992653	-0.2076598	-0.4153196	0.65791124	0.61047361	0.54843454	0.67050481	0.63374873	0.6064058
GO:0006772	thiamine metabolic process	3	0.72359971	-0.1405016	-0.4215049	0.78400174	0.7030746	0.64521954	0.77084592	0.74243633	0.70524099
GO:0009229	thiamine diphosphate biosynthetic process	3	0.72359971	-0.1405016	-0.4215049	0.78400174	0.7030746	0.64521954	0.77084592	0.74243633	0.70524099
GO:0019538	protein metabolic process	17	0.94429283	-0.0248933	-0.4231863	1.0052475	0.89554584	0.87821309	1.0056944	0.94881253	0.93979465
GO:0006164	purine nucleotide biosynthetic process	5	0.82217798	-0.0850342	-0.4251708	0.87592572	0.83100994	0.76129048	0.82459265	0.85317231	0.79230962
GO:0002098	tRNA wobble uridine modification	3	0.71597675	-0.1451011	-0.4353032	0.7425578	0.68837707	0.64419981	0.79802853	0.71495438	0.71700058
GO:0016571	histone methylation	3	0.71594057	-0.145123	-0.4353691	0.7888837	0.68806533	0.64251815	0.75332058	0.73675201	0.695717
GO:0031120	snRNA pseudouridine synthesis	2	0.60549488	-0.2178895	-0.435779	0.63509623	0.58785408	0.5674962	0.6344098	0.61101874	0.60002096
GO:0032957	inositol trisphosphate metabolic process	6	0.84291916	-0.0742141	-0.4452844	0.8802352	0.82699868	0.75723454	0.9158192	0.85320182	0.83276043
GO:0019725	cellular homeostasis	1	0.35856878	-0.4454275	-0.4454275	0.34463746	0.32428394	0.262097	0.5643388	0.33430584	0.38459265
GO:0032012	regulation of ARF protein signal transduction	4	0.77347211	-0.1115553	-0.4462214	0.82677963	0.74761033	0.70425076	0.82221662	0.78619908	0.76095117
GO:0016570	histone modification	3	0.70642055	-0.1509367	-0.45281	0.72527169	0.69763494	0.65530855	0.75106673	0.71131911	0.70155573
GO:0008283	cell proliferation	6	0.83996828	-0.0757371	-0.4544227	0.79182989	0.74853248	0.83660019	1.00390134	0.76987687	0.91644097
GO:0007031	peroxisome organization	5	0.80992094	-0.0915574	-0.4577869	0.85947601	0.78828598	0.70540079	0.90036167	0.82311171	0.79694155
GO:0006564	L-serine biosynthetic process	6	0.83728903	-0.0771246	-0.4627476	0.89352427	0.79986147	0.76112779	0.90348907	0.84539674	0.82925909
GO:0006535	cysteine biosynthetic process from serine	16	0.93526355	-0.029066	-0.4650558	1.01519964	0.8650736	0.90554369	0.96210422	0.93713521	0.93339563
GO:0006427	histidyl-tRNA aminoacylation	2	0.58511798	-0.2327566	-0.4655131	0.63012071	0.56421656	0.5289327	0.62330954	0.59625878	0.57418533
GO:0006400	tRNA modification	7	0.85602204	-0.0675151	-0.4726054	0.91339045	0.83068171	0.75833724	0.93322484	0.87105495	0.84124856
GO:0005985	sucrose metabolic process	7	0.85517487	-0.0679451	-0.4756155	0.93611807	0.83416478	0.74341689	0.92130797	0.88367229	0.82759646
GO:0019363	pyridine nucleotide biosynthetic process	2	0.573521	-0.2414507	-0.4829014	0.62844643	0.54883287	0.50222311	0.62458597	0.58729214	0.56007277
GO:0015992	proton transport	14	0.92322073	-0.0346945	-0.4857224	0.96839913	0.89723351	0.83823495	0.99746241	0.93213741	0.91438934
GO:0007154	cell communication	6	0.82649463	-0.08276	-0.4965598	0.88684143	0.79435199	0.73166289	0.90529507	0.83932369	0.81386166
GO:0016559	peroxisome fission	4	0.74414177	-0.1283443	-0.5133773	0.8103836	0.71252363	0.66494417	0.79863369	0.7598799	0.72872959
GO:0009308	amine metabolic process	10	0.88709794	-0.0520284	-0.5202843	0.9504457	0.8620682	0.80247191	0.94186253	0.90517899	0.86937807
GO:0045893	positive regulation of transcription%2C DNA-depend	7	0.84094095	-0.0752345	-0.5266415	0.9605198	0.785888	0.75257453	0.88033003	0.86882736	0.8139496
GO:0032313	regulation of Rab GTPase activity	14	0.91648512	-0.0378746	-0.5302442	0.96927303	0.8847586	0.82869785	0.99273774	0.92605219	0.90701688

GO:0007585 respiratory gaseous exchange	5	0.78321504	-0.106119	-0.5305949	0.81331681	0.75115005	0.71766601	0.85825285	0.78161561	0.78481775
GO:0006189 'de novo' IMP biosynthetic process	6	0.81462787	-0.0890407	-0.5342444	0.88462764	0.77213231	0.72131221	0.8938436	0.82646814	0.80295723
GO:0030244 cellulose biosynthetic process	29	0.95844601	-0.0184323	-0.5345381	1.01302582	0.93474502	0.88855233	1.00293761	0.97309858	0.94401406
GO:0009113 purine base biosynthetic process	4	0.73176983	-0.1356255	-0.542502	0.75974992	0.70787529	0.65401914	0.81522994	0.73335408	0.73018901
GO:0034227 tRNA thio-modification	4	0.73166302	-0.1356889	-0.5427556	0.79092383	0.70292346	0.63977438	0.80570283	0.74562652	0.71796102
GO:0006168 adenine salvage	4	0.73091445	-0.1361335	-0.5445338	0.78933776	0.71918622	0.66757137	0.75312011	0.75344598	0.70905672
GO:0032259 methylation	14	0.91291209	-0.039571	-0.5539946	0.9978678	0.89564732	0.81046835	0.95889209	0.94537697	0.88156207
GO:0006807 nitrogen compound metabolic process	18	0.93118562	-0.0309637	-0.5573473	0.99031027	0.90729778	0.8355495	1.00150179	0.94789573	0.91477009
GO:0032968 positive regulation of transcription elongation from F	2	0.52600407	-0.2790109	-0.5580218	0.53006419	0.52436307	0.46398359	0.5935991	0.52720592	0.52480496
GO:0048268 clathrin coat assembly	10	0.87848984	-0.0562633	-0.5626326	0.93239811	0.85817174	0.79977257	0.93068961	0.89451535	0.86275142
GO:0070588 calcium ion transmembrane transport	10	0.8781826	-0.0564152	-0.5641517	0.91867064	0.86143568	0.79523723	0.94506114	0.88959298	0.86691857
GO:0009396 folic acid-containing compound biosynthetic proces	11	0.88855168	-0.0513173	-0.5644904	0.96095624	0.84838211	0.79211927	0.9652617	0.90291643	0.87441546
GO:0006526 arginine biosynthetic process	7	0.83042793	-0.0806981	-0.5648864	0.88608743	0.81119307	0.74638054	0.88643456	0.84781365	0.81339874
GO:0006075 (1->3)-beta-D-glucan biosynthetic process	9	0.86399643	-0.0634881	-0.5713925	0.89544026	0.84694647	0.77675944	0.94595106	0.87085588	0.857191
GO:0016226 iron-sulfur cluster assembly	15	0.91475661	-0.0386944	-0.5804167	0.97316553	0.86833656	0.81473373	1.01702522	0.91925797	0.91027729
GO:0006432 phenylalanyl-tRNA aminoacylation	3	0.63954246	-0.1941306	-0.5823919	0.65751679	0.63949063	0.57007991	0.69791216	0.64844107	0.63076596
GO:0016998 cell wall macromolecule catabolic process	22	0.94080814	-0.0264989	-0.5829766	0.97807525	0.92746369	0.82805949	1.04297413	0.95243335	0.92932482
GO:0009168 purine ribonucleoside monophosphate biosynthetic	3	0.63466674	-0.1974543	-0.5923628	0.65624049	0.63347003	0.56772034	0.68747897	0.64475474	0.62473658
GO:0001510 RNA methylation	5	0.76051231	-0.1188938	-0.5944688	0.81628043	0.7247176	0.66162435	0.8546835	0.76913769	0.75198365
GO:0006338 chromatin remodeling	3	0.62800427	-0.2020374	-0.6061122	0.66761799	0.60401828	0.56652903	0.68084789	0.63502242	0.62106368
GO:0009059 macromolecule biosynthetic process	3	0.62036548	-0.2073524	-0.6220571	0.61175946	0.66510963	0.57490068	0.63317497	0.63787703	0.60333467
GO:0006415 translational termination	10	0.86333115	-0.0638226	-0.6382259	1.11251178	0.77622692	0.76307583	0.84304086	0.92928015	0.80206241
GO:0006730 one-carbon metabolic process	11	0.87488101	-0.058051	-0.6385611	0.82988161	0.97545138	0.75100172	0.96368092	0.89972727	0.85072089
GO:0006631 fatty acid metabolic process	14	0.89848773	-0.0464878	-0.6508299	0.93902026	0.87190493	0.80356049	0.99057175	0.90484053	0.89217953
GO:0031123 RNA 3'-end processing	4	0.68619655	-0.1635515	-0.6542059	0.73585405	0.68588677	0.60335401	0.72807785	0.71043125	0.66278857
GO:0043631 RNA polyadenylation	4	0.68619655	-0.1635515	-0.6542059	0.73585405	0.68588677	0.60335401	0.72807785	0.71043125	0.66278857
GO:0009072 aromatic amino acid family metabolic process	6	0.77729781	-0.1094126	-0.6564753	0.83018048	0.74373093	0.70419103	0.83959708	0.78576771	0.7689192
GO:0006812 cation transport	80	0.98125298	-0.008219	-0.6575208	1.01620456	0.99597385	0.89479101	1.02370135	1.00603835	0.95707824
GO:0006420 arginyl-tRNA aminoacylation	2	0.46473299	-0.3327965	-0.665593	0.48263199	0.45508546	0.41234106	0.5150488	0.46865638	0.46084246
GO:0006434 seryl-tRNA aminoacylation	3	0.59202047	-0.2276633	-0.6829898	0.63018535	0.5766916	0.52274552	0.64661331	0.60284542	0.58138989
GO:0006505 GPI anchor metabolic process	3	0.58624433	-0.2319213	-0.695764	0.64116168	0.55450415	0.50035407	0.66399503	0.59626069	0.57639623
GO:0006308 DNA catabolic process	4	0.66172311	-0.1793237	-0.7172948	0.69414903	0.65013124	0.61755918	0.68797532	0.67177971	0.65181706
GO:0006071 glycerol metabolic process	13	0.87924265	-0.0558913	-0.7265863	0.90856874	0.86537489	0.8106512	0.93764592	0.88670884	0.87183932
GO:0016575 histone deacetylation	6	0.75664693	-0.1211067	-0.7266404	0.80909969	0.73508643	0.67647635	0.81466682	0.77120568	0.74236301
GO:0006090 pyruvate metabolic process	5	0.71307291	-0.1468661	-0.7343303	0.75299678	0.68229068	0.63486848	0.79266454	0.71677241	0.70939251
GO:0008643 carbohydrate transport	7	0.78511762	-0.1050653	-0.7354569	0.79914128	0.765921	0.7402956	0.83854461	0.78235484	0.78789015
GO:0072488 ammonium transmembrane transport	9	0.82774499	-0.0821034	-0.738931	0.89181559	0.82181691	0.76660771	0.83553169	0.85610112	0.80032808
GO:0006419 alanyl-tRNA aminoacylation	3	0.5624077	-0.2499487	-0.7498462	0.58931686	0.54706225	0.51154698	0.60664399	0.56779662	0.55706992
GO:0006431 methionyl-tRNA aminoacylation	2	0.42037312	-0.3763651	-0.7527301	0.48359336	0.41663302	0.37658656	0.41156736	0.44886631	0.39368863
GO:0016075 rRNA catabolic process	10	0.83428127	-0.0786875	-0.7868751	0.86128011	0.81611077	0.76617958	0.89955118	0.8383913	0.83019139
GO:0006904 vesicle docking involved in exocytosis	11	0.8472587	-0.071984	-0.7918236	0.9201132	0.80439101	0.76272742	0.91282245	0.86030854	0.8344068
GO:0042026 protein refolding	8	0.79163782	-0.1014735	-0.8117877	0.81434702	0.77533938	0.71722994	0.86725414	0.79460387	0.78868285
GO:0009098 leucine biosynthetic process	3	0.53441851	-0.2721185	-0.8163555	0.5680129	0.52107801	0.48188495	0.57190207	0.54403955	0.52496762
GO:0006865 amino acid transport	2	0.38989878	-0.4090481	-0.8180962	0.41344441	0.3761632	0.34670228	0.42860515	0.3943635	0.38548461
GO:0015689 molybdate ion transport	3	0.53275884	-0.2734693	-0.820408	0.57222085	0.54052434	0.43464184	0.59925575	0.55614683	0.51035441
GO:0006298 mismatch repair	12	0.85213695	-0.0694906	-0.8338872	0.91788081	0.81741327	0.75656826	0.92888405	0.86619164	0.83831032
GO:0006635 fatty acid beta-oxidation	3	0.52565059	-0.2793028	-0.8379085	0.55262818	0.50923377	0.47553512	0.5705002	0.53048745	0.52085784
GO:0046907 intracellular transport	4	0.60935407	-0.2151303	-0.8605211	0.65641218	0.59050861	0.54982697	0.64691907	0.62258899	0.59640049
GO:0009116 nucleoside metabolic process	18	0.8952781	-0.048042	-0.8647567	0.96119998	0.87156532	0.80291109	0.95510407	0.91528606	0.87570752
GO:0006465 signal peptide processing	10	0.8194366	-0.0864846	-0.8648464	0.89979186	0.80877955	0.72353404	0.85630848	0.85307283	0.78712663
GO:0019310 inositol catabolic process	1	0.13449666	-0.8712885	-0.8712885	0.14886569	0.13272087	0.13606083	0.12172478	0.14056167	0.12869334
GO:0006914 autophagy	5	0.66078221	-0.1799417	-0.8997083	0.68598104	0.64172948	0.59750123	0.72482049	0.66348644	0.658089
GO:0006470 protein dephosphorylation	62	0.96699644	-0.0145751	-0.9036578	1.03086379	0.93464247	0.86417949	1.05014374	0.98157479	0.9526346
GO:0034968 histone lysine methylation	12	0.84047988	-0.0754727	-0.9056721	0.89960593	0.79905647	0.75315556	0.92171111	0.84784193	0.83318176
GO:0071918 urea transmembrane transport	1	0.1223227	-0.9124929	-0.9124929	0.13201397	0.11633434	0.10858864	0.13425052	0.12392642	0.12073972
GO:0006265 DNA topological change	11	0.82476867	-0.0836678	-0.9203463	0.86396466	0.79272261	0.74882479	0.9022589	0.82757738	0.82196948
GO:0042742 defense response to bacterium	4	0.58614845	-0.2319924	-0.9279695	0.62932507	0.5600914	0.5398213	0.62036391	0.59369989	0.57869306
GO:0050832 defense response to fungus	4	0.58614845	-0.2319924	-0.9279695	0.62932507	0.5600914	0.5398213	0.62036391	0.59369989	0.57869306
GO:0006289 nucleotide-excision repair	21	0.90009869	-0.0457099	-0.9599073	0.94404373	0.87070148	0.80942323	0.98655989	0.90663128	0.89361317
GO:0019318 hexose metabolic process	5	0.63996268	-0.1938454	-0.9692268	0.66316758	0.62527555	0.59726244	0.67726502	0.64394291	0.63600704
GO:0006357 regulation of transcription by RNA polymerase II	14	0.84984283	-0.0706614	-0.9892594	0.89691948	0.81498692	0.77034961	0.92632309	0.85497231	0.84474412
GO:0035434 copper ion transmembrane transport	5	0.62951978	-0.2009906	-1.0049531	0.65117877	0.60709911	0.58196513	0.68262268	0.62875278	0.63028771
GO:0005992 trehalose biosynthetic process	22	0.89842419	-0.0465186	-1.0234084	0.96321377	0.85908797	0.81875586	0.96163663	0.90966223	0.88732498
GO:0016117 carotenoid biosynthetic process	5	0.62398702	-0.2048244	-1.0241222	0.6739544	0.59657236	0.56026798	0.67299648	0.63408404	0.6140508
GO:0015914 phospholipid transport	7	0.71126477	-0.1479687	-1.0357809	0.75547703	0.69201228	0.63735076	0.76808913	0.72304867	0.69967292
GO:0006796 phosphate-containing compound metabolic process	11	0.80396914	-0.0947606	-1.0423668	0.8605718	0.77588788	0.71509625	0.87499774	0.81713354	0.79101682
GO:0046470 phosphatidylcholine metabolic process	10	0.78650072	-0.1043009	-1.0430088	0.84132422	0.75012772	0.70716009	0.85739341	0.79441842	0.77866193
GO:0009966 regulation of signal transduction	8	0.73804611	-0.1319165	-1.055332	0.80024944	0.69266342	0.66419787	0.80591431	0.74451563	0.73163281
GO:0035556 intracellular signal transduction	22	0.89387088	-0.0487252	-1.0719546	0.96484427	0.86306643	0.79567336	0.96352479	0.91253751	0.87558609
GO:0044070 regulation of anion transport	8	0.73342429	-0.1346447	-1.0771577	0.76163592	0.71743637	0.66276394	0.79897184	0.73920586	0.72768793
GO:0007205 activation of protein kinase C activity by G-protein c	12	0.81100399	-0.090977	-1.0917241	0.844205	0.78320416	0.72562385	0.90169052	0.81313275	0.8088808
GO:0006438 valyl-tRNA aminoacylation	4	0.52608037	-0.2789479	-1.1157916	0.55707941	0.5032981	0.4834686	0.56506411	0.52950638	0.52267653
GO:0010215 cellulose microfibril organization	7	0.68775569	-0.1625658	-1.1379607	0.74718969	0.67065654	0.58732548	0.7601983	0.70788957	0.66819446
GO:0016049 cell growth	3	0.41631102	-0.3805821	-1.1417463	0.45327831	0.38471688	0.36892314	0.46690613	0.41759288	0.4150331
GO:0006367 transcription initiation from RNA polymerase II prom	14	0.82794334	-0.0819994	-1.1479914	0.88000154	0.80409467	0.73981829	0.89760815	0.84119234	0.81490302
GO:0000413 protein peptidyl-prolyl isomerization	5	0.58477878	-0.2330084	-1.165042	0.6338002	0.54901566	0.53364234	0.62976574	0.58988663	0.57971515
GO:0006397 mRNA processing	16	0.84199117	-0.0746925	-1.1950794	0.89885931	0.80788915	0.75356991	0.91846602	0.85216118	0.83194252
GO:0009435 NAD biosynthetic process	7	0.67256471	-0.1722659	-1.2058615	0.73351232	0.64406933	0.59237839	0.73113415	0.68733746	0.65810946
GO:0016042 lipid catabolic process	8	0.70454533	-0.1520911	-1.2167285	0.77547494	0.69819787	0.62088969	0.73295112	0.73582264	0.67459751
GO:0006012 galactose metabolic process	10	0.74811943	-0.1260291	-1.2602907	0.78470338	0.73119107	0.67053105	0.81419502	0.75747482	0.73887959
GO:0042256 mature ribosome assembly	2	0.23194148	-0.6346216	-1.2692431	0.23559762	0.23107594	0.19867029	0.26758092	0.23332583	0.23056535
GO:0044262 cellular carbohydrate metabolic process	10	0.73944256	-0.1310956	-1.3109556	0.78851833	0.71433366	0.6589302	0.80549983	0.75050995	0.72853838
GO:0006479 protein methylation	8	0.68091694	-0.1669059	-1.3352469	0.7195187	0.65802629	0.6139828	0.73949462	0.68808591	0.67382266
GO:0009247 glycolipid biosynthetic process	3	0.3578292	-0.4463242	-1.3389727	0.37092598	0.36359128	0.30681231	0.3962138	0.36724032	0.34865925
GO:0006073 cellular glucan metabolic process	21	0.86029698	-0.0653516	-1.3723837	0.92834405	0.82721917	0.7686366	0.92798932	0.87632414	0.84456294

GO:0017148	negative regulation of translation	15	0.8006181	-0.0965746	-1.4486189	0.8011318	0.90314375	0.71309055	0.79633553	0.85060989	0.75356442
GO:0006821	chloride transport	11	0.73310814	-0.134832	-1.4831515	0.7924754	0.70002992	0.65758214	0.79180777	0.74481977	0.72158066
GO:0016311	dephosphorylation	22	0.85466709	-0.068203	-1.5004664	0.90121713	0.83600974	0.76600203	0.92452196	0.86800133	0.8415377
GO:0006614	SRP-dependent cotranslational protein targeting to	9	0.68002854	-0.1674729	-1.5072557	0.72620456	0.65965895	0.60820011	0.733979	0.69213246	0.66813629
GO:0045039	protein import into mitochondrial inner membrane	9	0.67390338	-0.1714024	-1.5426213	0.70272668	0.65064275	0.60509828	0.74547925	0.67618343	0.67163101
GO:0030163	protein catabolic process	15	0.78660205	-0.1042449	-1.5636739	0.81923661	0.76847491	0.70814009	0.85874075	0.79344992	0.77981328
GO:0003333	amino acid transmembrane transport	20	0.83393799	-0.0788662	-1.5773248	0.87870264	0.83091002	0.75669109	0.87542813	0.85447225	0.81389721
GO:0006099	tricarboxylic acid cycle	21	0.84100653	-0.0752006	-1.5792133	0.87327516	0.81508914	0.76704648	0.91626177	0.84368069	0.83834084
GO:0001678	cellular glucose homeostasis	8	0.63469549	-0.1974346	-1.5794767	0.69102983	0.6126021	0.57538539	0.66623515	0.65063533	0.61914616
GO:0006626	protein targeting to mitochondrion	10	0.69477971	-0.1581529	-1.5815288	0.72624022	0.669759	0.62323489	0.76866638	0.69742808	0.69214139
GO:0006464	protein modification process	22	0.84538824	-0.0729438	-1.6047635	0.88185894	0.81816949	0.77179835	0.91723066	0.8494175	0.8413781
GO:0009987	cellular process	15	0.78130447	-0.1071797	-1.6076954	0.8152578	0.75119435	0.69894814	0.87054054	0.7825708	0.78004019
GO:0006855	drug transmembrane transport	44	0.91837285	-0.036981	-1.6271623	0.94948216	0.8750108	0.82700157	1.03530807	0.91148623	0.92531151
GO:0051603	proteolysis involved in cellular protein catabolic proc	23	0.84503331	-0.0731262	-1.6819019	0.88689116	0.81810288	0.76723033	0.91599122	0.85180292	0.83831751
GO:0071805	potassium ion transmembrane transport	30	0.86340996	-0.0637829	-1.9134883	0.90021061	0.84654779	0.79617658	0.91593197	0.87296695	0.8539576
GO:0010044	response to aluminum ion	8	0.57454709	-0.2406744	-1.925395	0.6458473	0.55506762	0.52672842	0.5770853	0.59873944	0.55133223
GO:0043039	tRNA aminoacylation	11	0.65487411	-0.1838422	-2.022264	0.69587079	0.63636967	0.58599319	0.70876234	0.66545553	0.64446094
GO:0006414	translational elongation	24	0.81978058	-0.0863024	-2.071257	0.8543069	0.79555311	0.76067569	0.87359044	0.82440676	0.81518035
GO:0015991	ATP hydrolysis coupled proton transport	26	0.82706293	-0.0824614	-2.1439975	0.86536216	0.79680595	0.74630248	0.90926164	0.83037685	0.82376223
GO:0006887	exocytosis	35	0.862952	-0.0640134	-2.2404676	0.91679302	0.86088717	0.75795825	0.92700858	0.88840044	0.83823254
GO:0006888	ER to Golgi vesicle-mediated transport	18	0.74170619	-0.1297681	-2.3358258	0.77612815	0.72302842	0.66989714	0.80506451	0.74910794	0.73437757
GO:0001522	pseudouridine synthesis	20	0.75860471	-0.1199845	-2.3996894	0.78118107	0.75365356	0.71172015	0.7903681	0.76729387	0.75001394
GO:0042254	ribosome biogenesis	17	0.7223581	-0.1412475	-2.4012067	0.75880781	0.70227997	0.65555162	0.77940149	0.72999693	0.71479921
GO:0009451	RNA modification	22	0.77540321	-0.1104724	-2.4303929	0.80076569	0.7680423	0.72339034	0.81254436	0.78423334	0.76667251
GO:0016036	cellular response to phosphate starvation	3	0.1438662	-0.8420412	-2.5261237	0.14947601	0.14994601	0.12418693	0.15390503	0.14971082	0.13824975
GO:0006352	transcription initiation%2C DNA-dependent	25	0.78045626	-0.1076514	-2.6912858	0.85989658	0.74508966	0.71041734	0.81512828	0.80043741	0.76097389
GO:0009058	biosynthetic process	143	0.95578029	-0.0196419	-2.8087961	1.03774618	0.91925655	0.85460052	1.02362586	0.97670618	0.93530273
GO:0006913	nucleocytoplasmic transport	26	0.77512503	-0.1106282	-2.8763342	0.82652239	0.74323723	0.69748084	0.84250537	0.78377434	0.76657117
GO:0006817	phosphate ion transport	11	0.54255995	-0.2655523	-2.921075	0.56615216	0.54425742	0.49707497	0.56575904	0.55509685	0.53030619
GO:0044267	cellular protein metabolic process	25	0.7616852	-0.1182245	-2.9556121	0.80491192	0.73188364	0.6896088	0.82853122	0.76752972	0.75588519
GO:0006184	GTP catabolic process	44	0.85467021	-0.0682014	-3.0008632	0.91384876	0.82216816	0.76757595	0.92520489	0.86679718	0.84271289
GO:0006096	glycolysis	55	0.87894827	-0.0560367	-3.0820178	0.92775851	0.84633487	0.79282567	0.95873452	0.88611195	0.8718425
GO:0044237	cellular metabolic process	77	0.9038208	-0.0439177	-3.3816606	0.96880226	0.86886781	0.82480459	0.96114609	0.91747539	0.89036942
GO:0045454	cell redox homeostasis	109	0.9297845	-0.0316177	-3.4463292	0.98429401	0.89122273	0.84113437	1.01286772	0.93660301	0.92301563
GO:0006364	rRNA processing	26	0.72777848	-0.1380008	-3.5880205	0.77057214	0.70261628	0.65579126	0.79013209	0.73581012	0.71983451
GO:0006662	glycerol ether metabolic process	34	0.77975365	-0.1080426	-3.6734478	0.83676727	0.75834502	0.69189385	0.84201275	0.79659167	0.76327154
GO:0007165	signal transduction	119	0.93123685	-0.0309398	-3.6818421	0.99908801	0.89609325	0.84460785	0.99455438	0.94619027	0.91651974
GO:0055114	oxidation-reduction process	1193	0.99251688	-0.0032621	-3.8916828	1.06148867	0.97184547	0.89800383	1.04751643	1.01567857	0.96988338
GO:0006952	defense response	206	0.956416	-0.0193532	-3.9867525	1.0366084	0.92992905	0.86806296	0.99993453	0.98182089	0.93166846
GO:0008033	tRNA processing	26	0.63346144	-0.1982798	-5.1552752	0.66670304	0.61204818	0.56019267	0.7044096	0.63879134	0.62817601
GO:0006413	translational initiation	28	0.62715734	-0.2026235	-5.6734577	0.66079011	0.60513238	0.56759228	0.68164091	0.63234918	0.62200813
GO:0007264	small GTPase mediated signal transduction	72	0.82069514	-0.0858181	-6.1789059	0.87472584	0.79244255	0.7297613	0.89682329	0.8325683	0.80899131
GO:0006351	transcription%2C DNA-dependent	79	0.82377421	-0.0841918	-6.6511527	0.86472193	0.79910806	0.74647177	0.8927655	0.83126787	0.81634812
GO:0016567	protein ubiquitination	71	0.79396993	-0.1001959	-7.1139121	0.85731489	0.79301472	0.70469598	0.82945497	0.82453825	0.76453488
GO:0016192	vesicle-mediated transport	91	0.82882163	-0.0815389	-7.4200419	0.87415667	0.80778356	0.74603646	0.89577713	0.84031505	0.81748541
GO:0006396	RNA processing	67	0.7707321	-0.1130966	-7.577469	0.81843578	0.74834803	0.69603931	0.8277359	0.78260769	0.75903671
GO:0006418	tRNA aminoacylation for protein translation	43	0.65853481	-0.1814213	-7.8011143	0.700956	0.64030211	0.58886037	0.71158523	0.66994299	0.6473209
GO:0048544	recognition of pollen	70	0.76065811	-0.1188105	-8.316735	0.82103445	0.7333285	0.70972028	0.78344919	0.77594328	0.74567404
GO:0006508	proteolysis	363	0.94322285	-0.0253857	-9.2150049	1.01027483	0.91451825	0.85956741	0.99665625	0.96120485	0.92557724
GO:0015031	protein transport	98	0.8049761	-0.094217	-9.2332674	0.85964202	0.77472686	0.72039454	0.87517576	0.81608074	0.79402257
GO:0006511	ubiquitin-dependent protein catabolic process	97	0.77835317	-0.1088233	-10.55586	0.81529713	0.7598164	0.70881944	0.83588515	0.78706806	0.76973479
GO:0006810	transport	295	0.91835037	-0.0369916	-10.912521	0.98089122	0.89321541	0.82523191	0.98374049	0.93602732	0.90100724
GO:0055085	transmembrane transport	499	0.94543294	-0.0243693	-12.160265	0.99728661	0.92350424	0.8600308	1.00867218	0.95968662	0.93139097
GO:0008152	metabolic process	875	0.96699409	-0.0145762	-12.754157	1.03543711	0.94095152	0.87557683	1.02496739	0.9870644	0.94733189
GO:0006457	protein folding	168	0.81382392	-0.0894695	-15.030884	0.86015692	0.79527208	0.73396052	0.8736866	0.82707847	0.80078179
GO:0006886	intracellular protein transport	143	0.76441661	-0.1166699	-16.683794	0.80520865	0.7361824	0.6934178	0.83067589	0.76992236	0.75895023
GO:0006355	regulation of transcription%2C DNA-dependent	900	0.93138792	-0.0308694	-27.782459	0.99440105	0.90770808	0.84030243	0.99215383	0.95006624	0.91307681
GO:0006412	translation	294	0.79810295	-0.0979411	-28.794679	0.83468691	0.77298126	0.72463212	0.86781171	0.80324177	0.792997
GO:0006468	protein phosphorylation	1063	0.90896856	-0.0414511	-44.062558	0.97013232	0.88707545	0.82860366	0.95731986	0.92767482	0.89063951

A

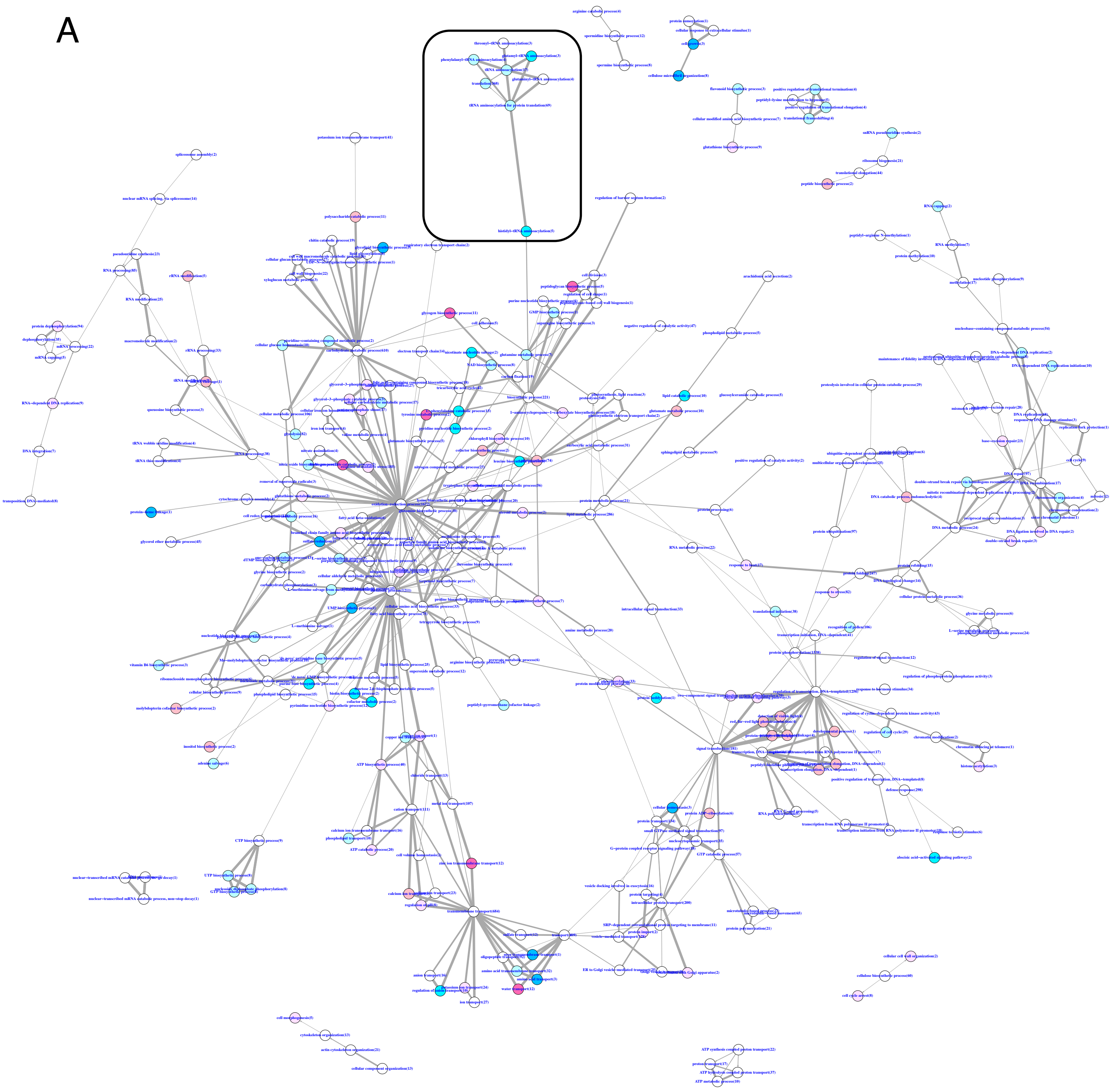

Supplementary Figure S1. Co-occurrence network map of GO-terms in "Biological process" category. The GO terms were mapped on a co-occurrence map by GO enrichment analysis. This figure shows networks of the GO terms with multiple co-occurrence targets. Each connects to the major ones. Vertex circle colors with different brightness indicate average foldchanges of gene expression belonging to a GO term: Red and blue indicates Up- and down- regulation in the four experimental samples, respectively.

B

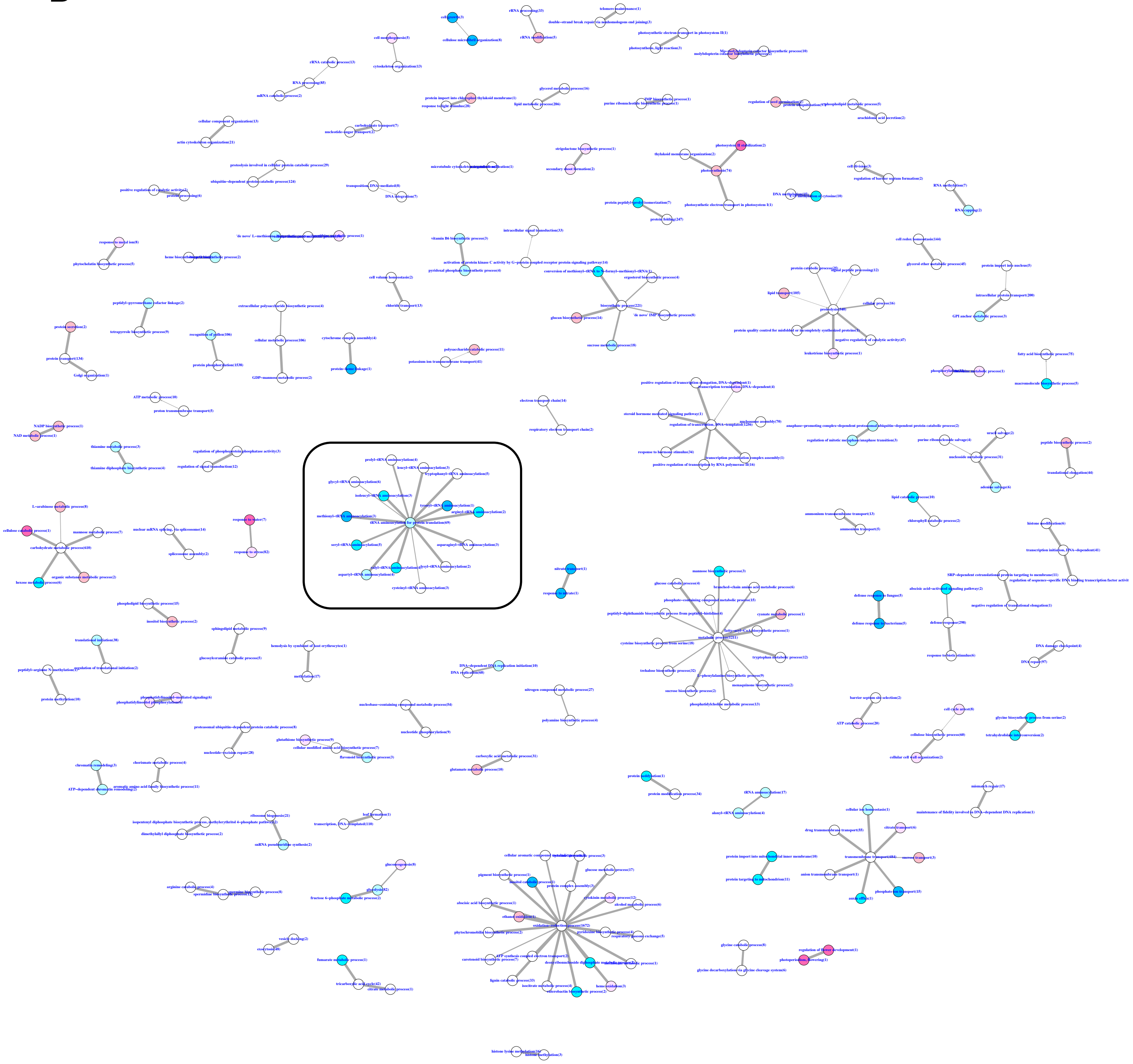

Supplementary Figure S2. Co-occurrence map of pair GO-terms in "Biological process" category. The GO terms were mapped on a co-occurrence map by GO enrichment analysis. This figure shows networks of the GO terms with simple co-occurrence targets. Vertex circle colors with different brightness indicate average foldchanges of gene expression belonging to a GO term: Red and blue indicates Up- and down- regulation in the four experimental samples, respectively.

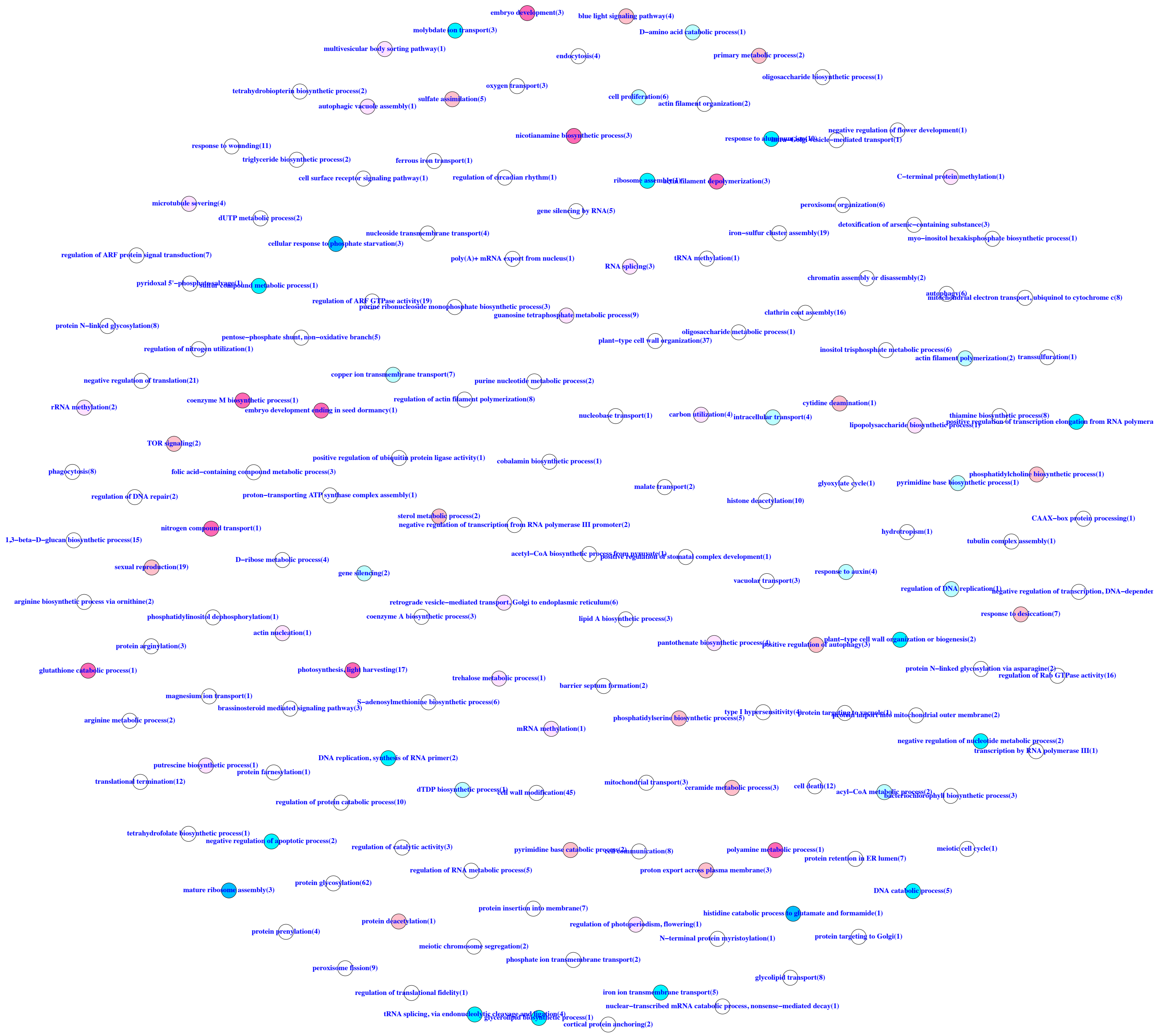

Supplementary Figure S3. Co-occurrence map of orphan GO-terms in "Biological process" category. The GO terms were mapped on a co-occurrence map by GO enrichment analysis. This figure shows GO terms with no connection to any co-occurrence target. Circle colors with different brightness indicate average foldchanges of gene expression belonging to a GO term: Red and blue indicates Up- and down-regulation in the four experimental samples, respectively.

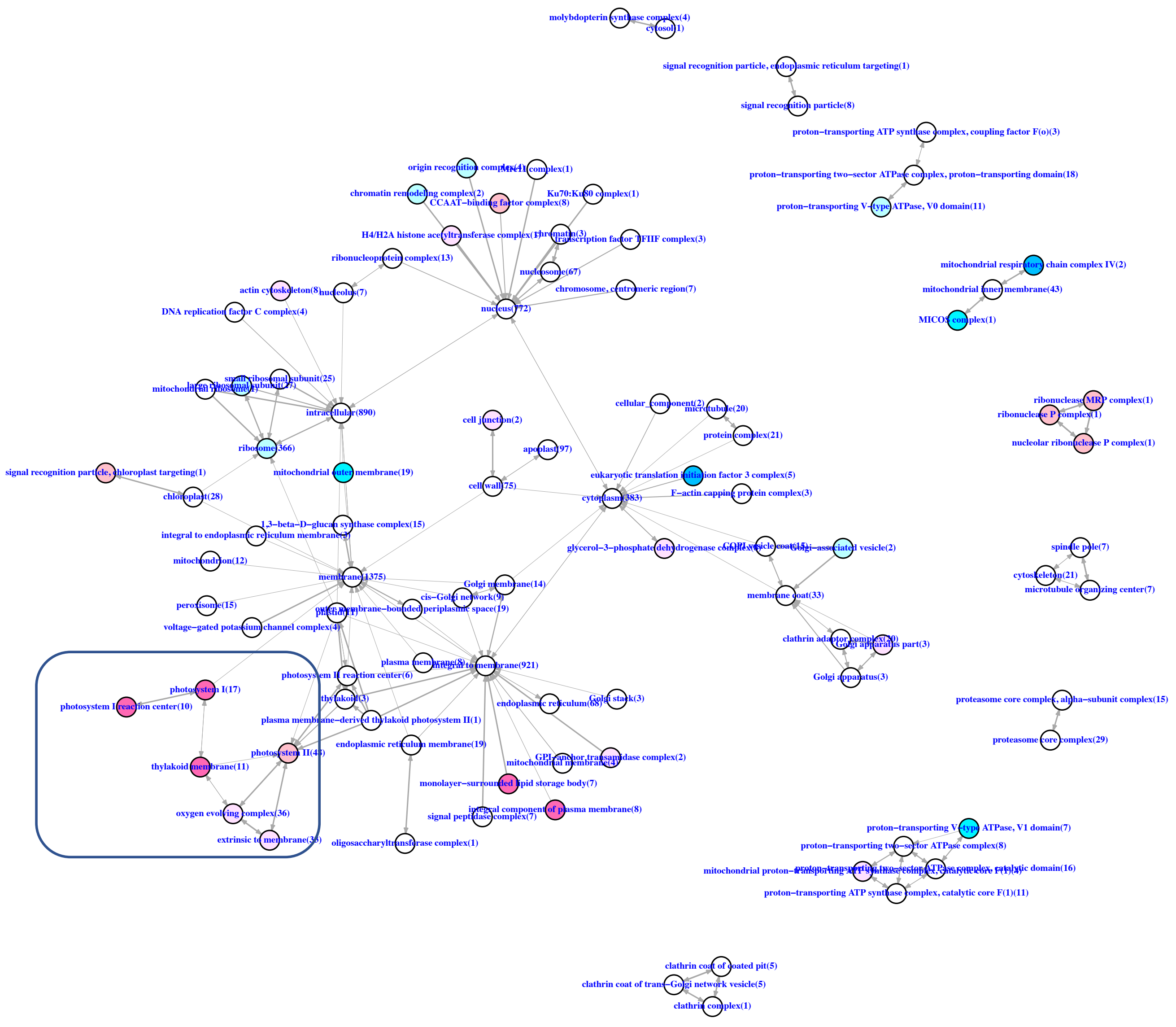

Supplementary Figure S4. Co-occurrence map of GO-terms in "Cellular components" category. The GO terms were mapped on a co-occurrence map by GO enrichment analysis. This figure shows networks of the GO terms with multiple co-occurrence targets. Each connects to the major ones. Vertex circle colors with different brightness indicate average foldchanges of gene expression belonging to a GO term: Red and blue indicates Up- and down- regulation in the four experimental samples, respectively.

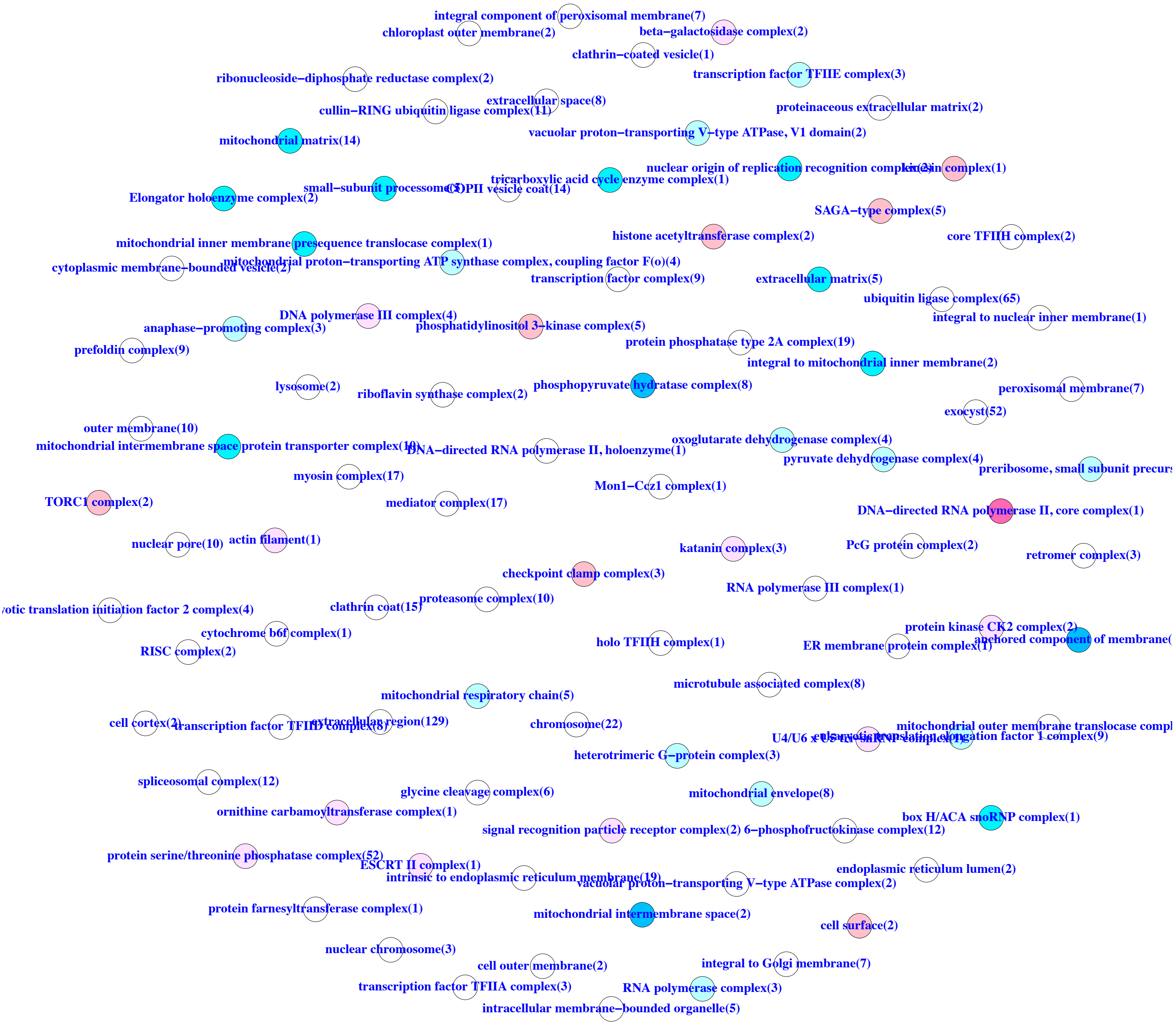

Supplementary Figure S5. Co-occurrence map of orphan GO-terms in "Cellular components" category. The GO terms were mapped on a co-occurrence map by GO enrichment analysis. This figure shows GO terms with no connection to any co-occurrence target. Circle colors with different brightness indicate average foldchanges of gene expression belonging to a GO term: Red and blue indicates Up- and down-regulation in the four experimental samples, respectively.

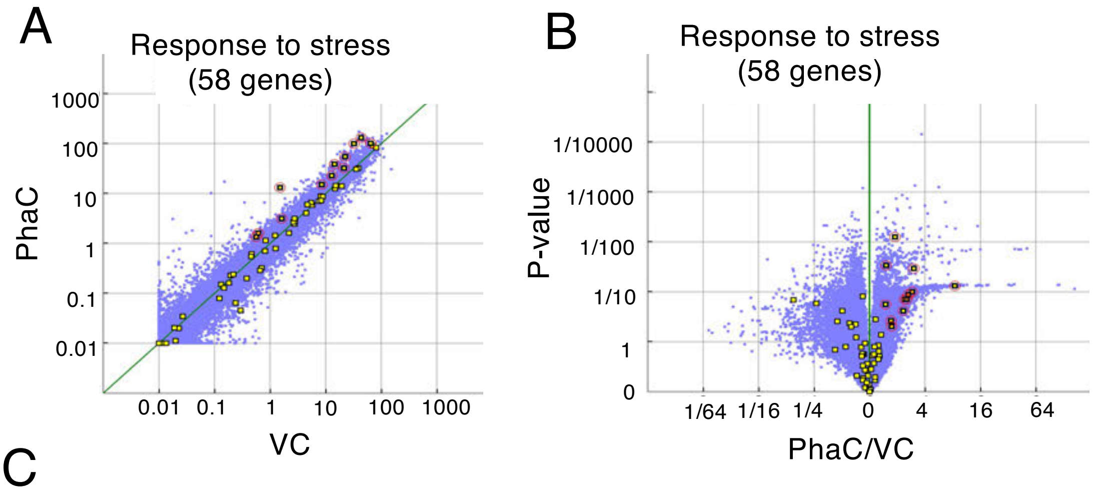

C

Gene ID	annotation	PhaC/VC
Os04g0423800	Peroxidase precursor (EC 1.11.1.7).	19.5
Os07g0677100	Peroxidase.	11.3
Os01g0263300	Peroxidase 72 precursor (EC 1.11.1.7)	4.33
Os03g0368900	Haem peroxidase, plant/fungal/bacterial family protein.	5.63
Os05g0499300	Peroxidase precursor (EC 1.11.1.7).	6.89
Os05g0134700	Haem peroxidase, plant/fungal/bacterial family protein.	8.61
Os01g0294700	Haem peroxidase, plant/fungal/bacterial family protein.	15.0
Os07g0677200	Peroxidase.	6.74
Os01g0327100	Haem peroxidase, plant/fungal/bacterial family protein.	2.58
Os02g0115700	Catalase isozyme A (EC 1.11.1.6) (CAT-A).	2.57
Os04g0689000	Anionic peroxidase precursor.	2.14

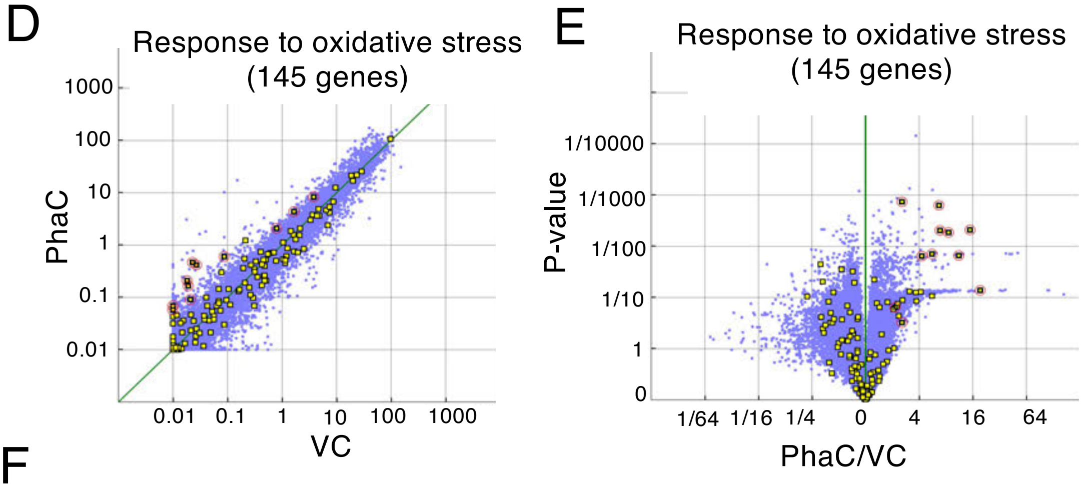

F

Gene ID	annotation	PhaC/VC
Os04g0107900	Heat shock protein 81-1 (HSP81-1) (Heat shock protein 83).	2.36
Os11g0454000	Dehydrin family protein.	2.55
Os05g0355400	Conserved hypothetical protein.	1.74
Os03g0750000	Universal stress protein (Usp) family protein.	1.73
Os01g0303800	Universal stress protein (Usp) family protein.	1.50
Os09g0482600	Heat shock protein 82.	2.65
Os11g0451700	Dehydrin 9.	2.40
Os11g0454200	Dehydrin RAB 16B.	2.92
Os05g0170200	Conserved hypothetical protein.	1.91
Os09g0482400	Heat shock protein 82.	1.53
Os01g0702500	Dehydrin RAB25.	3.05
Os11g0453900	Dehydrin RAB 16D.	8.56

Supplementary Figure S6. Scatter plots of the genes annotated with the GO terms response to stress and response to oxidative stress. (A) Scatter plot of the genes annotated with "response to stress" in the *PhaC* and *VC* lines. The values are the means of the *phaC* expression lines and those of the *VC* lines. (B) Volcano plot of the fold change in expression in the *PhaC* lines relative to the *VC* lines. P values of the *PhaC*/*VC* expression fold changes are shown on the vertical axis. In Panels A and B, 58 genes containing the GO term "response to stress" are indicated by yellow dots. Red-circled dots indicate the representative dots listed in Panel C. (C) List of the genes including the GO term "response to stress". Representative images are shown. (D) Scatter plot and (E) volcano plot of the genes annotated with "response to oxidative stress" in the *PhaC* and *VC* lines. In Panel D and E, 145 genes contained the GO term "response to oxidative stress" and are indicated by yellow dots. Red-circled dots indicate the representative dots listed in Panel F. (F) List of representative genes including the "response to oxidative stress" GO term.

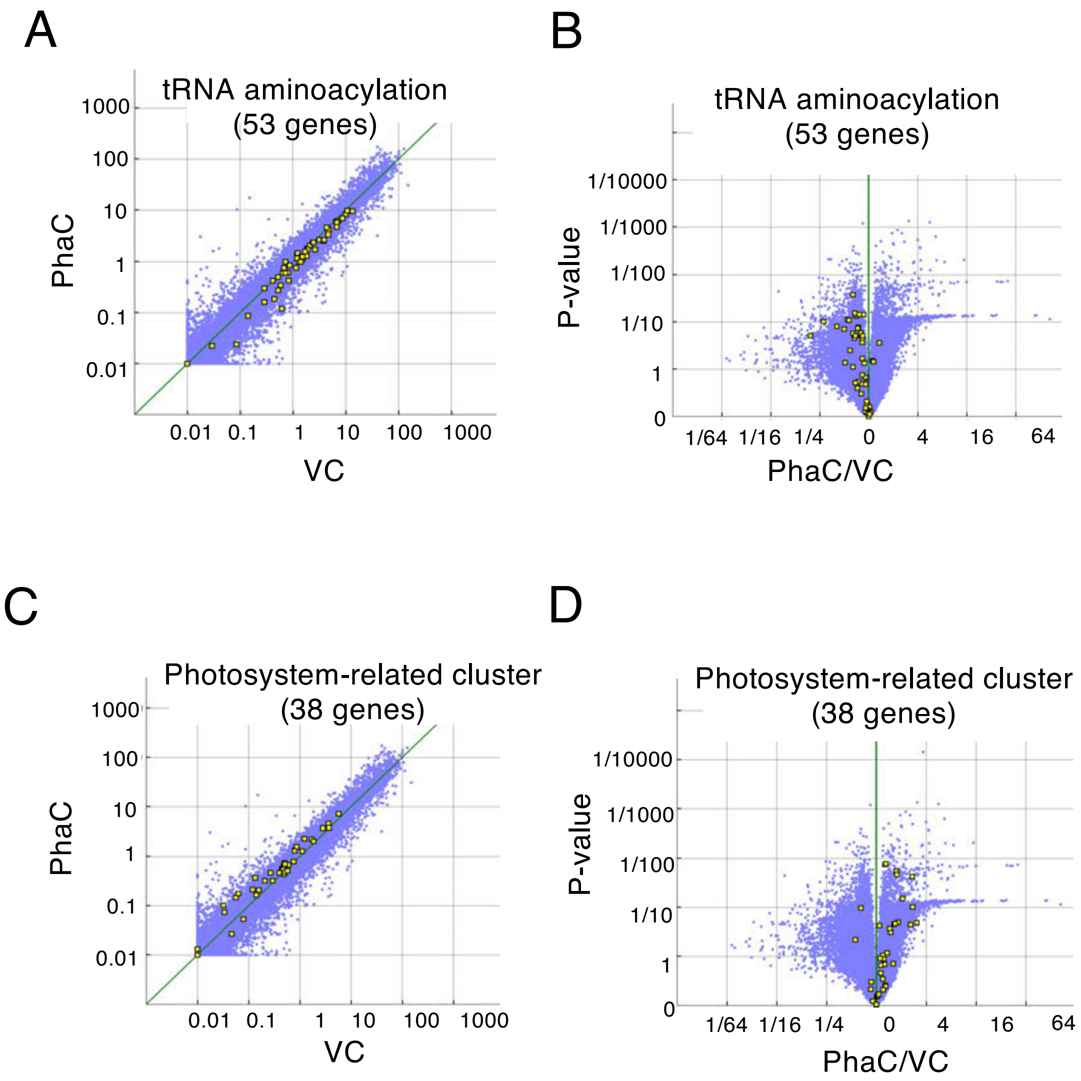

Supplementary Figure S7. Scatter plots of the genes annotated with the GO terms of tRNA aminoacylation and photosystem-related cluster. (A) Scatter plot and (B) volcano plot of the genes annotated with the GO term "tRNA aminoacylation" in the *PhaC* and *VC* lines. Fifty-three genes containing this GO term are indicated by yellow dots. (C) Scatter plot and (D) volcano plot of the genes annotated with " photosystem-related cluster" in the *PhaC* and *VC* lines. Yellow dots show the plots of the 145 genes including the GO term "response to oxidative stress".
